# Cacolides: Sesterterpene Butenolides from a Southern Australian Marine Sponge, *Cacospongia* sp.

**DOI:** 10.3390/md16110456

**Published:** 2018-11-20

**Authors:** Shamsunnahar Khushi, Laizuman Nahar, Angela A. Salim, Robert J. Capon

**Affiliations:** Institute for Molecular Bioscience, University of Queensland, St Lucia, QLD 4072, Australia; s.khushi@imb.uq.edu.au (S.K.); l.nahar@imb.uq.edu.au (L.N.); a.salim@uq.edu.au (A.A.S.)

**Keywords:** *Cacospongia* sp., sesterterpene butenolides, dereplication, GNPS, sesterterpene tetronic acids

## Abstract

Chemical analysis of a marine sponge, *Cacospongia* sp. (CMB-03404), obtained during deep sea commercial fishing activities off the southern coast of Australia, yielded an unprecedented family of sesterterpene α-methyl-γ-hydroxybutenolides, cacolides A–L (**1**–**12**), together with biosynthetically related norsesterterpene carboxylic acids, cacolic acids A–C (**13**–**15**). Structures were assigned on the basis of detailed spectroscopic analysis with comparisons to known natural products and biosynthetic considerations. In addition to revealing new chemical diversity, this study provided a valuable platform for comparing and contrasting the capabilities of the traditional dereplication technologies of HPLC-DAD, HPLC-MS and NMR, with those of the emerging HPLC-MS/MS approach known as global natural products social molecular networking (GNPS), as applied to marine sponge sesterterpene tetronic acids.

## 1. Introduction

Marine sponges (Phyla: Porifera) are a rich source of chemically diverse natural products. Indeed, some natural product structure classes unique to marine sponges have even been instrumental in informing systematic classifications. A case in point are the sesterterpene tetronic acids encountered in the Family: Irciniidae, Genera: *Ircinia*, *Psammocinia* and *Sarcotragus* [1]. Early accounts of Irciniidae sesterterpene tetronic acids include the 1972 report of ircinin-1 and ircinin-2 from *Ircinia oros* [2], and the 1973 report of variabilin from *Ircinia variabilis* [3], with absolute configurations for all three assigned in 1994 using metabolites from Australian *Ircinia* spp. [4]. The rapid pace of marine natural product discovery over the last four decades has included many accounts of Irciniidae sesterterpene tetronic acids, including from Australian *Ircinia* [5,6], *Psammocinia* [6,7,8,9,10] and *Sarcotragus* spp. [10]. As the prevalence of known sesterterpene tetronic acids increased, so did the need for rapid, reliable and cost effective dereplication. Dereplication strategies seek to analyse crude extracts/fractions, to achieve the early detection and de-prioritization of *known* chemistry, such as sponge sesterterpene tetronic acids, to ensure effective use of scientific resources. Modern dereplication strategies, which typically employ hyphenated chromatographic (i.e., HPLC, UPLC), spectroscopic (i.e., UV-vis, DAD, NMR) and/or spectrometric (i.e., MS) technologies, are generally effective at detecting sesterterpene tetronic acids in sponge extracts. As a result, many marine natural products researchers use these strategies to deprioritize such extracts at an early stage in the discovery process, in favour of extracts with a higher probability of returning new chemistry. 

In this report, we describe an unprecedented family of sponge sesterterpenes, cacolides A–L (**1**–**12**) and related metabolites, cacolic acids A–C (**13**–**15**), isolated from a sponge extract initially dereplicated as containing sesterterpene tetronic acids. 

## 2. Results and Discussion

**Figure 1:**
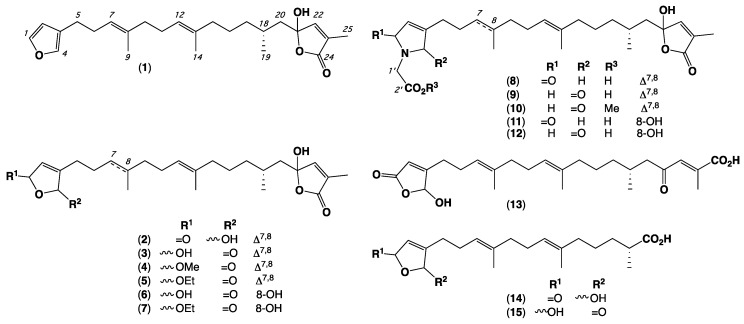
*Cacospongia* sp. (CMB-03404) metabolites **1**–**15**.

**Figure 2:**
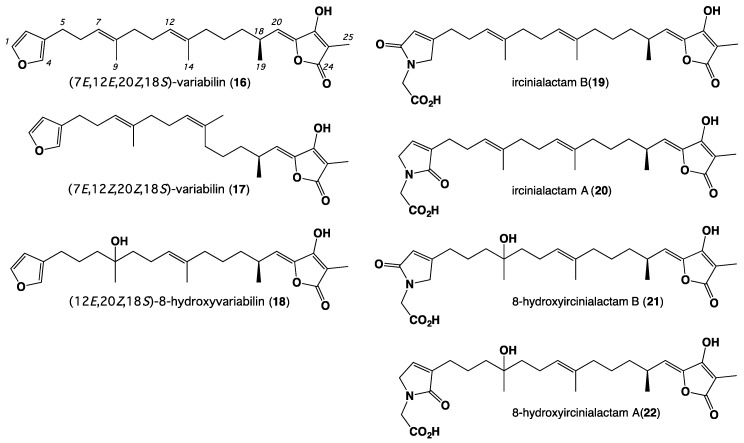
Selected known Irciniidae sesterterpene tetronic acids **16**–**22**.

A portion of the aqueous EtOH extract from a specimen of sponge, *Cacospongia* sp. (CMB-03404), obtained in 2001 during deep-water commercial trawling operations in the Great Australian Bight, was concentrated in vacuo and the residue partitioned to yield H_2_O and *n*-BuOH solubles. The *n*-BuOH solubles were then subjected to sequential solvent trituration to yield *n*-hexane, dichloromethane (DCM), MeOH and H_2_O soluble fractions. Preliminary high performance liquid chromatography diode array detection (HPLC-DAD) analysis revealed a complex array of metabolites concentrated in the DCM and MeOH fractions, although the DAD (UV-vis spectra) data proved uninformative for the purposes of dereplication. By contrast, the ^1^H NMR (CDCl_3_) data revealed resonances initially attributed to commonly encountered sponge sesterterpene tetronic acids, an assignment seemingly reinforced by high performance liquid chromatography mass spectrometry (HPLC-MS) analysis. With the ^1^H NMR and HPLC-MS data suggesting an extract rich in known chemistry and antibacterial screening revealing an absence of growth inhibitory activity against a panel of Gram-positive and -negative pathogens, this extract was initially designated at a low priority for further chemical investigation. However, before leaving this extract to languish in our freezers, we noted a puzzling discrepancy. Sponge extracts rich in sesterterpene tetronic acids typically exhibit significant growth inhibitory activity against Gram-positive bacteria and on occasion against mammalian (i.e., cancer) cells. That the CMB-03404 extract did not exhibit such activity seemed incompatible with the preliminary determination that the extract was rich in sesterterpene tetronic acids. Intrigued by this anomaly, we re-prioritized the extract for detailed chemical analysis. Gratifyingly, solvent extraction followed by trituration and reversed-phase HPLC fractionation yielded a family of unprecedented sesterterpenes (**1**–**15**, Figure 1), with structure elucidation achieved by detailed spectroscopic analysis and biosynthetic considerations and reference to known sesterterpene tetronic acids (**16**–**22**, Figure 2), as outlined below.

**Figure 3:**
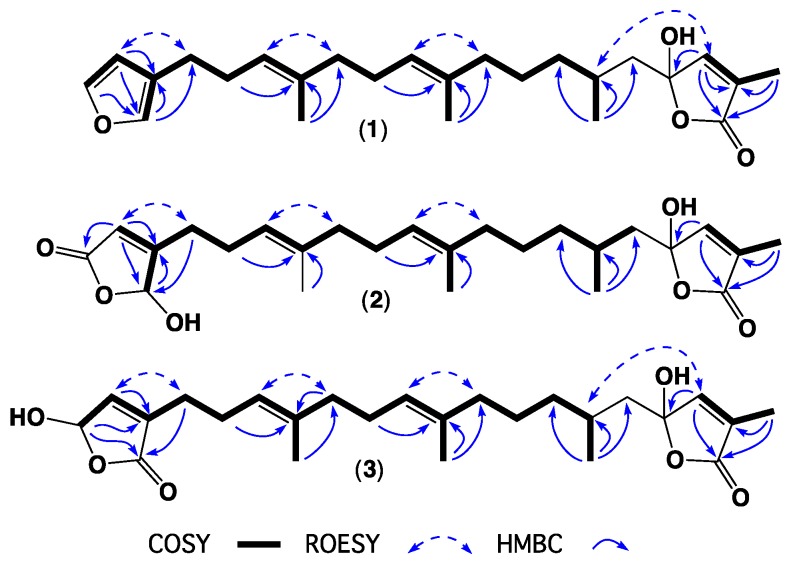
Selected 2D NMR (CDCl_3_) correlations for cacolides A–C (**1**–**3**).

High resolution electrospray ionisation, positive mode mass spectrometry (HRESI(+)MS) analysis of **1** revealed a sodium adduct ion attributed to a molecular formula (C_25_H_36_O_4_, ∆mmu −0.5) consistent with a dihydro analogue of the known sponge metabolite (7*E*,12*E*,20*Z*,18*S*)-variabilin (**16**) (whose planar structure was first reported as variabilin) [3,4,6,10]. Consistent with this hypothesis, the NMR (CDCl_3_) data for **1** (Table 1 and Table 2 and Table S1) displayed resonances for a C-1 to C-19 structure fragment in common with **16**. The *E* Δ^7^ and *E* Δ^12^ configurations were evident from shielded ^13^C NMR chemical shifts for C-9 (δ_C_ 16.2) and C-14 (δ_C_ 16.0), compared to the known *Psammocinia* metabolite (7*E*,12*Z*,20*Z*,18*S*)-variabilin (**17**) (*E* Δ^7^ δ_C_ 16.4, C-9; *Z* Δ^12^ δ_C_ 24.1, C-14) [10], as well as diagnostic rotating-frame nuclear Overhauser effect (ROESY) correlations between H-7 and H_2_-10 and between H-12 and H_2_-15 (Figure 3). Principle differences in the NMR data for **1** compared to **16** were consistent with hydration of *Z* Δ^20^ in **16** to deliver a C-21 acetal (δ_C_ 106.2) in **1** and replacement of the C-21 to C-25 α-methyl-tetronic acid moiety in **16** (i.e., C-22 δ_C_ 166.8) [6], with an α-methyl-γ-hydroxy-γ-substituted butenolide in **1** (i.e., H-22 δ_H_ 6.82; C-22 δ_C_ 147.6) (Figure 3). Based on the above and other 2D NMR correlations (Figure 3), the planar structure for cacolide A (**1**) was assigned as shown (Figure 1).

HRESI(+)MS analysis of **2** revealed a sodium adduct ion attributed to a molecular formula (C_25_H_36_O_6_, ∆mmu −0.7) consistent with an oxidized (+O_2_) analogue of **1**. Comparison of the NMR (CDCl_3_) data for **2** (Table 1 and Table 2 and Table S2) with **1** revealed key differences associated with oxidation of the C-1 to C-4 furan moiety in **1**, to an β-substituted γ-hydroxybutenolide in **2** (C-1 δ_C_ 170.0; H-4 δ_H_ 6.01; C-4 δ_C_ 99.5), with the butenolide regiochemistry confirmed by a heteronuclear multiple bond correlation (HMBC) from H_2_-5 to a C-4 acetal methine (δ_C_ 99.5) (Figure 3). Based on the above and other diagnostic 2D NMR correlations (Figure 3), the planar structure for cacolide B (**2**) was assigned as shown (Figure 1).

HRESI(+)MS analysis of **3** revealed a sodium adduct ion attributed to a molecular formula (C_25_H_36_O_6_, ∆mmu +0.6) consistent with an isomer of **2**. Comparison of the NMR (CDCl_3_) data for **3** (Table 1, Table 2 and Table S3) with **2** revealed a common C-5 to C-25 structure fragment, with the key differences being attributed to an alternate C-1 to C-4 α-substituted γ-hydroxybutenolide regiochemistry (H-1 δ_H_ 6.09; C-1 δ_C_ 97.2; C-4 δ_C_ 172.5), the latter independently confirmed by an HMBC from H_2_-5 to a C-4 carbonyl (δ_C_ 172.5) (Figure 3). Based on the above and other diagnostic 2D NMR correlations (Figure 3), the planar structure for cacolide C (**3**) was assigned as shown (Figure 1). 

**Figure 4:**
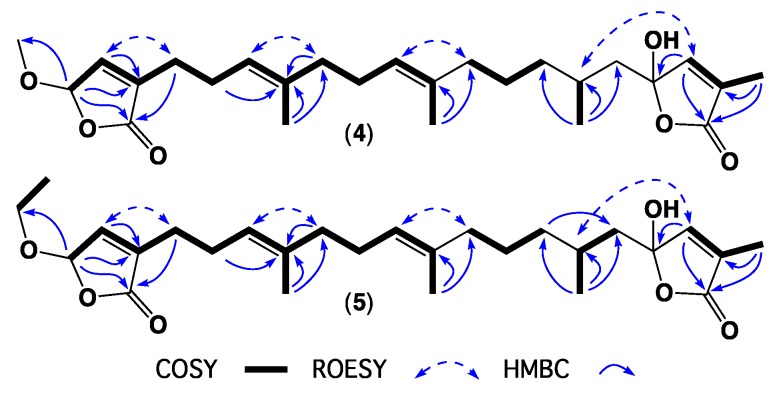
Selected 2D NMR (CDCl_3_) correlations for cacolides D–E (**4**–**5**).

HRESI(+)MS analysis of **4** and **5** revealed sodium adduct ions attributed to molecular formulae (C_26_H_38_O_6_, ∆mmu +0.2 and C_27_H_40_O_6_, ∆mmu −0.8) consistent with methyl and ethyl homologues of **3**, respectively. Comparison of the NMR (CDCl_3_) data for **4** and **5** (Table 1, Table 2 and Table 3, Tables S4 and S5), with **3**, revealed a common carbon skeleton, including a C-1 to C-4 α-substituted γ-hydroxybutenolide terminus, with the key differences being the appearance of 1-OCH_3_ (δ_H_ 3.56; δ_C_ 57.3, OCH_3_) and 1-OCH_2_CH_3_ (δ_H_ 3.73/3.91 and δ_C_ 66.0, OCH_2_CH_3_; δ_H_ 1.27 and δ_C_ 15.2, OCH_2_CH_3_) moieties. The regiochemistry of these alkylations was independently confirmed from HMBC correlations from H-1 to OCH_3_ in **4** and H-1 to OCH_2_CH_3_ in **5** (Figure 4). Based on the above, the planar structures for cacolides D (**4**) and E (**5**) were assigned as shown (Figure 1). 

**Figure 5:**
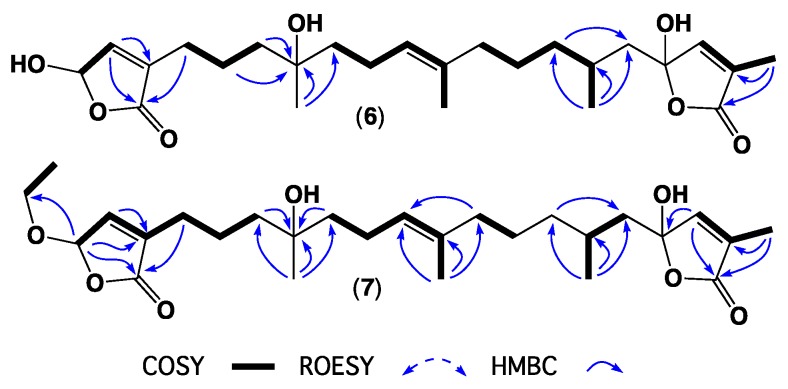
Selected 2D NMR (CDCl_3_) correlations for cacolides F–G (**6**–**7**).

HRESI(+)MS analysis of **6** and **7** revealed sodium adduct ions attributed to molecular formulae (C_25_H_38_O_7_, ∆mmu −1.1 and C_27_H_42_O_7_, ∆mmu +0.5) consistent with hydrated (+H_2_O) homologues of **3** and **5**, respectively. Comparison of the NMR (CDCl_3_) data for **6** and **7** (Table 1, Table 3, Tables S6 and S7), with **3** and **5**, revealed the key differences as hydration of Δ^7^ in **3** (C-7 δ_C_ 122.9; C-8 δ_C_ 136.8) and **5** (C-7 δ_C_ 122.7; C-8 δ_C_ 136.9), to yield a common C-8 3°-OH in **6** (C-7 δ_C_ 41.5; C-8 δ_C_ 73.5) and **7** (C-7 δ_C_ 41.6; C-8 δ_C_ 73.1). The NMR data for **6** and **7** compared favourably with the known *Sarcotragus* metabolite (12*E*,20*Z*,18*S*)-8-hydroxyvariabilin (**18**) [6,11], with the C-1 to C-4 α-substituted γ-hydroxybutenolide regiochemistry confirmed by HMBC correlations from H_2_-5 to C-4 carbonyls (**6** δ_C_ 172.3; **7** δ_C_ 171.9) (Figure 5). Based on the above, the planar structures for cacolides F (**6**) and G (**7**) were assigned as shown (Figure 1). Cacolides E (**5**) and G (**7**) are likely ethanolysis artefacts of **3** and **6**, respectively, induced during prolonged storage in EtOH.

**Figure 6:**
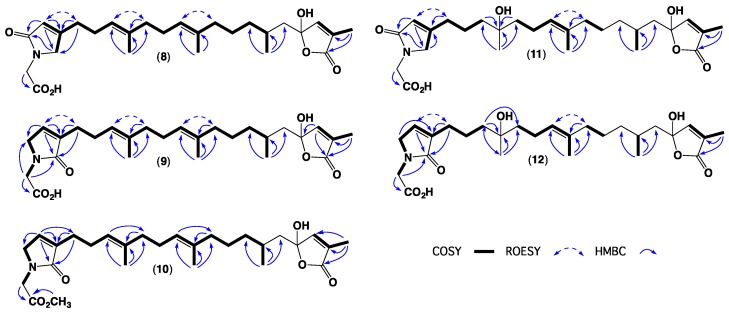
Selected 2D NMR (CDCl_3_) correlations for cacolides H–L (**8**–**12**).

HRESI(+)MS analysis of **8** and **9** revealed sodium adduct ions attributed to isomeric molecular formulae (C_27_H_39_NO_6_, ∆mmu +0.0 and −1.2) consistent with dihydro analogues of the known *Psammocinia* metabolite ircinialactam B (**19**) [10], and *Ircinia/Psammocinia* metabolite ircinialactam A (**20**) [6,10]. Comparison of the NMR (CDCl_3_) data for **8** and **9** (Table 4, Table 5, Tables S8 and S9) with co-metabolites **1**–**5** revealed a common C-5 to C-25 structure fragment. However, whereas **8** possessed a C-1 to C-4 β-substituted glycinyl lactam regiochemistry in common with **19**, the co-metabolite **9** possessed an C-1 to C-4 α-substituted glycinyl lactam regiochemistry in common with **20**. The lactam regiochemistry in **8** was further confirmed by an HMBC from H_2_-5 to a C-4 methylene (δ_C_ 55.7), while that in **9** was confirmed by an HMBC from H_2_-5 to a C-4 carbonyl (δ_C_ 173.3) (Figure 6). Based on the above, the planar structures for cacolides H (**8**) and I (**9**) were assigned as shown (Figure 1).

HRESI(+)MS analysis of **10** revealed a sodium adduct ion attributed to a molecular formulae (C_28_H_43_NO_6_, ∆mmu −0.1) consistent with a methylated analogue of **8** or **9**. Comparison of the 1D and 2D NMR (CDCl_3_) data for **10** (Table 4, Table 5 and Table S10) with **9** supported this hypothesis, including lactam regiochemistry and double bond conformations, with the new OCH_3_ (δ_H_ 3.74; δ_C_ 52.4) positioned on the basis of an HMBC to the adjacent C-2′ carbonyl (Figure 6). Based on the above, the structure for cacolide J (**10**) was assigned as shown (Figure 1).

HRESI(+)MS analysis of **11** and **12** revealed sodium adduct ions attributed to isomeric molecular formulae (C_27_H_41_NO_7_, ∆mmu +1.3 and −1.4) consistent with hydrated analogues of the co-metabolites **8** and **9**. Comparison of the NMR (CDCl_3_) data for **11** and **12** (Table 4, Table 5, Tables S11 and S12), with co-metabolites **8** and **9**, revealed a common C-11 to C-25 structure fragment, with **11** sharing a C-1 to C-5 β-substituted glycinyl lactam moiety in common with **8** and **12** sharing a C-1 to C-5 α-substituted glycinyl lactam in common with **9**. Both **8** and **9** also shared a C-8 3°-OH moiety in common with co-metabolites **6** and **7** and the known *Ircinia* metabolites, 8-hydroxyircinialactams B (**21**) [6] and A (**22**) [6]. Based on the above, plus diagnostic 2D NMR correlations (Figure 6), the planar structures for cacolides K (**11**) and L (**12**) were assigned as shown (Figure 1). 

**Figure 7:**
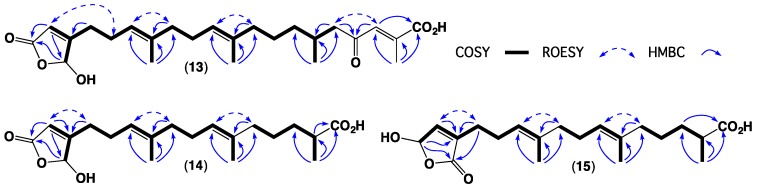
Selected 2D NMR (CDCl_3_) correlations for cacolic acids A–C (**13**–**15**).

HRESI(+)MS analysis of **13** revealed a sodium adduct ion attributed to a molecular formula (C_25_H_36_O_6_, ∆mmu +0.7) isomeric with the co-metabolites **2** and **3**, with comparison of NMR (CDCl_3_) data (Table 4, Table 6 and Table S12) confirming a common C-1 to C-19 structure fragment, inclusive of a C-1 to C-4 β-substituted γ-hydroxybutenolide and *E* Δ^7^ and *E* Δ^12^ moieties. Further NMR comparisons revealed that **13** lacked the characteristic C-21 to C-25 α-methyl-γ-hydroxyl-γ- substituted butenolide moiety common across cacolides A–L (**1**–**12**), in favour of an acyclic isomeric equivalent, featuring a ketone (C-21, δ_C_ 202.9) conjugated to a Δ^22,23^ (C-22, δ_C_ 133.8; C-23, δ_C_ 140.5) bearing methyl (C-25, δ_C_ 14.4) and carboxylic acid (C-24, δ_C_ 170.7) moieties. The proposed C-21 to C-25 terminus was further confirmed by HMBC correlations from H-22 to C-20 and from H_3_-25 to C-22, C-23 and C-24 (Figure 7). An *E* Δ^22^ configuration was tentatively proposed on the basis that the alternate *Z* configuration might be expected to undergo at least partial cyclisation to yield the butenolide moiety common across co-metabolites **1**–**12** (see biosynthetic discussion below). Based on the above, the planar structure for cacolic acid A (**13**) was assigned as shown (Figure 1).

HRESI(+)MS analysis of **14** and **15** revealed a sodium adduct ions attributed to isomeric molecular formulae (C_20_H_30_O_5_, ∆mmu −1.0 and −0.8, respectively) requiring 6 double bond equivalents (DBE). Analysis of the NMR (CDCl_3_) data for **14** (Table 4, Table 6 and Table S14) revealed resonances for a C-1 to C-19 structure fragment in common with **13**, together with a carboxylic acid (C-20 δ_C_ 180.2), accounting for all 6 DBE. Diagnostic 2D NMR correlations confirmed the regiochemistry of the C-1 to C-4 β-substituted γ-hydroxybutenolide moiety (Figure 7) and allowed assembly of the planar structure for cacolic acid B (**14**) as shown (Figure 1). Analysis of the NMR (CDCl_3_) data for **15** (Table 4, Table 6 and Table S14) revealed resonances for a C-1 to C-19 structure fragment in common with **3**, together with a carboxylic acid (C-20 δ_C_ 180.4), accounting for all 6 DBE. Diagnostic 2D NMR correlations confirmed the regiochemistry of the C-1 to C-4 α-substituted γ-hydroxybutenolide moiety (Figure 7) and allowed assembly of the planar structure for cacolic acid C (**15**) as shown (Figure 1).

**Scheme 1:**
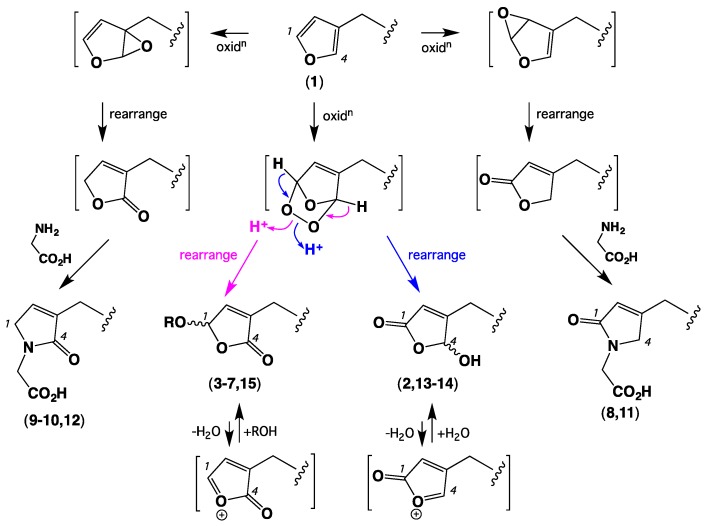
Plausible biosynthetic relationship between the C-1 to C-4 termini in **1**–**15**.

All the metabolites **1**–**15** incorporate chiral sp^3^ centres, at one or both termini and are optically active (negative specific rotations). In an effort to assign absolute configurations we turned our attention to the likely biosynthetic relationship between **1**–**15**. For the C-1 to C-4 termini we propose a relationship (Scheme 1) based around oxidative elaboration of the precursor β-substituted furanyl moiety in cacolide A (**1**). Mono-epoxidations lead to isomeric α,β-unsaturated butyrolactones that in turn form adducts with glycine to yield the regioisomeric glycinyl-lactam pairs **9** and **12** and **8** and **11** [10]. In an alternate oxidative elaboration, 2+4 addition of oxygen to the furan moiety (either stereospecific, or non-stereospecific) generates an intermediate cyclic peroxide, which undergoes alternate rearrangements to yield regioisomeric γ-hydroxybutenolides (**2**, **3**, **6** and **13**–**15**). These butenolides can undergo elimination/addition reactions to both racemize sp^3^ C-1 or C-4 chiral centres and yield OMe (**4**) and OEt (**5** and **7**) adducts, with the latter likely solvolysis artefacts generated during long term freezer storage of *Cacospongia* sp. (CMB-03404) in EtOH. The racemic character of natural sesterterpene γ-hydroxybutenolides is evident in other marine sponge metabolites, including manoalides [12,13,14], luffariellolides [15,16], luffariolides [17], acantholides [18], thorectidaeolides [19] and cacospongionolides [20], and in the case of **1**–**15** ensures that the C-1 to C-4 termini does not contribute to specific rotations.

**Scheme 2:**
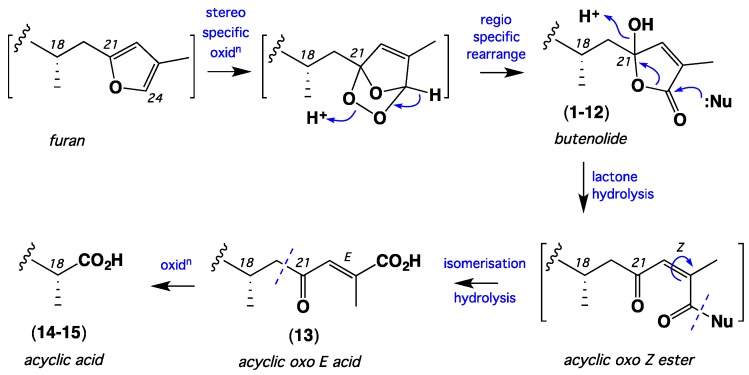
Plausible biosynthetic relationship between the C-17 to C-25 termini in **1**–**15**.

Cacolides A–L (**1**–**12**) incorporate a common C-17 to C-25 terminus bearing chiral centres at C-18 and C-21, with spectroscopic and chromatographic data indicative of a single diastereomer. By comparison, cacolic acids A–C (**13**–**15**) feature an alternate terminus bearing a single C-18 chiral centre. A plausible biosynthetic relationship between the C-17 to C-25 termini in **1**–**15** could see all derived from oxidation of a putative furan precursor (Scheme 2). This would proceed by stereospecific 2 + 4 addition of oxygen to the furan moiety to yield a cyclic peroxide, followed by regiospecific rearrangement to yield the α-methyl-γ-hydroxy-γ-substituted butenolide common to **1**–**12**. Nucleophilic hydrolysis of the lactone heterocyclic would yield an acyclic 21-oxo *Z* Δ^22^ intermediate, which following double bond isomerization and hydrolysis would yield the 21-oxo *E* Δ^22^ carboxylic acid **13**. Further oxidative degradation of **13** would yield **14**–**15**. In this relationship, the configurations about C-18 are preserved across **1**–**15** and about C-21 for **1**–**12**. Comparison of the negative specific rotations for **14**–**15**, with the known model compounds (–)-*R*-2,6-dimethyl-5-heptenoic acid [21,22] and (+)-*S*-2,6-dimethyl-5-heptenoic acid [22], suggests that **14**–**15** and by inference **1**–**13**, possess an 18*R* configuration. The absolute configuration about C-21 in **1**–**12** remains unassigned.

**Figure 8:**
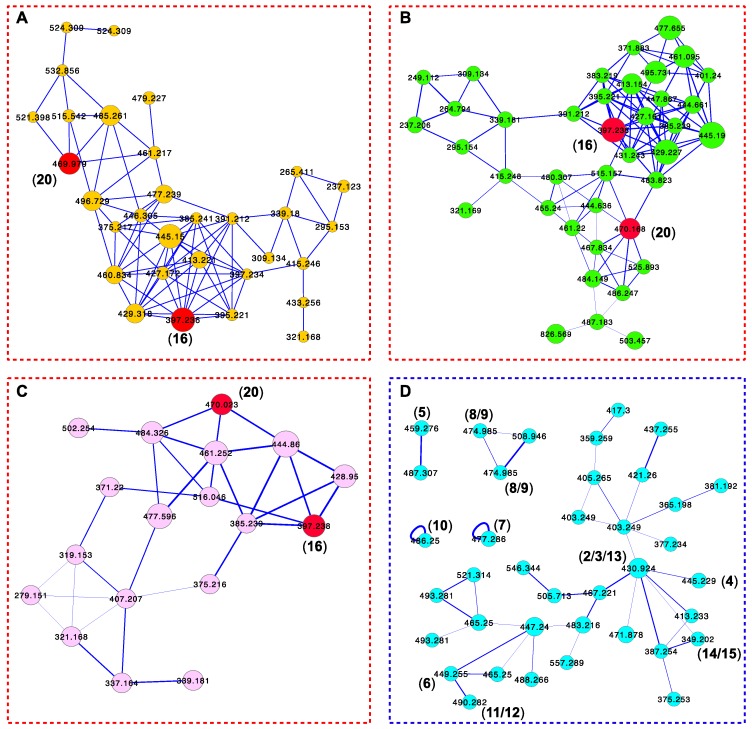
Molecular networking of *n*-BuOH solubles from (**A**) five specimens of *Ircinia spp.*, (**B**) five specimens of *Psammocinia spp.*, (**C**) five specimens of *Sarcotragus spp.* and (**D**) *Cacospongia* sp. (CMB-03404). Red nodes: the two authentic standards, (7*E*,12*E*,20*Z*,18*S*)-variabilin (**16**) and ircinialactam A (**20**).

As predicted from screening of the *Cacospongia* sp. (CMB-03404) *n*-BuOH solubles, none of the pure metabolites **1**–**5** exhibited growth inhibitory activity against the Gram-positive bacterium *Bacillus subtilis*, Gram-negative bacterium *Escherichia coli*, a fungus *Candida albicans*, or two human cancer cell lines (SW620 and NCI-H460) at concentrations up to 30 μM. Furthermore, given the difficulties encountered in using HPLC-DAD, HPLC-MS and ^1^H NMR dereplication on CMB-03404, we elected to investigate the application of Global Natural Product Social Molecular (GNPS) networking [23]. From our in-house library of southern Australian sponges, we acquired ultra-high perfomance liquid chromatography quadrupole time of flight, negative mode mass spectrometry (UPLC-QTOF-(–)MS/MS) data on *n*-BuOH solubles from ×5 specimens each of *Ircinia* spp., *Psammocinia* spp. and *Sarcotragus* spp., along with authentic samples of (7*E*,12*E*,20*Z*,18*S*)-variabilin (**16**) and ircinialactam A (**20**). A GNPS analysis of this data (Figures S3–S5) confirmed that all *Ircinia*, *Psammocinia* and *Sarcotragus* samples displayed a sesterterpene tetronic acid cluster, which incorporated the authentic standards **16** and **20** (and a number of related metabolites) (Figure 8A–C). By contrast, a GNPS analysis performed on *n*-BuOH solubles from *Cacospongia* sp. (CMB-03404) did not reveal a sesterterpene tetronic acid cluster (Figure S6), with the cacolides and cacolic acids dispersed over several clusters (Figure 8D). 

Close inspection of the MS/MS data revealed that, unlike sesterterpene tetronic acids, the butenolides **1**–**15** were prone to the facile loss of CO_2_, with further fragmentations defined by differing degrees of oxygenation and substitution. These observations confirm that, while GNPS can be an effective tool to detect the presence of sesterterpene tetronic acids in crude extracts, it is less useful at detecting and cataloguing cacolides/cacolic acids. Our discovery of cacolides A–L (**1**–**12**) and cacolic acids A–C (**13**–**15**) provides an intriguing chapter in the knowledge of marine sponge natural products and highlights some of the challenges faced in applying traditional and even newer (i.e., GNPS), dereplication technologies.

## 3. Materials and Methods 

### 3.1. General Experimental Procedures 

Optical rotation values ([α]_D_) were measured with a JASCO P-1010 polarimeter (JASCO International Co. Ltd., Tokyo, Japan) in a 100 mm × 2 mm cell at room temperature. NMR spectra were recorded in CDCl_3_ on a Bruker Avance 600 MHz spectrometer (Bruker Pty. Ltd., Alexandria, NSW, Australia) equipped with either a 5 mm PASEL ^1^H/D-^13^C Z-Gradient probe or 5 mm CPTCI ^1^H/^19^F-^13^C/^15^N/DZ-Gradient cryoprobe, controlled by TopSpin 2.1 software (Bruker Pty. Ltd., Alexandria, NSW, Australia). HRESI(+)-MS spectra were obtained on a Bruker micrOTOF mass spectrometer (Bruker Daltonik Pty. Ltd., Preston, VIC, Australia) by direct injection in MeCN at 3 μL/min using sodium formate clusters as an internal calibrant. UPLC-HRMS data were obtained on a quadrupole time-of-flight spectrometer (Agilent 6545 Q-TOF LC/MS) using a Zorbax SB-C_8_ column (150 mm × 2.1 mm, 1.7 μm) (Agilent Technologies Inc., Mulgrave, VIC, Australia) with a linear gradient of 10–100% CH_3_CN (with constant 0.1% formic acid as a modifier) at 0.45 mL/min over 6.0 min. HPLC-DAD-ESIMS data were acquired on an Agilent 1100 series separation module equipped with an Agilent 1100 series HPLC/MSD mass detector and diode array multiple wavelength detector (Agilent Technologies Inc., Mulgrave, VIC, Australia). Semi-preparative and preparative HPLCs were performed using Agilent 1100 series HPLC instruments with corresponding detectors, fraction collectors and software inclusively. All solvents used for HPLC separation and purification were chromatographic grade.

### 3.2. Collection and Taxonomy

Specimen CMB-03404 was collected in 2001 as by-catch during commercial fisheries operations (bottom trawling of Lucky S vessel) in the Great Australian Bight. The specimen was immediately frozen and transported at 0 °C to the laboratory, where it was thawed, documented, diced and stored in EtOH at −30 °C prior to chemical investigation. The specimen was taxonomically classified as a *Cacospongia* sp. (Class Demospongiae, Order Dictyoceratida, Family Thorectidae). 

### 3.3. Extraction and Fractionation

A portion of the aqueous EtOH extract of *Cacospongia* sp. (CMB-03404) was decanted, concentrated in vacuo and the residue (4.5 g) partitioned between *n*-BuOH and H_2_O. The *n*-BuOH soluble material was concentrated in vacuo (1.2 g) *and* sequentially triturated and concentrated in vacuo to yield *n*-hexane (6.2 mg), DCM (550 mg), MeOH (500 mg) and H_2_O (31.8 mg) soluble materials. Following HPLC-DAD, UPLC-QTOF and ^1^H NMR (CDCl_3_) analysis, the DCM soluble material was selected for preparative HPLC (Agilent Zorbax SB-C_8_ 5 μm, 21.2 mm × 150 mm column, gradient elution at 20 mL/min from 60% H_2_O/MeCN to 100% MeCN over 40 min, with an isocratic 0.01% TFA modifier) to yield 12 fractions (I-XII). Fraction IX was determined to be pure cacolide I (**9**) (*t*_R_ = 16.7 min; 7.6 mg; 0.63%). The remaining fractions were subjected to preparative HPLC fractionations using an Agilent Zorbax SB-C_8_ 5 μm, 21.2 mm × 150 mm column, with 20 mL/min isocratic elution. Fraction II (isocratic 37% MeCN/H_2_O and 0.01% TFA over 15 min) yielded cacolide K (**11**) (*t*_R_ = 13.1 min; 0.9 mg; 0.08%) and cacolide L (**12**) (*t*_R_ = 14.5 min; 2.3 mg; 0.19%). Fraction III (isocratic 35% MeCN/H_2_O and 0.01% TFA over 36 min) yielded cacolide F (**6**) (*t*_R_ = 31.9 min; 1.6 mg; 0.13%). Fraction V (isocratic 45% MeCN/H_2_O and 0.01% TFA over 30 min) yielded cacolic acid B (**14**) (*t*_R_ = 17.3 min; 1 mg; 0.08%), cacolic acid C (**15**) (*t*_R_ = 18.7 min; 0.6 mg; 0.06%) and cacolide H (**8**) (*t*_R_ = 22.2 min; 3.6 mg; 0.3%). Fraction VI (isocratic 60% MeCN/H_2_O and 0.01% TFA over 28 min) yielded cacolide G (**7**) (*t*_R_ = 15.2 min; 0.8 mg; 0.07%), cacolide B (**2**) (*t*_R_ = 20.2 min; 11.1 mg; 0.93%), cacolide C (**3**) (*t*_R_ = 22.2 min; 11.9 mg; 0.99%) and cacolic acid A (**13**) (*t*_R_ = 26.8 min; 2 mg; 0.17%). Fraction VIII (isocratic 65% MeCN/H_2_O and 0.01% TFA over 15 min) yielded cacolide D (**4**) (*t*_R_ = 13.3 min; 1 mg; 0.08%) and cacolide E (**5**) (*t*_R_ = 16.9 min; 9.3 mg; 0.78%). Fraction X (isocratic 75% MeCN/H_2_O and 0.01% TFA over 15 min) yielded cacolide A (**1**) (*t*_R_ = 13.9 min; 9 mg; 0.75%). Although only present in negligible amounts in the DCM solubles, preparative HPLC (gradient elution from 30% H_2_O/MeCN to 100% MeCN over 25 min, with an isocratic 0.01% TFA modifier) fractionation of the MeOH solubles yielded cacolide J (**10**) (*t*_R_= 14.2 min; 1.6 mg; 0.13%) (see Figures S1 and S2).

### 3.4. Metabolite Characterization 

Cacolide A (**1**). light yellow oil; [α]${}_{D}^{25}$ − 6.7 (*c* 0.60, MeOH); NMR (600 MHz, CDCl_3_) see Table S1 and Figures S4 and S5; HRESIMS *m/z* 423.2511 [M + Na]^+^ (calcd for C_25_H_36_O_4_Na, 423.2506).

Cacolide B (**2**). light yellow oil; [α]${}_{D}^{25}$ − 5.3 (*c* 0.74, MeOH); NMR (600 MHz, CDCl_3_) see Table S2 and Figures S6 and S7; HRESIMS *m/z* 455.2411 [M + Na]^+^ (calcd for C_25_H_36_O_6_Na, 455.2404).

Cacolide C (**3**). light yellow oil; [α]${}_{D}^{25}$ − 6.9 (*c* 0.79, MeOH); NMR (600 MHz, CDCl_3_) see Table S3 and Figures S8–S10; HRESIMS *m/z* 455.2398 [M + Na]^+^ (calcd for C_25_H_36_O_6_Na, 455.2404).

Cacolide D (**4**). light yellow oil; [α]${}_{D}^{25}$ − 20.9 (*c* 0.06, MeOH); NMR (600 MHz, CDCl_3_) see Table S4 and Figures S11 and S12; HRESIMS *m/z* 469.2559 [M + Na]^+^ (calcd for C_26_H_38_O_6_Na, 469.2561).

Cacolide E (**5**). light yellow oil; [α]${}_{D}^{25}$ − 6.9 (*c* 0.62, MeOH); NMR (600 MHz, CDCl_3_) see Table S5 and Figures S13 and S14; HRESIMS *m/z* 483.2725 [M + Na]^+^ (calcd for C_27_H_40_O_6_Na, 483.2717).

Cacolide F (**6**). light yellow oil; [α]${}_{D}^{25}$ − 7.1 (*c* 0.08, MeOH); NMR (600 MHz, CDCl_3_) see Table S6 and Figure S15; HRESIMS *m/z* 473.2521 [M + Na]^+^ (calcd for C_25_H_38_O_7_Na, 473.2510).

Cacolide G (**7**). light yellow oil; [α]${}_{D}^{25}$ − 15.1 (*c* 0.05, MeOH); NMR (600 MHz, CDCl_3_) see Table S7 and Figures S16–S17; HRESIMS *m/z* 501.2817 [M + Na]^+^ (calcd for C_27_H_42_O_7_Na, 501.2823).

Cacolide H (**8**). light yellow oil; [α]${}_{D}^{25}$ − 5.0 (*c* 0.24, MeOH); NMR (600 MHz, CDCl_3_) see Table S8 and Figures S18 and S19; HRESIMS *m/z* 496.2678 [M + Na]^+^ (calcd for C_27_H_39_NO_6_Na, 496.2678).

Cacolide I (**9**). light yellow oil; [α]^25^_D_ − 8.1 (*c* 0.47, MeOH); NMR (600 MHz, CDCl_3_) see Table S9 and Figures S20 and S21; HRESIMS *m/z* 496.2682 [M + Na]^+^ (calcd for C_27_H_39_NO_6_Na, 496.2670).

Cacolide J (**10**). light yellow oil; [α]${}_{D}^{25}$ − 7.6 (*c* 0.11, MeOH); NMR (600 MHz, CDCl_3_) see Table S10 and Figures S22 and S23; HRESIMS *m/z* 510.2827 [M + Na]^+^ (calcd for C_28_H_41_NO_6_Na, 510.2826).

Cacolide K (**11**). light yellow oil; [α]${}_{D}^{25}$ − 4.7 (*c* 0.05, MeOH); NMR (600 MHz, CDCl_3_) see Table S11 and Figure S24; HRESIMS *m/z* 514.2762 [M + Na]^+^ (calcd for C_27_H_41_NO_7_Na, 514.2775).

Cacolide L (**12**). light yellow oil; [α]${}_{D}^{25}$ − 6.9 (*c* 0.15, MeOH); NMR (600 MHz, CDCl_3_) see Table S12 and Figures S25 and S26; HRESIMS *m/z* 514.2789 [M + Na]^+^ (calcd for C_27_H_41_NO_7_Na, 514.2775).

Cacolic acid A (**13**). light yellow oil; [α]${}_{D}^{25}$ − 12.3 (*c* 0.13, MeOH); NMR (600 MHz, CDCl_3_) see Table S13 and Figures S27–S29; HRESIMS *m/z* 455.2397 [M + Na]^+^ (calcd for C_25_H_36_O_6_Na, 455.2404).

Cacolic acid B (**14**). light yellow oil; [α]${}_{D}^{25}$ − 7.5 (*c* 0.07, MeOH); NMR (600 MHz, CDCl_3_) see Table S14 and Figures S30 and S31; HRESIMS *m/z* 373.1995 [M + Na]^+^ (calcd for C_20_H_30_O_5_Na, 373.1985).

Cacolic acid C (**15**). light yellow oil; [α]${}_{D}^{25}$ − 32.2 (*c* 0.04, MeOH); NMR (600 MHz, CDCl_3_) see Table S15 and Figures S32 and S33; HRESIMS *m/z* 373.1993 [M + Na]^+^ (calcd for C_20_H_30_O_5_Na, 373.1985).

### 3.5. Cytotoxicity Assay

Adherent human colorectal (SW620) and lung (NCI-H460) carcinoma cells were cultured in Roswell Park Memorial Institute (RPMI) medium 1640. All cells were cultured as adherent mono-layers in flasks supplemented with 10% foetal bovine serum, l-glutamine (2 mM), penicillin (100 unit/mL) and streptomycin (100 μg/mL), in a humidified 37 °C incubator supplied with 5% CO_2_. Briefly, cells were harvested with trypsin and dispensed into 96-well microtiter assay plates at 2000–5000 cells/well after which they were incubated for 18 h at 37 °C with 5% CO_2_ (to allow cells to attach as adherent mono-layers). Test compounds were dissolved in 20% DMSO in PBS (*v*/*v*) and aliquots (10 μL) applied to cells over a series of final concentrations ranging from 10 nM to 30 μM. After 48 h incubation at 37 °C with 5% CO_2_ an aliquot (20 μL) of (3-(4,5-dimethylthiazol-2-yl)-2,5-diphenyltetrazolium bromide (MTT) in phosphate buffered saline (PBS) (5 mg/mL) was added to each well (final concentration 0.5 mg/mL) and microtiter plates were incubated for a further 4 h at 37 °C with 5% CO_2_. After final incubation, the medium was aspirated and precipitated formazan crystals dissolved in DMSO (100 μL/well). The absorbance of each well was measured at 580 nm with a PowerWave XS Microplate Reader from Bio-Tek Instruments Inc. (Vinooski, VT, USA). IC_50_ values were calculated using Prism 7.0 (GraphPad Software Inc., La Jolla, CA, USA), as the concentration of analyte required for 50% inhibition of cancer cell growth (compared to negative controls). Negative control comprised of 1% aqueous DMSO, while positive control was doxorubicin. All experiments were performed in duplicate.

### 3.6. Antibacterial Assay

The bacterium to be tested was streaked onto a tryptic soy agar plate and incubated at 37 °C for 24 h. One colony was then transferred to fresh tryptic soy broth (15 mL) and the cell density adjusted to 10^4^–10^5^ CFU/mL. The compounds to be tested were dissolved in DMSO and diluted with H_2_O to return 600 µM stock solutions (20% DMSO). The stock solutions were serially diluted to give concentration range of 600 µM to 0.2 µM in 20% DMSO/H_2_O. An aliquot (10 µL) of each dilution was transferred to a 96-well microtiter plate and freshly prepared microbial broth (190 µL) was added to each well. The plates were incubated at 37 °C for 24 h and the optical density of each well was measured spectrophotometrically at 600 nm using POLARstar Omega plate (BMG LABTECH, Offenburg, Germany). Each test compound was screened against the Gram-negative bacterium *Escherichia coli* (ATCC 11775) and the Gram-positive bacterium *Bacillus subtilis* (ATCC 6051). Rifampicin was used as a positive control (40 µg/mL in 10% DMSO). The IC_50_ value was calculated as the concentration of the compound or antibiotic required for 50% inhibition of the bacterial cells using Prism 7.0 from GraphPad Software Inc. (La Jolla, CA, USA).

### 3.7. Antifungal Assay

The fungus to be tested was streaked onto a sabouraud agar plate and incubated at 37 °C for 24 h. One colony was then transferred to fresh sabouraud broth (15 mL) and the cell density adjusted to 10^4^–10^5^ CFU/mL. The compounds to be tested were dissolved in DMSO and diluted with H_2_O to return 600 µM stock solutions (20% DMSO). The stock solutions were serially diluted to give concentration range of 600 µM to 0.2 µM in 20% DMSO/H_2_O. An aliquot (10 µL) of each dilution was transferred to a 96-well microtiter plate and freshly prepared microbial broth (190 µL) was added to each well. The plates were incubated at 37 °C for 24 h and the optical density of each well was measured spectrophotometrically at 600 nm using POLARstar Omega plate (BMG LABTECH, Offenburg, Germany). Each test compound was screened against *Candida albicans* (ATCC 10231). Nystatin was used as a positive control (40 µg/mL in 10% DMSO). The IC_50_ value was calculated as the concentration of the compound or antibiotic required for 50% inhibition of the bacterial cells using Prism 7.0 from GraphPad Software Inc. (La Jolla, CA, USA).

### 3.8. Global Natural Product Social Molecular Networking (GNPS)

A series of GNPS analysis were carried out on UPLC-QTOF-(–)MS/MS data acquired on the *n*-BuOH solubles from; (**A**) *Cacospongia* sp. (CMB-03404); (**B**) *Ircinia* sp. (CMB-01064) known to produce (7*E*,12*E*,20*Z*,18*S*)-variabilin (**16**), (7*E*,12*Z*,20*Z*,18*S*)-variabilin (**17**), (12*E*,20*Z*,18*S*)-8-hydroxyvariabilin (**18**), ircinialactam A (**20**), 8-hydroxyircinialactam B (**21**) and 8-hydroxyircinialactam A (**22**) [6]; (**C**) *Ircinia* sp. (CMB-03363) known to produce **16**–**20** [6]; (**D**) *Ircinia* spp. CMB-01058, CMB-01693 and CMB-02014 that have not been subjected to detailed chemical analysis; (**E**) *Psammocinia* sp. (CMB-03231) known to produce *ent*-ircinialactam C [6]; (**F**) *Psammocinia* sp. (CMB-01018) known to produce **16**–**17** and **20**, as well as ircinialactams C and G-I, ircinialactone A, irciniafuran A, (7*Z*,12*Z*,20*Z*,18*S*)-variabilin and (7*Z*,12*E*,20*Z*,18*S*)-variabilin [10]; (**G**) *Psammocinia* sp. (CMB-03344) known to produce (–)-ircinianin, (–)-ircinianin sulphate, (–)-oxoircinianin, (–)-ircinianin lactam A [7]; (**H**) *Psammocinia* spp. CMB-01757 and CMB-02026 that have not been subjected to detailed chemical analysis; (**I**) *Sarcotragus* spp. CMB-01788, CMB-01848, CMB-02707, CMB-02717 and CMB-03390 that have not been subjected to detailed chemical analysis; and (**J**) authentic standards of (7*E*,12*E*,20*Z*,18*S*)-variabilin (**16**) and ircinialactam A (**20**). To acquire this data, aliquots (1 μL of 100 μg/mL in 100 μL MeOH) of test solutions were analysed on an Agilent 6545 Q-TOF LC/MS equipped with an Agilent 1290 infinity II UPLC system, utilising an Agilent SB-C_8_ 1.8 μm, 2.1 × 50 mm column, with a 0.5 mL/min, 4.5 min gradient elution from 90% H_2_O/CH_3_CN to CH_3_CN, followed by isocratic elution with CH_3_CN for 1 min, with a constant isocratic 0.1% formic acid modifier. UPLC-QTOF-(–)MS/MS data acquired for all samples at a fixed collision energy of 40 eV, were converted from Agilent MassHunter data files (.d) to mzXML file format and transferred to the GNPS server (gnps.ucsd.edu). Molecular networking was performed using the GNPS data analysis workflow using the spectral clustering algorithm and a cosine score of 0.7 and a minimum of 6 matched peaks. The resulting spectral networks were imported into Cytoscape version 3.5.1 (cytoscape.org) [24], where nodes represented parent (-) *m/z* and edge thickness corresponded to cosine scores. Separate GNPS cluster maps were generated for (I) all ×5 *Ircinia* spp. (Figure S3), (II) all ×5 *Psammocinia* spp. (Figure S4) and (III) all ×5 *Sarcotragus* spp. (Figure S5), compared against the authentic standards **16** and **20**; and for (IV) *Cacospongia* sp. (CMB-03404) compared against data for *Ircinia* sp. (CMB-01064) and *Ircinia* sp. (CMB-03363) (Figure S6). In each case sesterterpene tetronic acid and cacolide/cacolic acid clusters are highlighted, as are the nodes for the authentic standards.

## Figures and Tables

**Table 1:** ^13^C (150 MHz) NMR Data for **1**–**7** in CDCl_3._

Position	1	2	3	4	5	6	7
1	142.7	170.0	97.2	102.6	101.6	96.9	101.8
2	111.3	117.6	144.1	142.2	142.5	143.8	142.6
3	125.2	171.1	137.8	138.6	138.3	137.8	138.5
4	139.0	99.5	172.5	171.9	172.0	172.3	171.9
5	25.2	28.0	25.4	25.5	25.5	25.5	25.9
6	28.6	25.3	25.6	25.7	25.7	21.6	21.9
7	123.9	122.6	122.9	122.6	122.7	41.5 ^a^	41.6 ^a^
8	135.9	136.9	136.8	136.8	136.9	73.5	73.1
9	16.2 ^a^	16.1 ^a^	16.1 ^a^	16.3	16.0	26.4	26.9
10	39.9	39.5	39.5	39.7	39.7	41.5 ^a^	41.6 ^a^
11	26.7	25.9	26.1	26.5	26.5	22.9	22.8
12	124.4	123.9	124.2	124.2	124.2	125.1	125.0
13	135.1	135.4	135.3	135.2	135.3	135.2	135.9
14	16.0^a^	16.1 ^a^	16.1 ^a^	16.1	16.2	16.1	16.0
15	39.9	39.8	39.8	39.9	39.8	39.3	39.5
16	25.2	25.1	25.1	25.2	25.2	24.6	25.1
17	37.6	37.5	37.5	37.5	37.3	36.6	37.5
18	28.7	28.6	28.6	29.0	28.7	28.6	28.6
19	21.1	21.1	21.2	21.3	21.2	21.1	21.0
20	44.9	44.7	44.7	44.9	44.8	45.4 ^b^	45.1
21	106.2	107.0	107.0	105.8	106.1	107.5	106.0
22	147.6	148.0	148.0	147.6	147.6	147.6	147.5
23	132.1	131.9	131.8	135.2	132.0	131.7	132.3
24	171.9	172.6	172.6	171.2	172.0	172.0	173.0
25	10.7	10.6	10.6	10.8	10.6	10.7	10.6
1-OMe				57.3			
1-OCH_2_CH_3_					66.0		66.2
1-OCH_2_CH_3_					15.2		15.2

^a^ Resonances with the same superscript within a column are interchangeable, ^b^ Detected from HMBC.

**Table 2:** ^1^H (600 MHz) NMR Data for **1**–**4** in CDCl_3._

Position	1 δ_H_, m (*J* in Hz)	2 δ_H_, m (*J* in Hz)	3 δ_H_, m (*J* in Hz)	4 δ_H_, m (*J* in Hz)
1	7.33, br s		6.09, br s	5.74, br s
2	6.28, br s	5.83, br s	6.84 ^a^, br s	6.78, br s
4	7.21, br s	6.01, br s		
5	2.45, t (7.7)	a 2.52, mb 2.39, m	2.32, t (7.3)	2.34, t (7.3)
6	2.24, dt 7.3, 7.7)	2.30, dt (7.2, 7.2)	2.26, dt (7.2, 7.3)	2.26, dt (7.3, 7.2)
7	5.16 (br t, 7.3)	5.09, br t (7.2)	5.09, br t, (7.2)	5.10, br t (7.2)
9	1.59, s	1.61 (s)	1.59, s	1.61, s
10	2.01, t (7.4)	2.02, t (7.0)	2.01, t (7.0)	2.01, t (7.0)
11	2.07, dt (7.4, 7.4)	2.09, dt (7.0, 7.0)	2.08, dt (7.0, 7.3)	2.08, dt (7.0, 7.3)
12	5.04, br t (7.4)	5.02, br t (7.0)	5.04, br t (7.3)	5.07, br t (7.3)
14	1.57, s	1.56 (s)	1.56, s	1.57, s
15	1.93 ^a^	1.91, m	1.92, m	1.94 ^a^, m
16	1.36, m	1.34, m	1.35, m	1.35, m
17	a 1.30, m b 1.16, m	a 1.29, m b 1.14, m	a 1.30, m b 1.14, m	a 1.30, m b 1.17, m
18	1.64, m	1.64, m	1.63, m	1.65, m
19	0.96, br d (6.7)	0.94, br d (6.5)	0.94, br d (6.3)	0.96, s
20	a 1.94 ^a^ b 1.74, m	a 1.95, m b 1.71, m	a 1.93, m b 1.71, m	a 1.94 ^a^, m b 1.73, m
22	6.82, br s	6.85 (br s)	6.84 ^a^, br s	6.84, br s
25	1.93 ^a^, s	1.90 (s)	1.90, s	1.94 ^a^, s
1-OMe				3.56, s

^a^ Resonances with the same superscript within a column are overlapping signals.

**Table 3:** ^1^H (600 MHz) NMR Data for **5**–**8** in CDCl_3._

Position	5 δ_H_, m (*J* in Hz)	6 δ_H_, m (*J* in Hz)	7 δ_H_, m (*J* in Hz)
1	5.80, br s	6.05, br s	5.82, br s
2	6.78, d (1.4)	6.89, br s	6.81, br s
5	2.33, t (7.3)	2.32, m	2.30, (7.3)
6	2.26, dt (7.2, 7.3)	1.64 ^a^	1.64 ^a^
7	5.10, br t (7.2)	a 1.55 ^b^ b 1.50 ^c^	a 1.64 ^a^ b 1.51 ^b^
9	1.59, s	1.19, s	1.18, s
10	1.99, t (7.0)	a 1.55 ^b^ b 1.50 ^c^	a 1.64 ^a^ b 1.51 ^b^
11	2.06, dt (7.3, 7.0)	2.07, m	2.05, m
12	5.06, br t (7.3)	5.27 (m)	5.15, m
14	1.57, s	1.64 ^a^, br s	1.60, br s
15	1.93 ^a^, m	1.97, m	1.96, m
16	1.36, m	1.41, m	1.36, m
17	a 1.27 ^b^, m b 1.15, m	a 1.31, m b 1.11, m	a 1.33, m b 1.13, m
18	1.67, m	1.70, m	1.66, m
19	0.96, s	0.94, br d (7.0)	0.96, br d (6.8)
20	a 1.93 ^a^ b 1.73, m	^#^	a 1.93 ^c^, m b 1.72, m
22	6.83, br s	6.85, br s	6.83, br s
25	1.93 ^a^, s	1.91, s	1.93 ^c^, s
1-OCH_2_CH_3_	a 3.91, m b 3.73, m		a 3.93, m b 3.74, m
1-OCH_2_CH_3_	1.27 ^b^, t (7.0)		1.27, t (7)

^a–c^ Resonances with the same superscript within a column are overlapping signals; ^#^ Not detected.

**Table 4:** ^13^C (150 MHz) NMR Data for **8**–**15** in CDCl_3._

Position	8	9	10	11	12	13	14	15
1	173.5	52.1	51.7	173.2	52.1	171.4	171.4	97.2
2	121.0	136.9	136.0	121.5	136.9	118.0	118.0	143.9
3	162.3	139.2	139.9	161.8	139.4	169.5	169.5	138.6
4	55.7	173.3	173.1	55.6	173.0	99.3	99.3	172.3
5	30.0	25.9	26.3	30.3	26.5	27.9	27.9	25.3
6	26.0	26.0	26.1	22.3	22.3	25.3	25.3	25.6
7	122.8	123.7	124.1	41.5 ^a^	41.5 ^a^	122.6	122.8	123.0
8	136.8	135.9	135.6	72.7	73.4	137.3	137.0	137.3
9	16.2	16.1	16.1	27.0	26.8	16.2	16.1	16.2
10	39.6	39.5	39.6	41.5 ^a^	41.5 ^a^	39.4	39.4	39.6
11	26.2	26.1	25.9	23.0	22.8	26.1	25.8	26.1
12	124.1	124.3	124.2	125.0	125.1	124.1	124.1	124.4
13	135.3	135.4	135.2	134.8	135.4	135.5	135.2	135.6
14	16.1	16.0	16.0	16.1	15.9	16.1	16.1	16.2
15	39.8	39.8	39.9	39.5	39.4	39.7	39.5	39.6
16	25.1	25.1	25.1	24.7	24.5	25.2	25.6	25.6
17	37.4	37.0	37.8	37.1	37.0	36.4	33.4	33.6
18	28.6	29.1	28.8	28.6	28.5	29.7	39.2	39.1
19	21.1	21.1	21.4	21.3	21.1	20.1	17.2	17.3
20	44.8	44.8	44.9	44.9 ^b^	45.2	52.6	180.2	180.4
21	107.5	107.5	^#^	^#^	107.8	202.9		
22	148.0	148.0	148.1	147.5	148.1	133.8		
23	131.7	131.7	132.0	132.0	131.7	140.5		
24	172.4	171.9	175.5	172.0	172.4	170.7		
25	10.8	10.6	10.7	10.6	10.8	14.4		
1′	43.7	44.1	43.8	44.1	44.4			
2′	171.7	172.6	170.3	171.6	171.3			
1-OMe			52.4					

^a^ Resonances with the same superscript within a column are interchangeable, ^b^ Detected from HMBC, ^#^ Not detected.

**Table 5:** ^1^H (600 MHz) NMR Data for **8**–**12** in CDCl_3._

Position	8 δ_H_, m (*J* in Hz)	9 δ_H_, m (*J* in Hz)	10 δ_H_, m (*J* in Hz)	11 δ_H_, m (*J* in Hz)	12 δ_H_, m (*J* in Hz)
**1**		4.03, br s	4.00, br s		4.02, br s
**2**	5.91, br s	6.77, br s	6.74, br s	5.93, br s	6.78, br s
**4**	4.04, br s			4.04, br s	
**5**	2.43, t (7.5)	2.30, t (7.2)	2.32, t (7.2)	2.40, t (7.5)	2.32, t (7.5)
**6**	2.28, m	2.24, dt (7.3, 7.3)	2.25, dt (7.3, 7.3)	1.66 ^a^, m	1.61, m
**7**	5.09, br t (7.0)	5.11, br t (7.3)	5.15, br t (7.3)	a 1.54 ^b^ b 1.52 ^c^	a 1.52 ^a^ b 1.50 ^b^
**9**	1.60, s	1.59, s	1.60, s	1.19, s	1.18, s
**10**	2.02, m	2.01, t (7.0)	2.03, t (7.0)	a 1.54 ^b^ b 1.52 ^c^	a 1.52 ^a^ b 1.50 ^b^
**11**	2.09, m	2.08, dt (7.0, 7.0)	2.10, dt (7.0, 7.0)	2.07, m	2.05, m
**12**	5.04, br t (7.0)	5.04, br t (7.0)	5.06, br t (7.0)	5.15, br t (7.0)	5.14, br t (7.0)
**14**	1.56, br s	1.56, s	1.56, s	1.59, br s	1.59, br s
**15**	1.92, m	1.92, m	1.93, m	1.96, m	1.95 ^c^
**16**	1.36, m	1.36, m	1.37, m	1.39, m	1.39, m
**17**	a 1.30, m b 1.14, m	a 1.27, m b 1.13, m	a 1.28, m b 1.16, m	a 1.32, m b 1.11, m	a 1.33, m b 1.10, m
**18**	1.64, m	1.62, m	1.64, m	1.66 ^a^, m	1.65, m
**19**	0.94, br d (7.0)	0.94, br s	0.95, br s	0.94, br d (7.0)	0.94, br d (7.0)
**20**	a 1.95, m b 1.70, m	a 1.95, m b 1.69, m	a 2.00, m b 1.71, m	^#^	a 1.95 ^c^ b 1.73, m
**22**	6.82, br s	6.83, br s	6.83, br s	6.81, br s	6.83, s
**25**	1.90, s	1.90, s	1.91, s	1.91, s	1.90, s
**1′**	a 4.22, m b 4.18, m	a 4.28, m b 4.25, m	a 4.25, m b 4.21, m	4.21, br s	4.23, s
**1-OMe**			3.74, s		

^a–c^ Resonances with the same superscript within a column are overlapping signals, ^#^ Not detected.

**Table 6:** ^1^H (600 MHz) NMR Data for **13**–**15** in CDCl_3._

Position	13 δ_H_, m (*J* in Hz)	14 δ_H_, m (*J* in Hz)	15 δ_H_, m (*J* in Hz)
**1**			6.09, br s
**2**	5.85, br s	5.88, br s	6.84, br s
**4**	5.99, br s	6.01, br	
**5**	2.46, m	2.47 ^a^	2.32, t (7.3)
**6**	2.32, m	2.33, m	2.25, dt (7.3, 7.2)
**7**	5.09, br t (7.3)	5.10, br t (7.3)	5.09 br t (7.2)
**9**	1.59, br s	1.64 ^b^, br s	1.64 ^a^, s
**10**	2.03 ^a^	2.06, m	2.02, br t (7.0)
**11**	2.09 (m)	2.12, m	2.09, dt (7.2, 7.0)
**12**	5.03, br t (7.0)	5.06, br t (7.0)	5.04, br t (7.2)
**14**	1.57, br s	1.59, br s	1.57, s
**15**	1.94, m	1.98, m	1.96, m
**16**	1.37, m	1.42 ^c^	1.42 ^b^
**17**	a 1.25, m b 1.15, m	a 1.64 ^b^ b 1.42 ^c^	a 1.64 ^a^ b 1.42 ^b^
**18**	2.03 ^a^	2.47 ^a^	2.47, m
**19**	0.91, br d (6.7)	1.19, br d (7.2)	1.17, br d (7.0)
**20**	a. 2.53, m b. 2.35, m		
**22**	7.11, br s		
**25**	2.20, s		

^a–c^ Resonances with the same superscript within a column are overlapping signals.

## Supplementary Materials

The following are available online at http://www.mdpi.com/1660-3397/16/11/456/s1, HPLC chromatogram and fractionation scheme, full molecular network (GNPS) analyses, tabulated 1D and 2D NMR data, annotated 1D NMR and selected 2D NMR spectra and bioassay data (pdf).
