# Supplementary material for: Cacolides: Sesterterpene Butenolides from a Southern Australian Marine Sponge, Cacospongia sp
Source: Mar Drugs. 2018 Nov 20;16(11):456. doi: 10.3390/md16110456 (PMC6266489; doi:10.3390/md16110456)
Supplement: Supplementary file 1 [file marinedrugs-16-00456-s001.zip › marinedrugs-386982-SI.pdf]

Mar. Drugs 2018, 16, 456; doi:10.3390/md16110456

SUPPORTING INFORMATION

Cacolides: Sesterterpene butenolides from a southern Australian marine sponge, *Cacospongia* sp.

Shamsunnahar Khushi, Laizuman Nahar, Angela Salim, and Robert J. Capon*,

Institute for Molecular Bioscience, The University of Queensland, St Lucia, QLD 4072, Australia

List of tables

Table S1. NMR (600 MHz, $CDCl_3$) data for cacolide A (**1**).. 9
Table S2. NMR (600 MHz,$CDCl_3$) data for cacolide B (**2**).. 10
Table S3. NMR (600 MHz,$CDCl_3$) data for cacolide C (**3**).. 11
Table S4. NMR (600 MHz,$CDCl_3$) data for cacolide D (**4**).. 12
Table S5. NMR (600 MHz,$CDCl_3$) data for cacolide E (**5**).. 13
Table S6. NMR (600 MHz,$CDCl_3$) data for cacolide F (**6**).. 14
Table S7. NMR (600 MHz,$CDCl_3$) data for cacolide G (**7**).. 15
Table S8. NMR (600 MHz,$CDCl_3$) data for cacolide H (**8**).. 16
Table S9. NMR (600 MHz,$CDCl_3$) data for cacolide I (**9**).. 17
Table S10. NMR (600 MHz,$CDCl_3$) data for cacolide J (**10**).. 18
Table S11. NMR (600 MHz,$CDCl_3$) data for cacolide K (**11**).. 19
Table S12. NMR (600 MHz,$CDCl_3$) data for cacolide L (**12**).. 20
Table S13. NMR (600 MHz,$CDCl_3$) data for cacolic acid A (**13**).. 21
Table S14. NMR (600 MHz,$CDCl_3$) data for cacolic acid B (**14**).. 22
Table S15. NMR (600 MHz,$CDCl_3$) data for cacolic acid C (**15**).. 23

List of figures

Figure S1. Fractionation scheme of CMB-03404.. 4
Figure S2. HPLC-DAD (210 nm) chromatogram (C8 column) of DCM partition of the crude extract CMB-03404 {inset: UV spectra for compounds **1-15**}.. 4
Figure S3. Molecular networking for five *Ircinia sp.* (CMB-01064, CMB-03363, CMB-01058, CMB-01693, CMB-02014) and two authentic standards (7*E*,12*E*,20*Z*,18*S*)-variabilin (**16**), ircinialactam A (**20**).. 5
Figure S4. Molecular networking for five *Psammocinia sp.* (CMB-03231, CMB-01018, CMB-03344, CMB-01757, CMB-02026) and two authentic standards (7*E*,12*E*,20*Z*,18*S*)-variabilin (**16**), ircinialactam A (**20**).. 6
Figure S5. Molecular networking for five *Sarcotragus sp.* (CMB-01788, CMB-01848, CMB-02707, CMB-002717, CMB-03390) and two authentic standards (7*E*,12*E*,20*Z*,18*S*)-variabilin (**16**), ircinialactam A (**20**).. 7
Figure S6. Molecular networking for two *Ircinia sp.* (CMB-01064, CMB-03363), *Cacospongia sp.* (CMB-03404) and two authentic standards (7*E*,12*E*,20*Z*,18*S*)-variabilin (**16**), ircinialactam A (**20**).. 8
Figure S7. ^{1}H NMR (600 MHz, $CDCl_3$) spectrum of cacolide A (**1**).. 24
Figure S8. ^{13}C NMR (150 MHz, $CDCl_3$) spectrum of cacolide A (**1**).. 24
Figure S9. ^{1}H NMR (600 MHz, $CDCl_3$) spectrum of cacolide B (**2**).. 25
Figure S10. ^{13}C NMR (150 MHz, $CDCl_3$) spectrum of cacolide B (**2**).. 25
Figure S11. ^{1}H NMR (600 MHz, $CDCl_3$) spectrum of cacolide C (**3**).. 26
Figure S12. ^{13}C NMR (150 MHz, $CDCl_3$) spectrum of cacolide C (**3**).. 26
Figure S13. HSQC NMR ($CDCl_3$) spectrum of cacolide C (**3**).. 27
Figure S14. ^{1}H NMR (600 MHz, $CDCl_3$) spectrum of cacolide D (**4**).. 28
Figure S15. ^{13}C NMR (150 MHz, $CDCl_3$) spectrum of cacolide D (**4**).. 28
Figure S16. ^{1}H NMR (600 MHz, $CDCl_3$) spectrum of cacolide E (**5**).. 29
Figure S17. ^{13}C NMR (150 MHz, $CDCl_3$) spectrum of cacolide E (**5**).. 29
Figure S18. ^{1}H NMR (600 MHz, $CDCl_3$) spectrum of cacolide F (**6**).. 30
Figure S19. ^{1}H NMR (600 MHz, $CDCl_3$) spectrum of cacolide G (**7**).. 31

Figure S20. ^{13}C NMR (150 MHz, $CDCl_3$) spectrum of cacolide G (**7**).. 31
Figure S21. ^{1}H NMR (600 MHz, $CDCl_3$) spectrum of cacolide H (**8**).. 32
Figure S22. ^{13}C NMR (150 MHz, $CDCl_3$) spectrum of cacolide H (**8**).. 32
Figure S23. ^{1}H NMR (600 MHz, $CDCl_3$) spectrum of cacolide I (**9**).. 33
Figure S24. ^{13}C NMR (150 MHz, $CDCl_3$) spectrum of cacolide I (**9**).. 33
Figure S25. ^{1}H NMR (600 MHz, $CDCl_3$) spectrum of cacolide J (**10**).. 31
Figure S26. ^{13}C NMR (150 MHz, $CDCl_3$) spectrum of cacolide J (**10**).. 31
Figure S27. ^{1}H NMR (600 MHz, $CDCl_3$) spectrum of cacolide K (**11**).. 35
Figure S28. ^{1}H NMR (600 MHz, $CDCl_3$) spectrum of cacolide L (**12**).. 36
Figure S29. ^{13}C NMR (150 MHz, $CDCl_3$) spectrum of cacolide L (**12**).. 36
Figure S30. ^{1}H NMR (600 MHz, $CDCl_3$) spectrum of cacolic acid A (**13**).. 37
Figure S31. ^{13}C NMR (150 MHz, $CDCl_3$) spectrum of cacolic acid A (**13**).. 37
Figure S32. HSQC NMR (600 MHz, $CDCl_3$) spectrum of cacolic acid A (**13**).. 38
Figure S33. ^{1}H NMR (600 MHz, $CDCl_3$) spectrum of cacolic acid B (**14**).. 39
Figure S34. ^{13}C NMR (150 MHz, $CDCl_3$) spectrum of cacolic acid B (**14**).. 39
Figure S35. ^{1}H NMR (600 MHz, $CDCl_3$) spectrum of cacolic acid C (**15**).. 40
Figure S36. ^{13}C NMR (150 MHz, $CDCl_3$) spectrum of cacolic acid C (**15**).. 40
Figure S37. Antimicrobial activity of cacolides A-I (**1-9**), K-L (**11-12**) and cacolic acids A-C (**13-15**).................. 41
Figure S38. Cytotoxic activity of cacolides A-I (**1-9**), K-L (**11-12**) and cacolic acids A-C (**13-15**)..................... 42
Figure S39. HR-ESIMS data for cacolide A (**1**).. 43
Figure S40. HR-ESIMS data for cacolide B (**2**).. 43
Figure S41. HR-ESIMS data for cacolide C (**3**).. 44
Figure S42. HR-ESIMS data for cacolide D (**4**).. 44
Figure S43. HR-ESIMS data for cacolide E (**5**).. 45
Figure S44. HR-ESIMS data for cacolide F (**6**).. 45
Figure S45. HR-ESIMS data for cacolide G (**7**).. 46
Figure S46. HR-ESIMS data for cacolide H (**8**).. 46
Figure S47. HR-ESIMS data for cacolide I (**9**).. 47
Figure S48. HR-ESIMS data for cacolide J (**10**).. 48
Figure S49. HR-ESIMS data for cacolide K (**11**).. 49
Figure S50. HR-ESIMS data for cacolide L (**12**).. 49
Figure S51. HR-ESIMS data for cacolic acid A (**13**).. 50
Figure S52. HR-ESIMS data for cacolic acid B (**14**).. 50
Figure S53. HR-ESIMS data for cacolic acid C (**15**).. 51

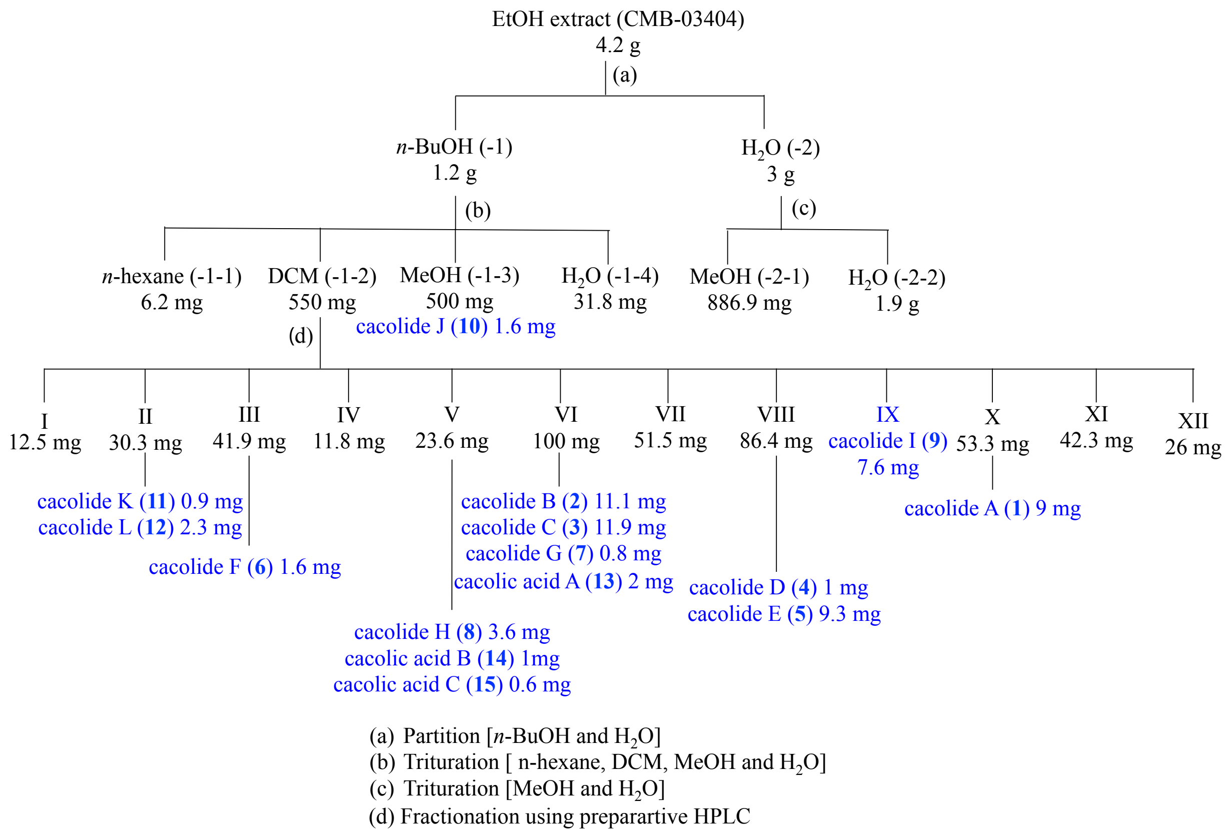

Figure S1. Fractionation scheme of CMB-03404

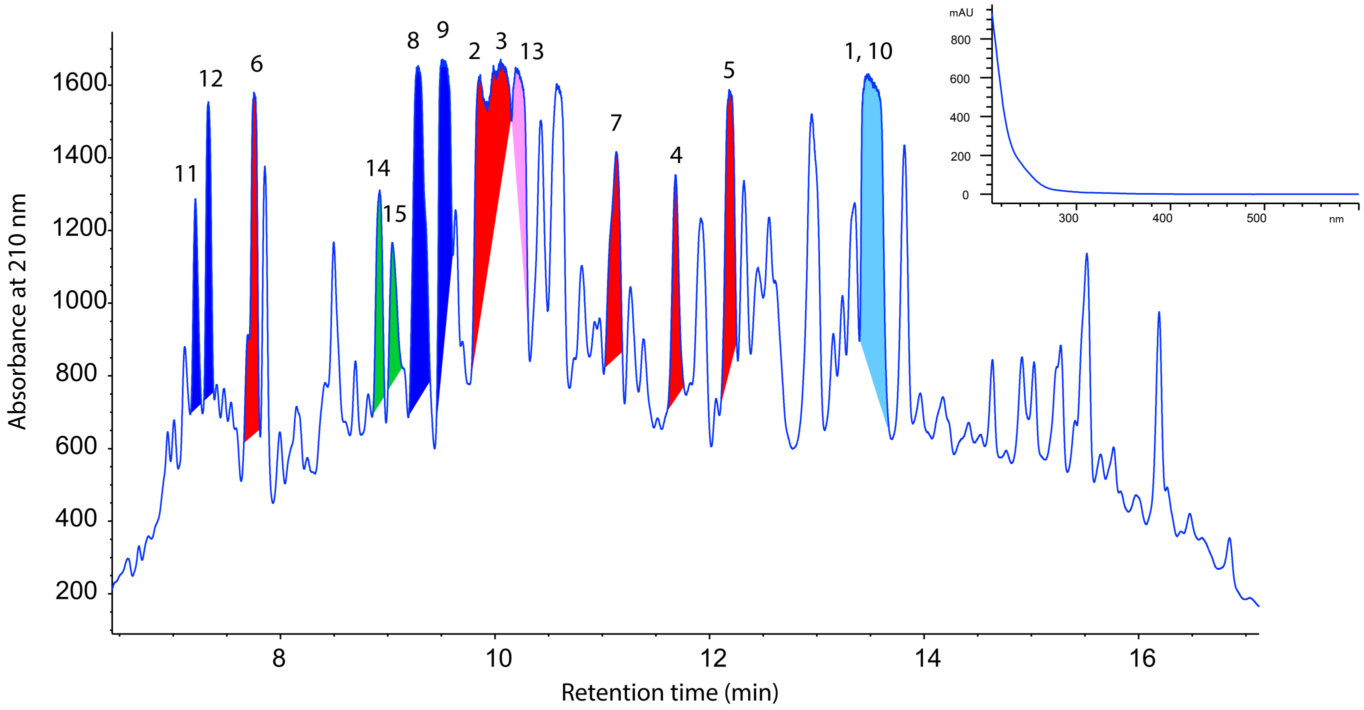

Figure S2. HPLC-DAD (210 nm) chromatogram (C8 column) of DCM partition of the crude extract CMB-03404 {inset: UV spectra for compounds **1-15**}

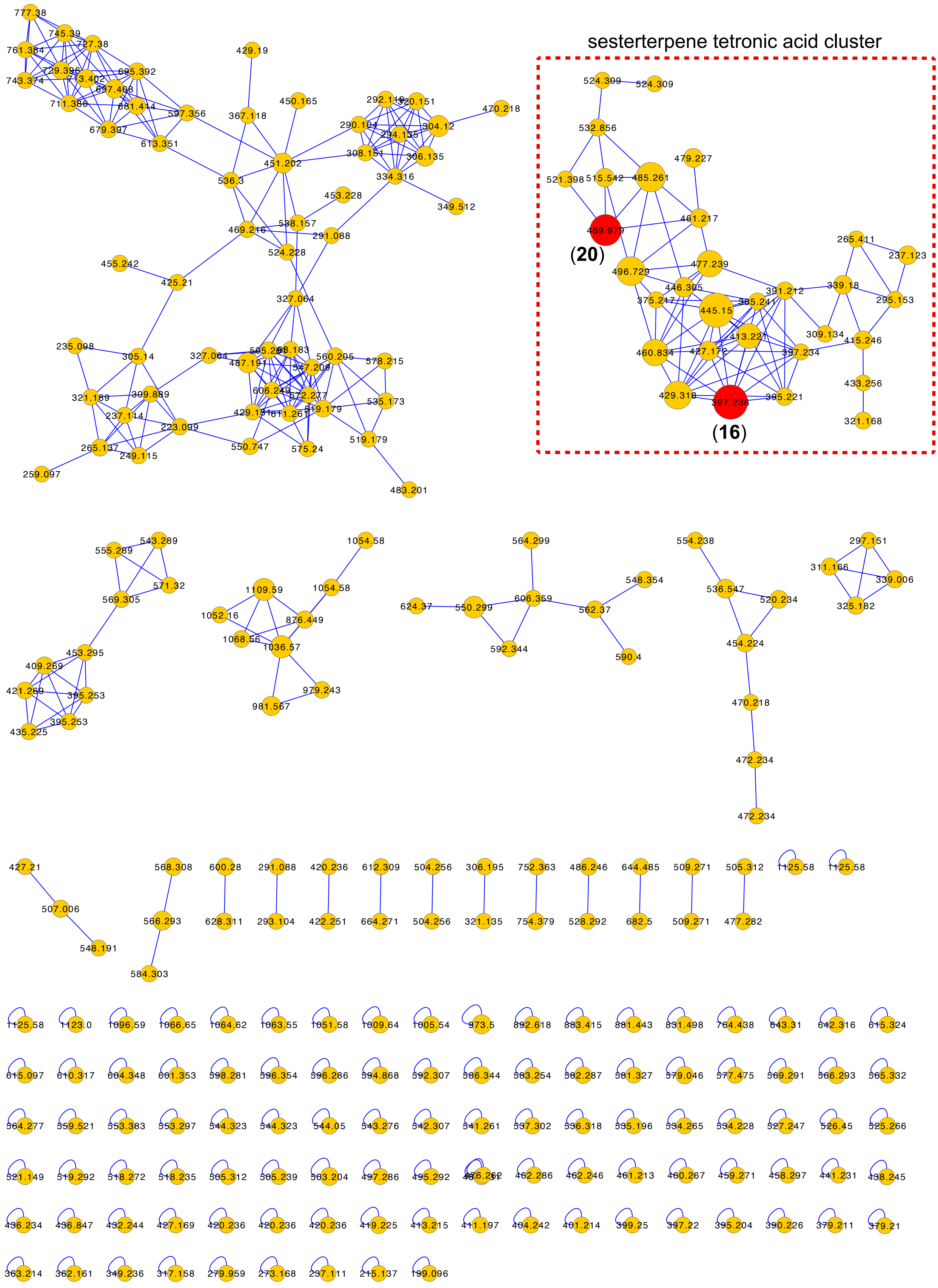

Figure S3. Molecular networking for five *Ircinia sp.* (CMB-01064, CMB-03363, CMB-01058, CMB-01693, CMB-02014) and two authentic standards (7*E*,12*E*,20*Z*,18*S*)-variabilin (**16**), ircinialactam A (**20**) (yellow- nodes from all five *Ircinia sp.* and red- authentic standards)

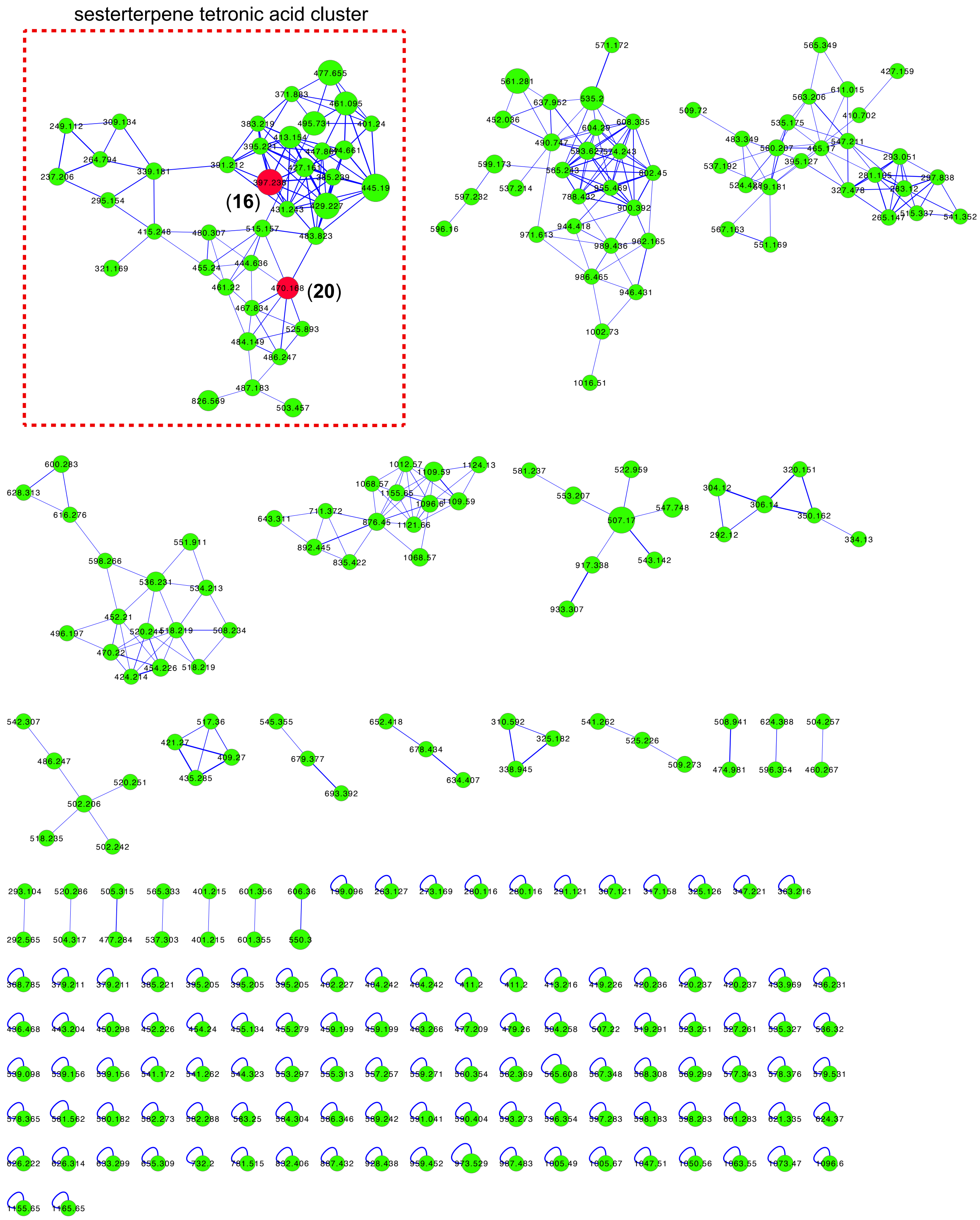

Figure S4. Molecular networking for five *Psammocinia sp.* (CMB-03231, CMB-01018, CMB-03344, CMB-01757, CMB-02026) and two authentic standards (7*E*,12*E*,20*Z*,18*S*)-variabilin (**16**), ircinialactam A (**20**) (green- nodes from all five *Psammocinia sp.* and red- authentic standards)

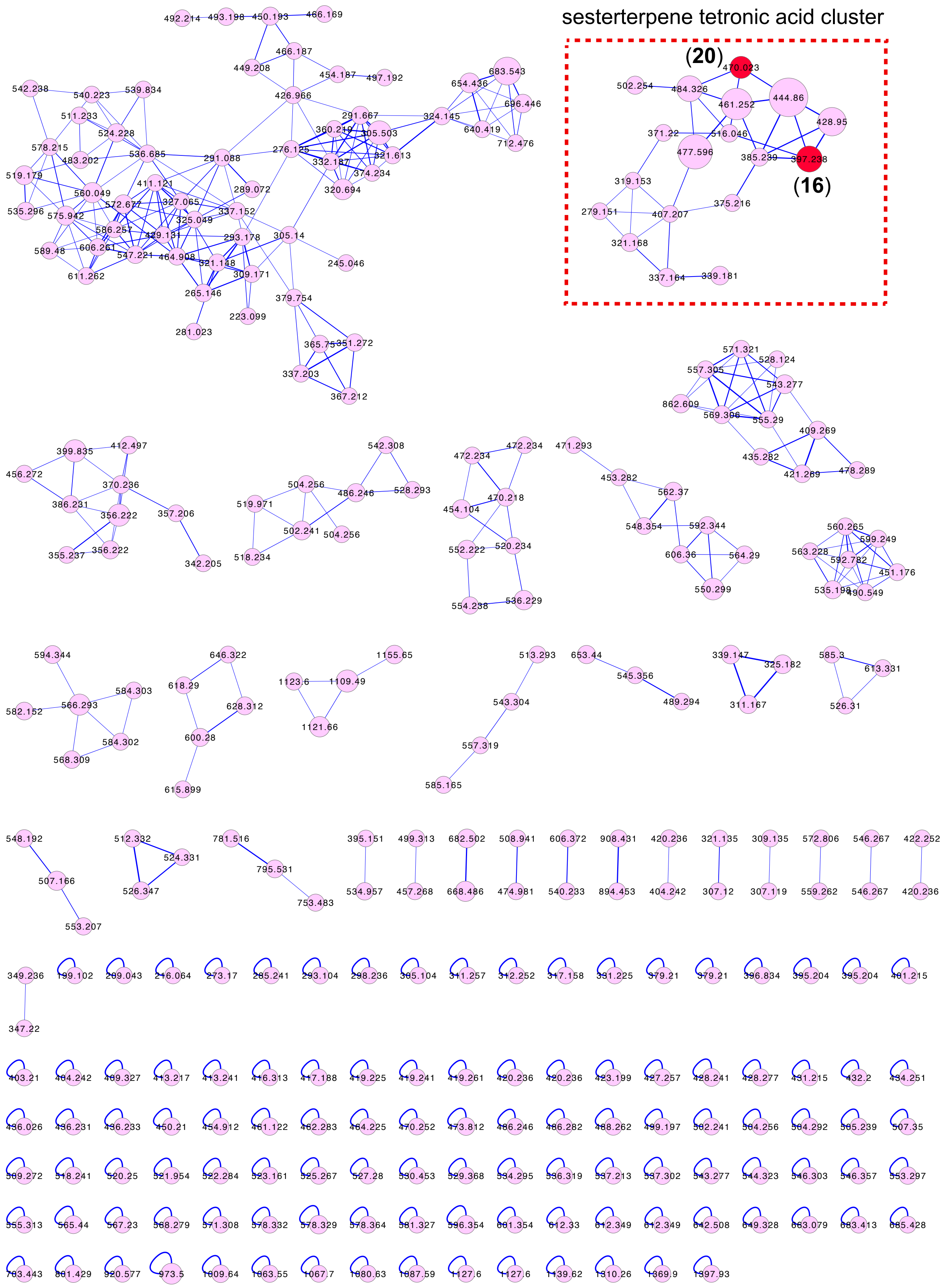

Figure S5. Molecular networking for five *Sarcotragus sp.* (CMB-01788, CMB-01848, CMB-02707, CMB-002717, CMB-03390) and two authentic standards (7*E*,12*E*,20*Z*,18*S*)-variabilin (**16**), ircinialactam A (**20**) (pink- nodes from all five *Sarcotragus sp.* and red- authentic standards)

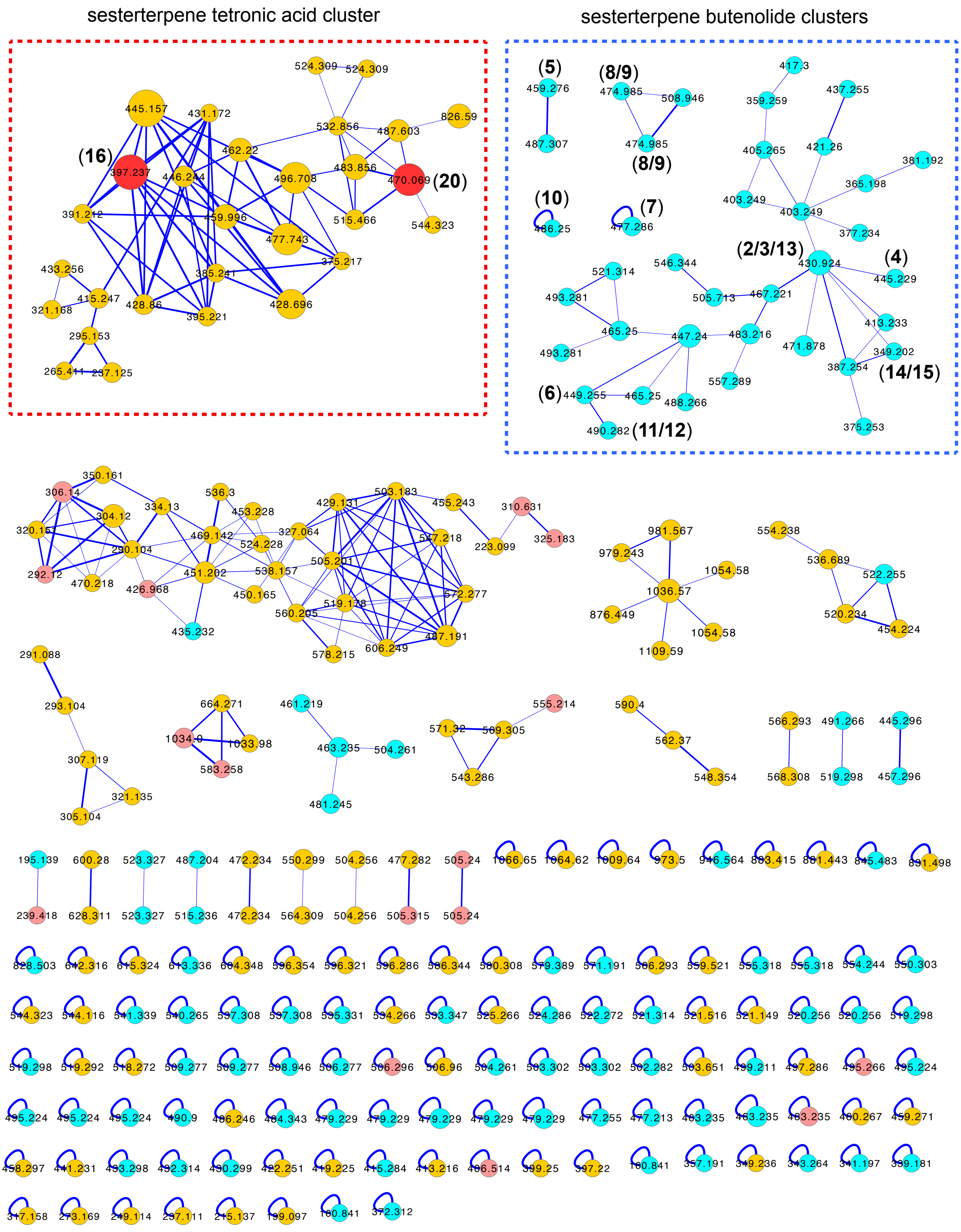

Figure S6. Molecular networking for two *Ircinia sp.* (CMB-01064, CMB-03363), *Cacospongia sp.* (CMB-03404) and two authentic standards (7*E*,12*E*,20*Z*,18*S*)-variabilin (**16**), ircinialactam A (**20**) (yellow- nodes from *Ircinia sp.*, red- authentic standards and pink- commonly present)

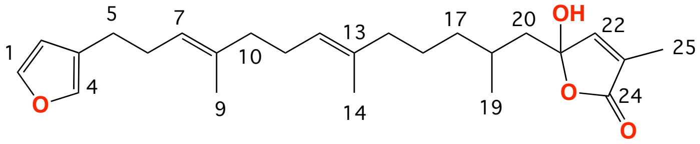

Table S1. NMR (600 MHz, $CDCl_3$) data for cacolide A (**1**)

no.	δ_C	δ_H, mult. (*J* in Hz)	COSY	HMBC	ROESY
1	142.7	7.33, br s	2, 4	2, 3, 4	
2	111.3	6.28, br s	1, 4, 5	1, 3, 4, 5	5
3	125.2				
4	139.0	7.21, br s	2, 5	1, 2, 3	
5	25.2	2.45, t (7.7)	2, 4, 6	2, 4, 6, 7	7
6	28.6	2.24, dt 7.3, 7.7)	5, 7	5, 7, 8	
7	123.9	5.16 (br t, 7.3)	6, 9, 10	5, 6, 9, 10	5, 10
8	135.9				
9	16.2	1.59, s	7, 10	7, 8, 10, 11	
10	39.9	2.01, t (7.4)	7, 9, 11	7, 8, 9, 11	7, 12
11	26.7	2.07, dt (7.4, 7.4)	10, 12, 14	10, 12, 13	
12	124.4	5.04, br t (7.4)	11, 14, 15	11, 14, 15	10, 15
13	135.1				
14	16.0	1.57, s	11, 12, 15	12, 13, 15	
15	39.9	1.93[a]	12, 16	12, 13, 14, 16, 17	12
16	25.2	1.36, m	15, 17	13, 15, 17, 18	
17	37.6	a 1.30, m	15, 16, 17b	15, 16, 18, 19, 20	
		b 1.16, m	17a, 18		
18	28.7	1.64, m	17b, 19, 20		22
19	21.1	0.96, br d (6.7)	18	17, 18, 20	
20	44.9	a 1.94[a]	20b	21	
		b 1.74, m	20a		
21	106.2				
22	147.6	6.82, br s	25	21, 23, 24	18
23	132.1				
24	171.9				
25	10.7	1.93[a], s	22	22, 23, 24	

[a] Overlapping signals

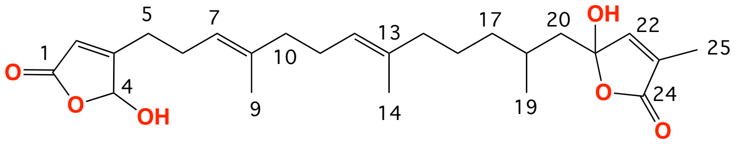

Table S2. Table 2. NMR (600 MHz, $CDCl_3$) data for cacolide B (**2**)

no.	δ_C	δ_H, mult. (*J* in Hz)	COSY	HMBC	ROESY
1	170.0				
2	117.6	5.83, br s	4, 5	1, 3, 4, 5	5
3	171.1				
4	99.5	6.01, br s	2	2, 3	
5	28.0	a. 2.52, m	2, 4, 5b, 6	2, 4, 6, 7	2, 7
		b. 2.39, m	2, 4, 5a, 6		
6	25.3	2.30, dt (7.2, 7.2)	5, 7	5, 7, 8	
7	122.6	5.09, br t (7.2)	6, 9, 10	5, 6, 9, 10	5, 10
8	136.9				
9	16.1[a]	1.61 (s)	7, 10	7, 8, 10, 11	
10	39.5	2.02, t (7.0)	7, 11	7, 8, 9, 11	7, 12
11	25.9	2.09, dt (7.0, 7.0)	10, 12, 14	8, 10, 12, 13	
12	123.9	5.02, br t (7.0)	11, 14, 15	14, 11, 15	10, 15
13	135.4				
14	16.1[a]	1.56 (s)	12, 15	12, 13, 15	
15	39.8	1.91, m	12, 16	12, 13, 14, 16, 17	12
16	25.1	1.34, m	15, 17	13, 15, 17, 18	
17	37.5	a 1.29, m	15, 16, 17b	15, 16, 18, 19, 20	
		b 1.14, m	17a, 18		
18	28.6	1.64, m	19, 20		
19	21.1	0.94, br d (6.5)	18	17, 18, 20	
20	44.7	a 1.95, m	20b	21	
		b 1.71, m	20a		
21	107.0				
22	148.0	6.85 (br s)	25	21, 23, 24	
23	131.9				
24	172.6				
25	10.6	1.90 (s)	22	22, 23, 24	

[a]Signals are interchangeable

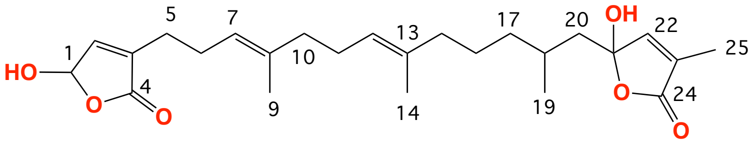

Table S3. NMR (600 MHz, $CDCl_3$) data for cacolide C (**3**)

no.	δ_C	δ_H, mult. (*J* in Hz)	COSY	HMBC	ROESY
1	97.2	6.09, br s	2	3, 4	
2	144.1	6.84[b], br s	5, 1	1, 3, 4, 5	5
3	137.8				
4	172.5				
5	25.4	2.32, t (7.3)	2, 6	2, 4, 6, 7	2, 7
6	25.6	2.26, dt (7.2, 7.3)	5, 7	5, 7, 8	
7	122.9	5.09, br t, (7.2)	6, 9, 10	5, 6, 9, 10	5, 10
8	136.8				
9	16.1[a]	1.59, s	7, 10	7, 8, 10, 11	
10	39.5	2.01, t (7.0)	7, 9, 11	7, 8, 9, 11	7, 12
11	26.1	2.08, dt (7.0, 7.3)	10, 12, 14	8, 10, 12, 13	
12	124.2	5.04, br t (7.3)	11, 14, 15	11, 14, 15	10, 15
13	135.3				
14	16.1[a]	1.56, s	12, 15	12, 13, 15	
15	39.8	1.92, m	12, 16	12, 13, 14, 17	12
16	25.1	1.35, m	15, 17	13, 15, 17, 18	
17	37.5	a 1.30, m	15, 16, 17b	15, 16, 18, 19	
		b 1.14, m	17a, 18		
18	28.6	1.63, m	19		22
19	21.2	0.94, br d (6.3)	18	17, 18, 20	
20	44.7	a 1.93, m	20b	21	
		b 1.71, m	20a		
21	107.0				
22	148.0	6.84[b], br s	25	21, 23, 24	18
23	131.8				
24	172.6				
25	10.6	1.90, s	22	22, 23, 24	

[a]Signals are interchangeable
[b]Overlapping signals

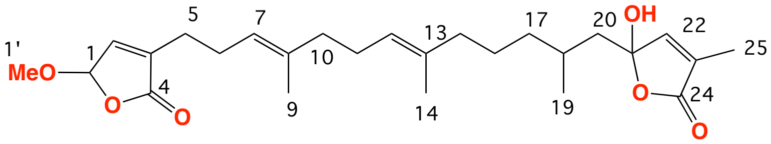

Table S4. NMR (600 MHz, $CDCl_3$) data for cacolide D (**4**)

no.	δ_C	δ_H, mult. (*J* in Hz)	COSY	HMBC	ROESY
1	102.6	5.74, br s	2	OMe, 3, 4	OMe
2	142.2	6.78, br s	1, 5	1, 3, 4, 5	5
3	138.6				
4	171.9				
5	25.5	2.34, t (7.3)	2, 6	2, 3, 4, 6, 7	2, 7
6	25.7	2.26, dt (7.3, 7.2)	5, 7	5, 7, 8	
7	122.6	5.10, br t (7.2)	6, 9	5, 9, 10	5, 10
8	136.8				
9	16.3	1.61, s	7, 10	7, 8, 10	
10	39.7	2.01, t (7.0)	9, 11	7, 8, 9, 11, 12	7
11	26.5	2.08, dt (7.0, 7.3)	10, 12	10, 12, 13	
12	124.2	5.07, br t (7.3)	11, 14	11, 14, 15	15
13	135.2				
14	16.1	1.57, s	12	12, 13, 15	
15	39.9	1.94[a], m	16	12, 13, 14, 16, 17	12
16	25.2	1.35, m	15, 17	13, 15, 17	
17	37.5	a 1.30, m	16, 17b	16	
		b 1.17, m	17a, 18		
18	29.0	1.65, m	19		
19	21.3	0.96, s	18	17, 18, 20	
20	44.9	a 1.94[a], m	20b		
		b 1.73, m	20a		
21	105.8				
22	147.6	6.84, br s	25	21, 24	
23	135.2				
24	171.2				
25	10.8	1.94[a], s	22	22, 23, 24	
1-OMe	57.3	3.56, s		1	1

[a]Overlapping signals

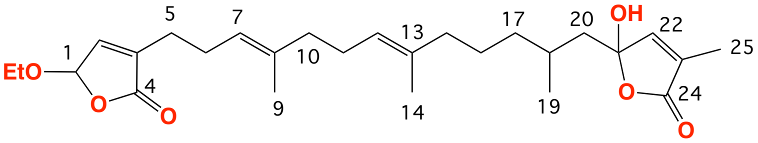

Table S5. NMR (600 MHz, $CDCl_3$) data for cacolide E (**5**)

no.	δ_C	δ_H, mult. (*J* in Hz)	COSY	HMBC	ROESY
1	101.6	5.80, br s	2	$O\underline{C}H_2CH_3$, 2, 3, 4	$O\underline{C}H_2CH_3$
2	142.5	6.78, d (1.4)	1, 5	1, 3, 4, 5	
3	138.3				
4	172.0				
5	25.5	2.33, t (7.3)	2, 6	2, 3, 4, 6, 7	7
6	25.7	2.26, dt (7.2, 7.3)	5, 7	5, 7, 8	
7	122.7	5.10, br t (7.2)	6, 9, 10	6, 9, 10	5, 10
8	136.9				
9	16.0	1.59, s	7, 10	7, 8, 10, 11	
10	39.7	1.99, t (7.0)	7, 9, 11	7, 8, 9, 11, 12	7
11	26.5	2.06, dt (7.3, 7.0)	10, 12, 14	8, 10, 12, 13	
12	124.2	5.06, br t (7.3)	11, 14, 15	11, 14, 15	15
13	135.3				
14	16.2	1.57, s	11, 12, 15	12, 13, 15	
15	39.8	1.93[a], m	12, 16	12, 13, 14, 16, 17	12
16	25.2	1.36, m	15, 17	13, 15, 17, 18	
17	37.3	a 1.27[b], m	17b	15, 16, 18, 19, 20	
		b 1.15, m	17a, 18		
18	28.7	1.67, m	17b, 19, 20		
19	21.2	0.96, s	18	17, 18, 20	
20	44.8	a. 1.93[a]	20b	21	
		b. 1.73, m	20a		
21	106.1				
22	147.6	6.83, br s	25	21, 24, 23	18
23	132.0				
24	172.0				
25	10.6	1.93[a], s	22	22, 23, 24	
$O\underline{CH_2}CH_3$	66.0	a 3.91, m	$O\underline{H}_2C\underline{H}_3$	1, $OCH_2\underline{C}H_3$	1
		b 3.73, m	$OC\underline{H}_2C\underline{H}_3$		
$OCH_2\underline{CH_3}$	15.2	1.27[b], t (7.0)	$OC\underline{H}_2CH_3$	$O\underline{C}H_2CH_3$	

[a, b] Overlapping signals

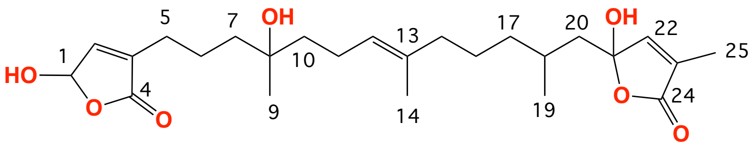

Table S6. NMR (600 MHz, $CDCl_3$) data for cacolide F (**6**)

no.	δ_C	δ_H, mult. (*J* in Hz)	COSY	HMBC
1	96.9	6.05, br s	2	
2	143.8	6.89, br s	1	1, 3, 4
3	137.8			
4	172.3			
5	25.5	2.32, m	6	
6	21.6	1.64[a]	5, 7	5, 7, 8
7	41.5[d]	a. 1.55[b]	6	5, 8
		b. 1.50[c]		
8	73.5			
9	26.4	1.19, s		8, 10
10	41.5[d]	a 1.55[b]	11	12
		b 1.50[c]		
11	22.9	2.07, m	10, 12	
12	125.1	5.27 (m)	11, 14	
13	135.2			
14	16.1	1.64[a], br s	12	
15	39.3	1.97, m	16	
16	24.6	1.41, m	15, 17	
17	36.6	a 1.31, m	17b	
		b 1.11, m	17a	
18	28.6	1.70, m	19	
19	21.1	0.94, br d (7.0)	18	17, 18, 20
20	45.4*	#		
21	107.5			
22	147.6	6.85, br s	25	
23	131.7			
24	172.0			
25	10.7	1.91, s	22	22, 23, 24

[a-c] Overlapping signals
[d]Signals are interchangeable
*Detected from HMBC
#Not detected

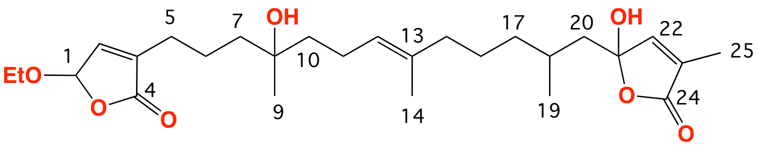

Table S7. NMR (600 MHz, $CDCl_3$) data for cacolide G (**7**)

no.	δ_C	δ_H, mult. (J in Hz)	COSY	HMBC
1	101.8	5.82, br s	2	$O\underline{C}H_2CH_3$, 3, 4
2	142.6	6.81, br s	1, 5	4, 1, 3
3	138.5			
4	171.9			2, 1
5	25.9	2.30, (7.3)	2, 6	6, 7, 3, 2, 4
6	21.9	1.64[a]	5, 7	5
7	41.6[d]	a. 1.64[a]	6	8, 5, 10, 6
		b. 1.51[b]		
8	73.1			
9	26.9	1.18, s		10, 8, 7
10	41.6[d]	a 1.64[a]	11	9, 7, 8, 11
		b 1.51[b]		
11	22.8	2.05, m	10, 12	13, 10, 12
12	125.0	5.15, m	11, 14	
13	135.9			
14	16.0	1.60, br s	12	15, 12, 13
15	39.5	1.96, m	16	13, 16, 12
16	25.1	1.36, m	15	
17	37.5	a 1.33, m	17b	
		b 1.13, m	17a	
18	28.6	1.66, m	19	
19	21.0	0.96, br d (6.8)	18, 20a	17, 18, 20
20	45.1	a 1.93[c], m	20b, 19	19
		b 1.72, m	20a	
21	106.0			
22	147.5	6.83, br s	25	21, 24
23	132.3			
24	173.0			
25	10.6	1.93[c], s	22	23, 22, 24
$O\underline{CH_2}CH_3$	66.2	a 3.93, m	$OCH_2\underline{CH_3}$	1, $OCH_2\underline{C}H_3$
		b 3.74, m	$OCH_2\underline{CH_3}$	
$OCH_2\underline{CH_3}$	15.2	1.27, t (7)	$OC\underline{H_2}CH_3$	$O\underline{C}H_2CH_3$

[a-c]Overlapping signals
[d]Signals are interchangeable

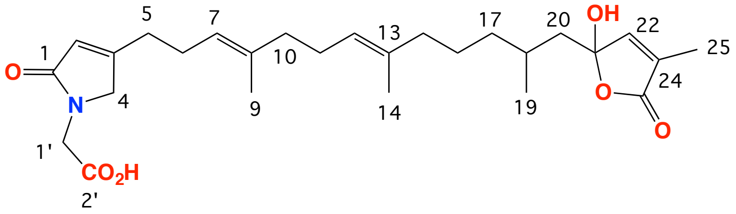

Table S8. NMR (600 MHz, $CDCl_3$) data for cacolide H (**8**)

no.	δ_C	δ_H, mult. (*J* in Hz)	COSY	HMBC	ROESY
1	173.5				
2	121.0	5.91, br s	5, 4	4, 1, 5, 3	5
3	162.3				
4	55.7	4.04, br s	2	2, 1	5, 6
5	30.0	2.43, t (7.5)	2, 6	2, 3, 4, 6, 7	2, 4, 7
6	26.0	2.28, m	5, 7	5, 7, 8, 3	4
7	122.8	5.09, br t (7.0)	6, 9, 10	9, 10, 5, 6	5, 10
8	136.8				
9	16.2	1.60, s	7, 10	10, 8, 7	
10	39.6	2.02, m	9, 7, 11	11, 9, 7, 8	7, 12
11	26.2	2.09, m	10, 12	8, 10, 12, 13	
12	124.1	5.04, br t (7.0)	11, 14, 15	14, 11, 15	10, 15
13	135.3				
14	16.1	1.56, br s	11, 12, 15	12, 13	
15	39.8	1.92, m	12, 14, 16	12, 14, 17, 16, 13	12
16	25.1	1.36, m	15, 17	17	
17	37.4	a. 1.30, m	17b, 16		
		b. 1.14, m	17a, 16, 18		
18	28.6	1.64, m	19, 17b		
19	21.1	0.94, br d (7.0)	18	17, 18, 20	
20	44.8	a 1.95, m	20b		
		b 1.70, m	20a		
21	107.5				
22	148.0	6.82, br s	25		
23	131.7				
24	172.4				
25	10.8	1.90, s	22	22, 23, 24	
1′	43.7	a 4.22, m		2′	
		b 4.18, m			
2′	171.7				

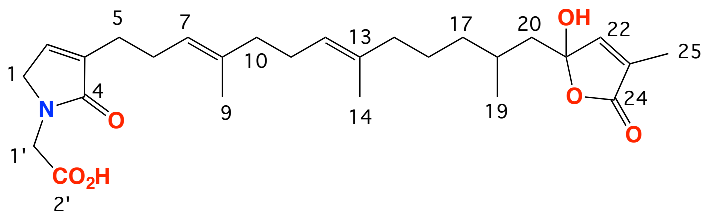

Table S9. NMR (600 MHz, $CDCl_3$) data for cacolide I (**9**)

no.	δ_C	δ_H, mult. (*J* in Hz)	COSY	HMBC	ROESY
1	52.1	4.03, br s	2	2, 3	2
2	136.9	6.77, br s	1, 5	4, 1, 3, 5	1, 5
3	139.2				
4	173.3				
5	25.9	2.30, t (7.2)	2, 6	2, 3, 4, 6, 7	2, 7
6	26.0	2.24, dt (7.3, 7.3)	7, 5, 10	3, 5, 7, 8	
7	123.7	5.11, br t (7.3)	6, 9, 10	9, 10, 5	5, 10
8	135.9				
9	16.1	1.59, s	7, 10	7, 10, 8	
10	39.5	2.01, t (7.0)	6, 7, 9, 11	8, 9, 11, 12	7, 12
11	26.1	2.08, dt (7.0, 7.0)	10, 12, 14	10, 12, 13	
12	124.3	5.04, br t (7.0)	11, 14, 15	14, 11, 15	10, 15
13	135.4				
14	16.0	1.56, s	11, 12, 15	15, 12, 13	
15	39.8	1.92, m	12, 14, 16	12, 16, 13, 14, 17	12
16	25.1	1.36, m	15, 17a	13, 15	
17	37.0	a 1.27, m	17b	15	
		b 1.13, m	17a, 16, 18		
18	29.1	1.62, m	19, 17a		
19	21.1	0.94, br s	18	17, 18, 20	
20	44.8	a 1.95, m	20b		
		b 1.69, m	20a		
21	107.5				
22	148.0	6.83, br s	25	21, 24	
23	131.7				
24	171.9				
25	10.6	1.90, s	22	22, 23, 24	
1′	44.1	a 4.28, m		1, 4, 2′	
		b 4.25, m			
2′	172.6				

[a, b] Overlapping signals

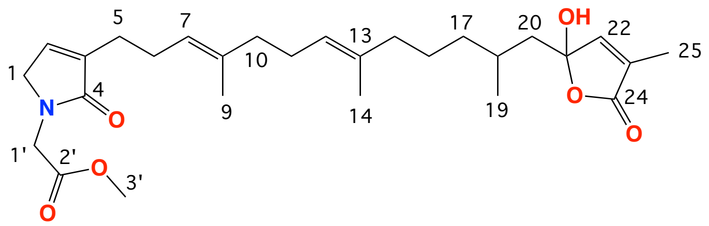

Table S10. NMR (600 MHz, $CDCl_3$) data for cacolide J (**10**)

no.	δ_C	δ_H, mult. (J in Hz)	COSY	HMBC
1	51.7	4.00, br s	2	2, 3, 4
2	136.0	6.74, br s	1, 5	1, 3, 4, 5
3	139.9			
4	173.1			
5	26.3	2.32, t (7.2)	2, 6	2, 3, 4, 6, 7
6	26.1	2.25, dt (7.3, 7.3)	7, 5, 10	3, 5, 7, 8
7	124.1	5.15, br t (7.3)	6, 9, 10	5, 9, 10
8	135.6			
9	16.1	1.60, s	7, 10	7, 8, 10
10	39.6	2.03, t (7.0)	6, 7, 9, 11	8, 9, 11, 12
11	25.9	2.10, dt (7.0, 7.0)	10, 12, 14	10, 12, 13
12	124.2	5.06, br t (7.0)	11, 14, 15	11, 14, 15
13	135.2			
14	16.0	1.56, s	11, 12, 15	12, 13, 15
15	39.9	1.93, m	12, 14, 16	12, 13, 14, 16, 17
16	25.1	1.37, m	15, 17	13, 15, 17, 18
17	37.8	a 1.28, m	17b, 16	15, 18
		b 1.16, m	17a, 16, 18	
18	28.8	1.64, m	19, 17b	
19	21.4	0.95, br s	18	17, 18, 20
20	44.9	a 2.00, m	20b	
		b 1.71, m	20a	
21				
22	148.1	6.83, br s	25	
23	132.0			
24	175.5			
25	10.7	1.91, s	22	22, 23, 24
1′	43.8	a 4.25, m	1′b, 3′	2′
		b 4.21, m	1′a, 3′	
2′	170.3			
3′	52.4	3.74, s	1′	2′

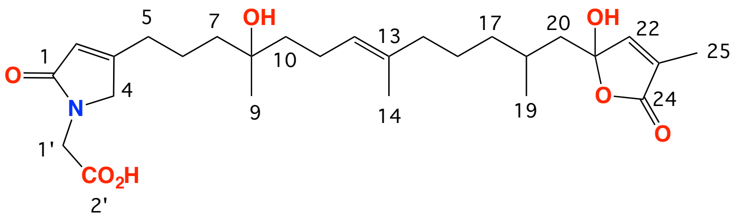

Table S11. NMR (600 MHz, $CDCl_3$) data for cacolide K (**11**)

no.	δ_C	δ_H, mult. (J in Hz)	COSY	HMBC	ROESY
1	173.2				
2	121.5	5.93, br s	4, 5	1, 4	5
3	161.8				
4	55.6	4.04, br s	2	1, 2, 3	5, 6
5	30.3	2.40, t (7.5)	2, 6	2, 3	2, 4
6	22.3	1.66^a, m	7, 5		4
7	41.5^d	a 1.54^b	6	6, 8	
		b 1.52^c			
8	72.7				
9	27.0	1.19, s	10	7, 8, 10	
10	41.5^d	a 1.54^b	9, 11	11, 12, 8	12
		b 1.52^c			
11	23.0	2.07, m	10, 12	12, 13	
12	125.0	5.15, br t (7.0)	11, 14		10, 15
13	134.8				
14	16.1	1.59, br s	12	15, 12, 13	
15	39.5	1.96, m	16	13	12
16	24.7	1.39, m	15		
17	37.1	a 1.32, m	17b		
		b 1.11, m	17a		
18	28.6	1.66^a, m	19		
19	21.3	0.94, br d (7.0)	18	17, 18, 20	
20	44.9*	#			
21	#				
22	147.5	6.81, br s	25		
23	132.0				
24	172.0				
25	10.6	1.91, s	22	22, 23, 24	
1′	44.1	4.21, br s		2′	
2′	171.6				

a-c Overlapping signals
d Signals are interchangeable
* Detected from HMBC
# Not detected

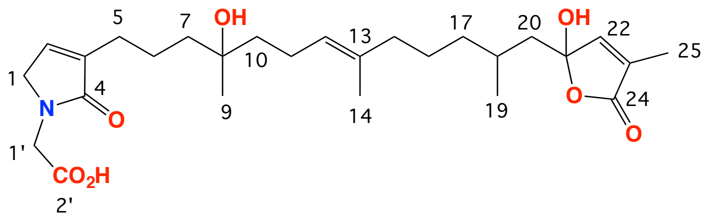

Table S12. NMR (600 MHz, $CDCl_3$) data for cacolide L (**12**)

Pos.	δ_C	δ_H, mult. (J in Hz)	COSY	HMBC	ROESY
1	52.1	4.02, br s	2	2, 3	2
2	136.9	6.78, br s	5, 1	4, 1	1, 5
3	139.4				
4	173.0				
5	26.5	2.32, t (7.5)	2, 6	2, 3, 4, 6	2
6	22.3	1.61, m	5, 7		
7	41.5[e]	a. 1.52[a]	6	8, 9, 10	
		b. 1.50[b]			
8	73.4				
9	26.8	1.18 (s)		8, 10	
10	41.5[e]	a 1.52[a]	11	11, 12	12
		b 1.50[b]			
11	22.8	2.05, m	10, 12	10, 12, 13	
12	125.1	5.14, br t (7.0)	11, 14	14, 11, 15	10, 15
13	135.4				
14	15.9	1.59, br s	12	15, 12, 13	
15	39.4	1.95[d]	16	16, 13	12
16	24.5	1.39, m	15		
17	37.0	a 1.33, m	17b		
		b 1.10, m	17a		
18	28.5	1.65, m	19		
19	21.1	0.94, br d (7.0)	18	17, 18, 20	
20	45.2	a 1.95[d]	20b		
		b 1.73, m	20a		
21	107.8				
22	148.1	6.83, s	25		
23	131.7				
24	172.4				
25	10.8	1.90, s	22	22, 23, 24	
1′	44.4	4.23, s		4, 2′	
2′	171.3				

[a-d]Overlapping signals
[e]Signals are interchangeable

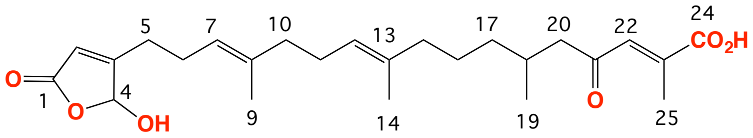

Table S13. NMR (600 MHz, $CDCl_3$) data for cacolic acid A (**13**)

no.	δ_C	δ_H, mult. (J in Hz)	COSY	HMBC	ROESY
1	171.4				
2	118.0	5.85, br s	4, 5	1, 4	6
3	169.5				
4	99.3	5.99, br s	2	1	
5	27.9	2.46, m	2, 6	3	7
6	25.3	2.32, m	7, 5	7, 8, 3	2
7	122.6	5.09, br t (7.3)	6, 9, 10	9, 10, 6	5, 10
8	137.3				
9	16.2	1.59, br s	7	10, 8, 7	
10	39.4	2.03[a]	7, 11	7, 8, 9, 11, 12	7, 12
11	26.1	2.09 (m)	10, 12	8, 12, 13	
12	124.1	5.03, br t (7.0)	11, 14, 15	14, 11, 15	10, 15
13	135.5				
14	16.1	1.57, br s	12	15, 12, 13	
15	39.7	1.94, m	12, 16	12, 13, 14, 16, 17	12
16	25.2	1.37, m	15, 17	17	
17	36.4	a 1.25, m	17b, 16, 18		
		b 1.15, m	17a, 18, 16		
18	29.7	2.03[a]	17, 19, 20	19, 20	
19	20.1	0.91, br d (6.7)	18	17, 18, 20	
20	52.6	a 2.53, m	20b, 18	18, 19, 21	22
		b 2.35, m	20a, 18		
21	202.9				
22	133.8	7.11, br s	25	21, 24, 25	20
23	140.5				
24	170.7				
25	14.4	2.20, s	22	22, 23, 24	

[a] Overlapping signals

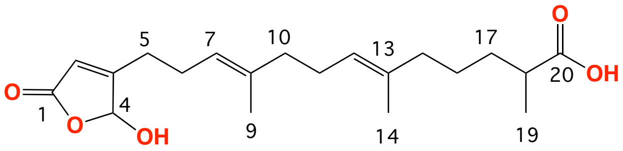

Table S14. NMR (600 MHz, $CDCl_3$) data for cacolic acid B (**14**)

no.	δ_C	δ_H, mult. (J in Hz)	COSY	HMBC	ROESY
1	171.4				
2	118.0	5.88, br s	4, 5	5, 4, 1, 3	5, 6, 7
3	169.5				
4	99.3	6.01, br	2	2, 1	5, 6
5	27.9	2.47[a]	2, 6		2, 4, 7
6	25.3	2.33, m	5, 7	5, 7, 8, 3	2, 4, 7
7	122.8	5.10, br t (7.3)	6, 9, 10	9, 10, 5	2, 5, 6, 10
8	137.0				
9	16.1	1.64[b], br s	7	10, 8, 7	
10	39.4	2.06, m	7, 11	7, 8, 12	7
11	25.8	2.12, m	10, 12	8, 10, 12, 13	
12	124.1	5.06, br t (7.0)	11, 14	14, 11, 15	15, 16
13	135.2				
14	16.1	1.59, br s	12	15, 12, 13	
15	39.5	1.98, m	16	12, 13, 14, 16, 17	12
16	25.6	1.42[c]	15, 17b	17	12
17	33.4	a 1.64[b]	17b, 18	16, 20	
		b 1.42[c]	17a, 16		
18	39.2	2.47[a]	17a, 19	19, 17, 20, 16	19
19	17.2	1.19, br d (7.2)	18	17, 18, 20	18
20	180.2				

[a-c]Overlapping signals

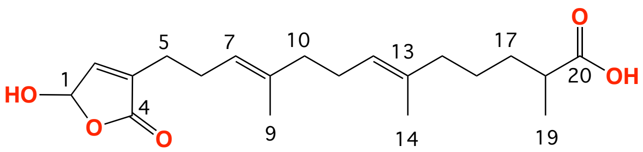

Table S15. NMR (600 MHz, $CDCl_3$) data for cacolic acid C (**15**)

no.	δ_C	δ_H, mult. (*J* in Hz)	COSY	HMBC	ROESY
1	97.2	6.09, br s	2	3, 4	2
2	143.9	6.84, br s	1, 5	1, 3, 4	1, 5, 6
3	138.6				
4	172.3				
5	25.3	2.32, t (7.3)	2, 6	2, 3, 4, 6, 7	2, 7
6	25.6	2.25, dt (7.3, 7.2)	5, 7	5, 7, 8	2, 7
7	123.0	5.09 (br t, 7.2)	6, 9	9, 10, 5	5, 6, 10
8	137.3				
9	16.2	1.64[a], s	7	7, 8, 10	
10	39.6	2.02, br t (7.0)	11	8, 9, 11, 12	7
11	26.1	2.09, dt (7.2, 7.0)	10, 12	10, 12, 13	
12	124.4	5.04, br t (7.2)	11, 14	14, 11, 15	15
13	135.6				
14	16.2	1.57, s	11, 12	15, 12, 13	
15	39.6	1.96, m	16	16, 13, 12, 14, 17	12
16	25.6	1.42[b]	15, 17a	17	
17	33.6	a 1.64[a]	16, 17b, 18	16, 20	
		b 1.42[b]	17a, 18		
18	39.1	2.47, m	17a, 17b, 19	17, 16	19
19	17.3	1.17, br d (7.0)	18	17, 18, 20	18
20	180.4				

[a, b] Overlapping signals

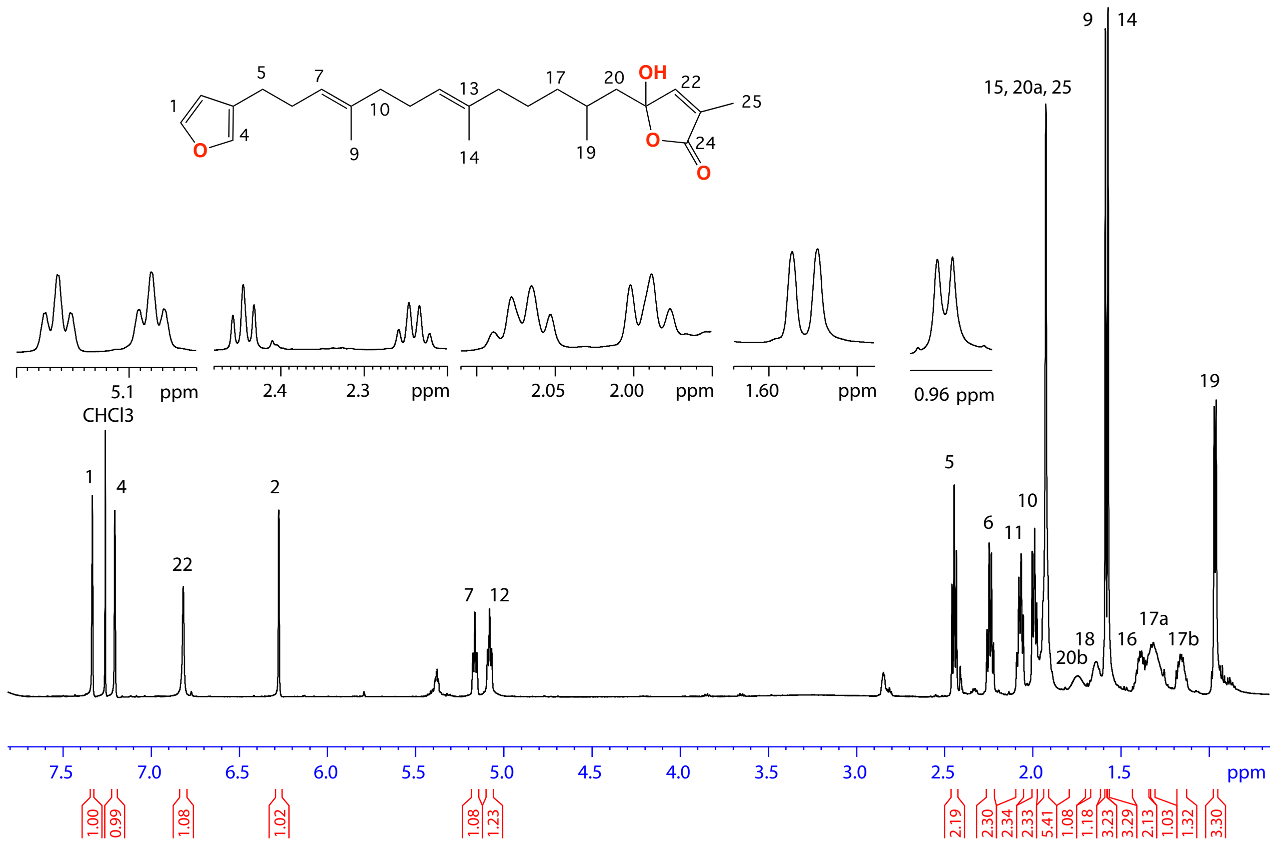

Figure S7. ^{1}H NMR (600 MHz, $CDCl_3$) spectrum of cacolide A (**1**)

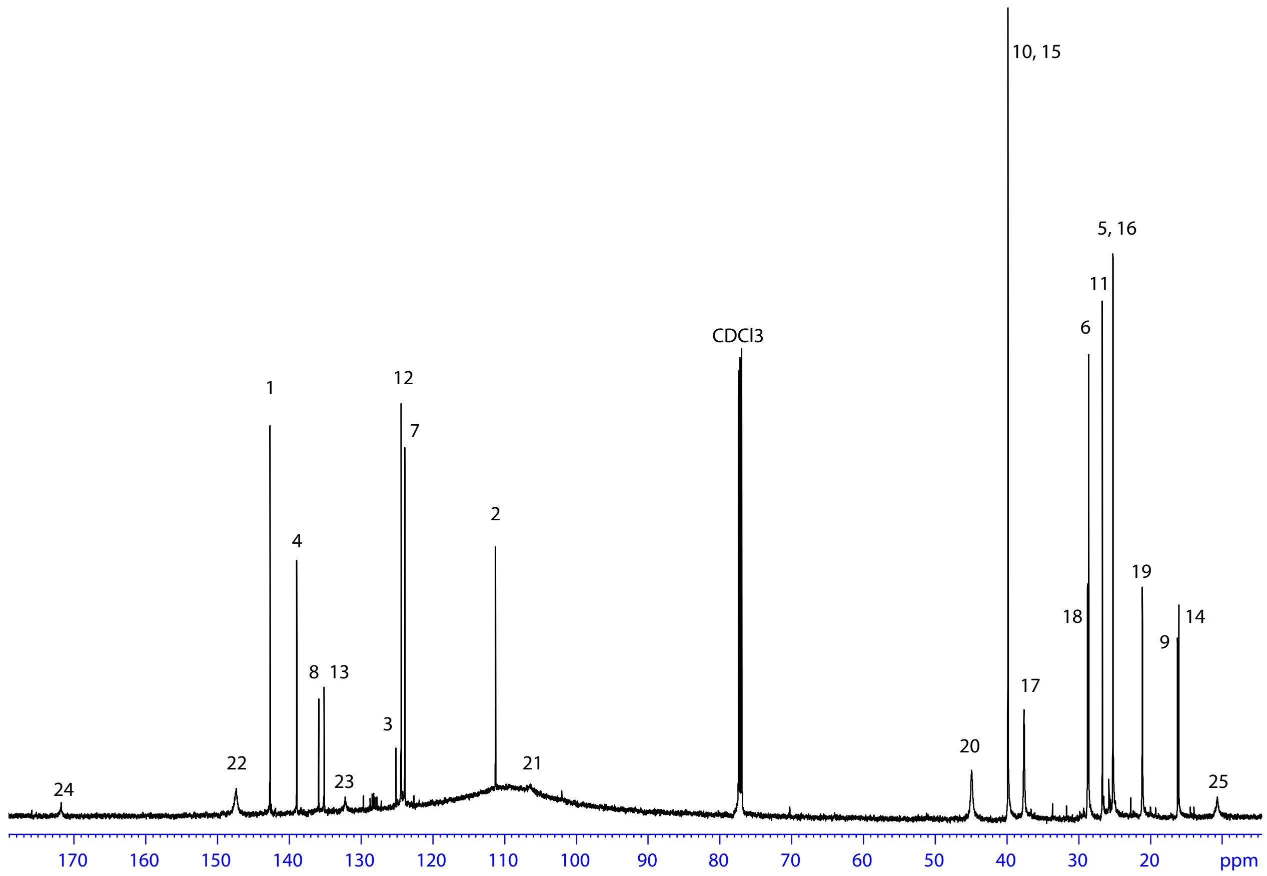

Figure S8. ^{13}C NMR (150 MHz, $CDCl_3$) spectrum of cacolide A (**1**)

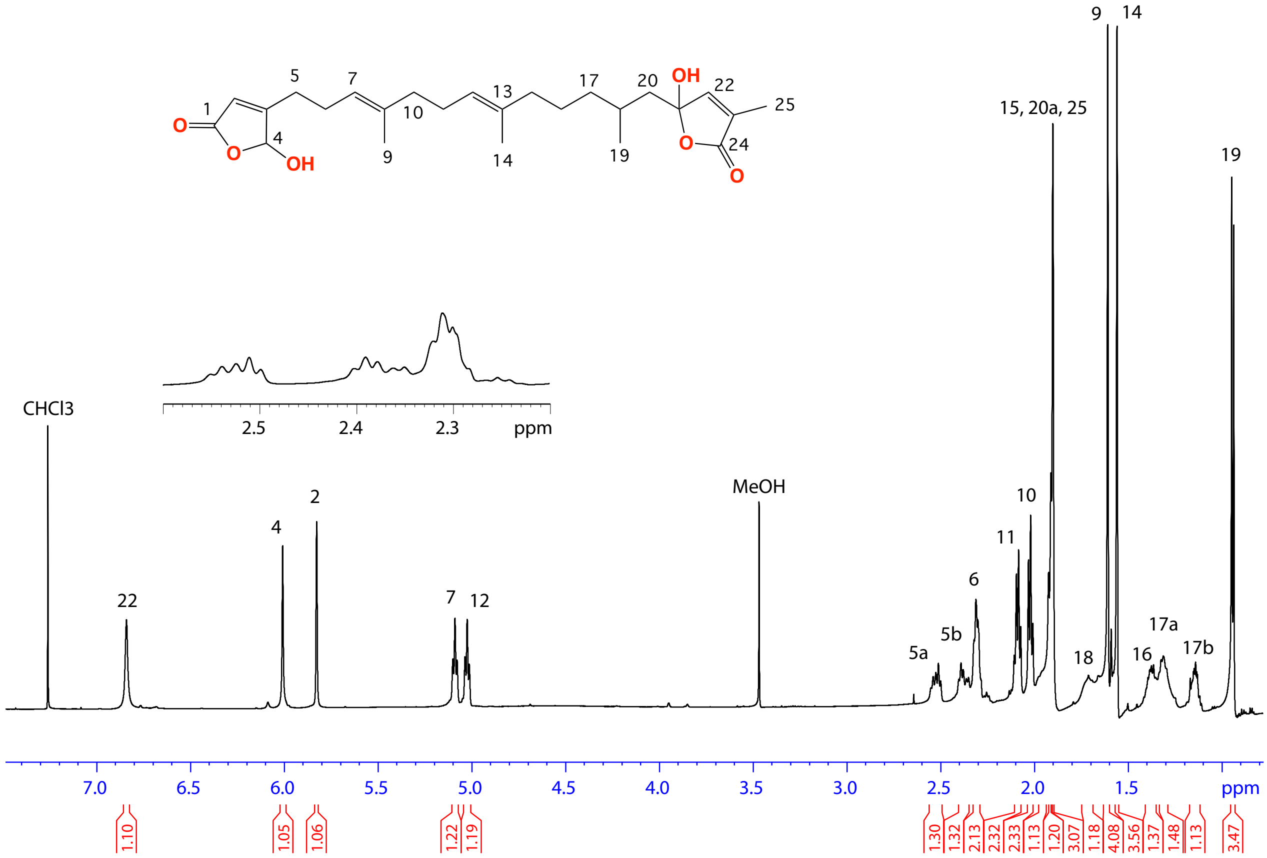

Figure S9. ^{1}H NMR (600 MHz, $CDCl_3$) spectrum of cacolide B (**2**)

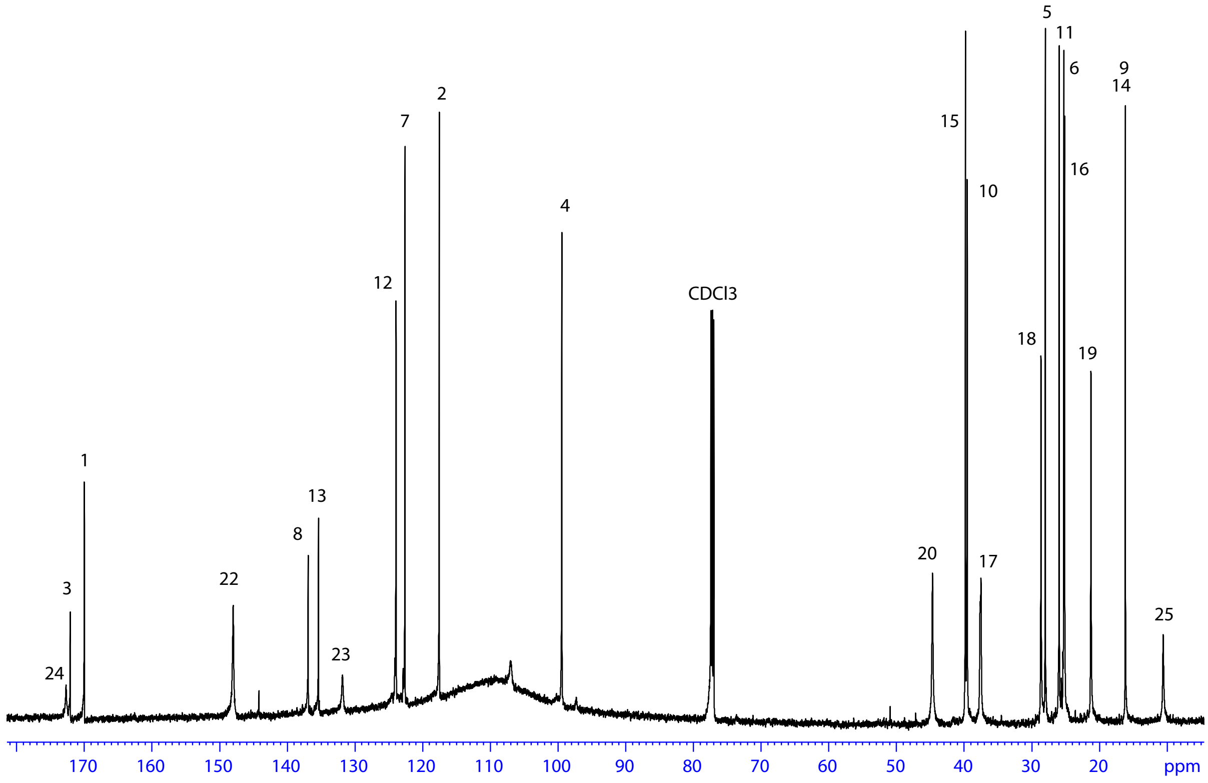

Figure S10. ^{13}C NMR (150 MHz, $CDCl_3$) spectrum of cacolide B (**2**)

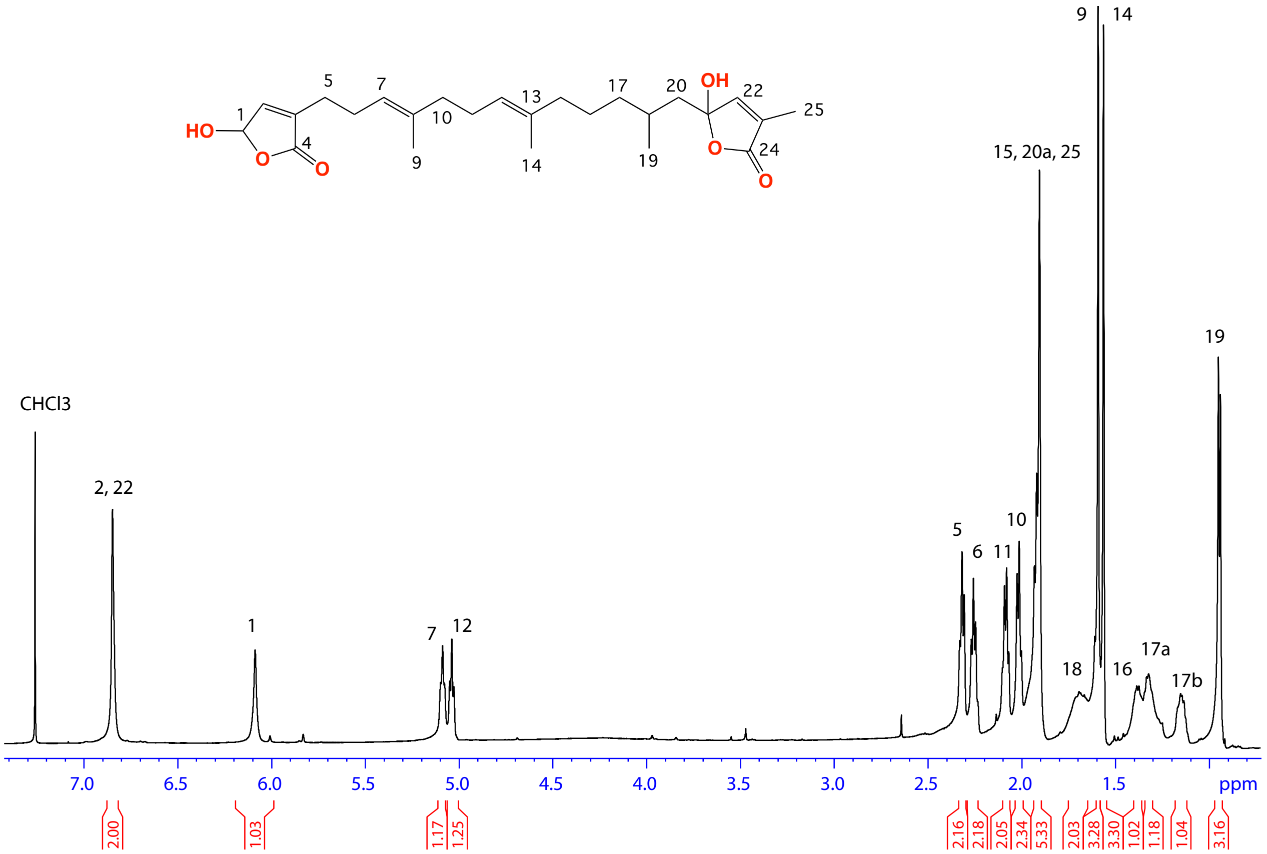

Figure S11. ^{1}H NMR (600 MHz, $CDCl_3$) spectrum of cacolide C (**3**)

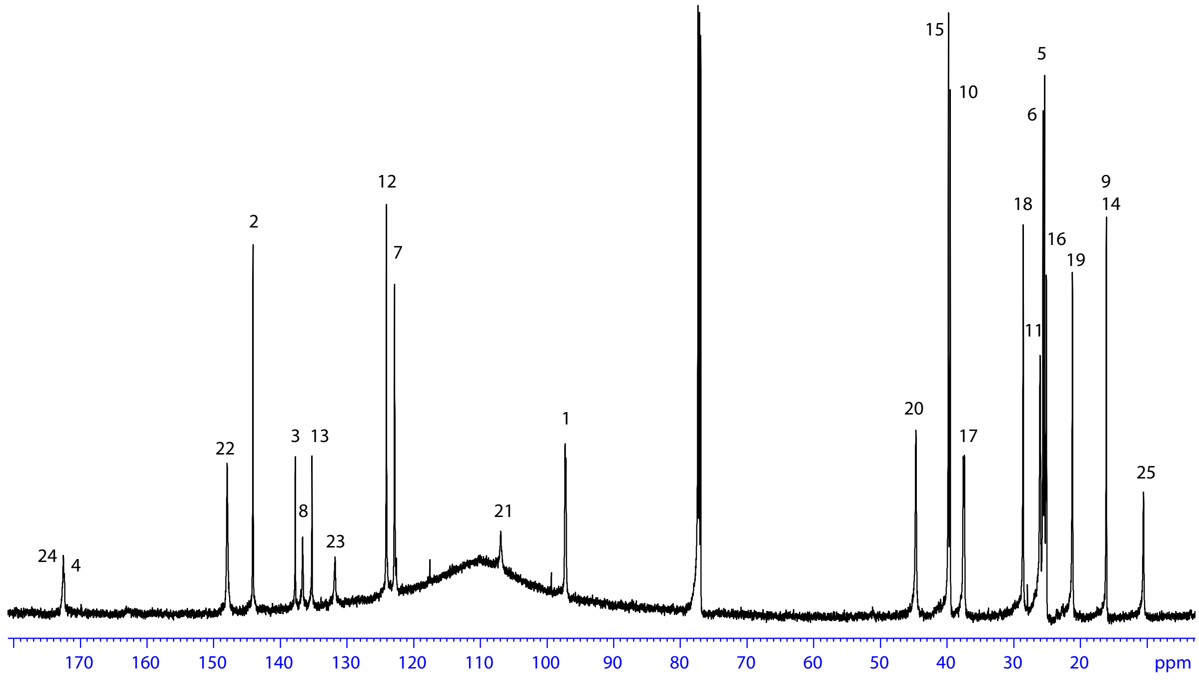

Figure S12. ^{13}C NMR (150 MHz, $CDCl_3$) spectrum of cacolide C (**3**)

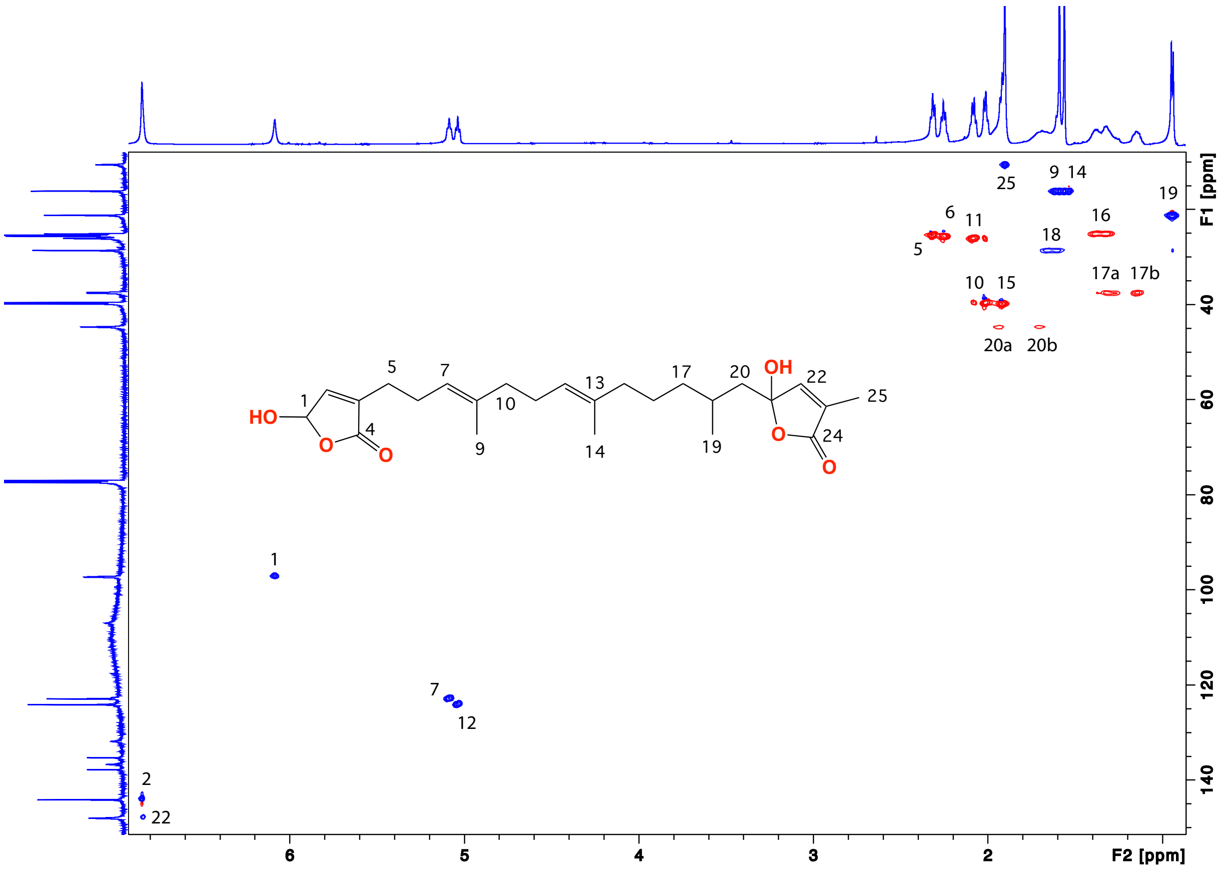

Figure S13. HSQC NMR (600 MHz,$CDCl_3$) spectrum of cacolide C (**3**)

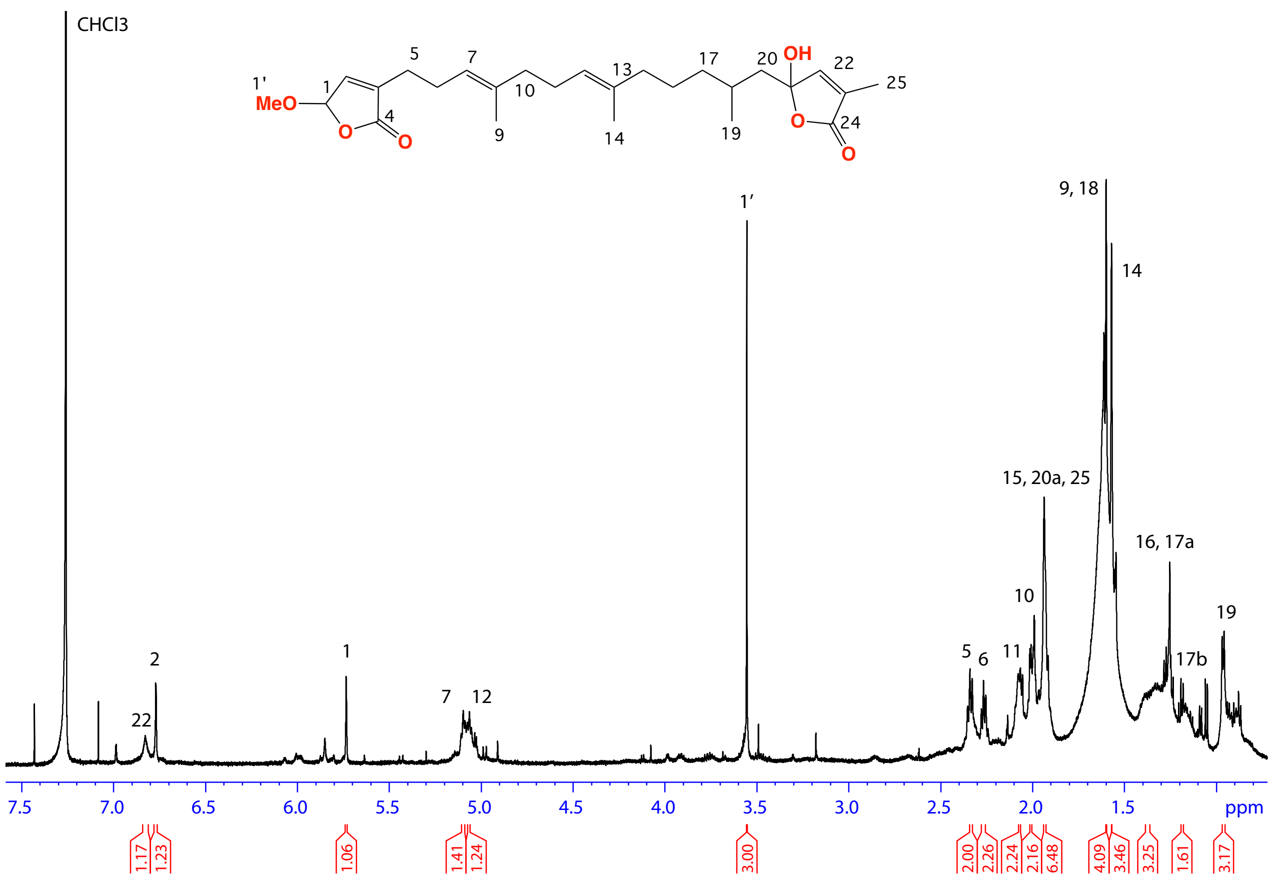

Figure S14. ^{1}H NMR (600 MHz, $CDCl_3$) spectrum of cacolide D (**4**)

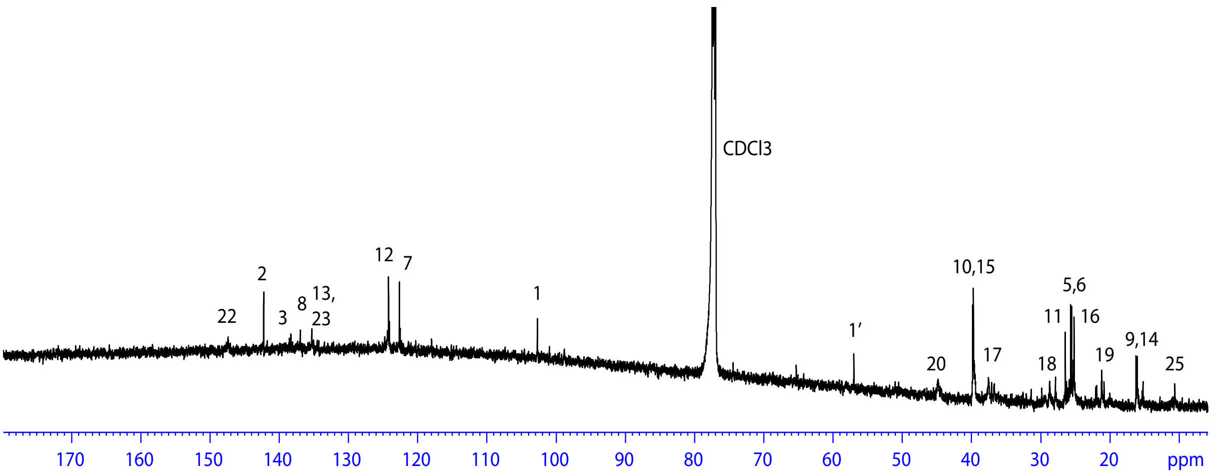

Figure S15. ^{13}C NMR (150 MHz, $CDCl_3$) spectrum of cacolide D (**4**)

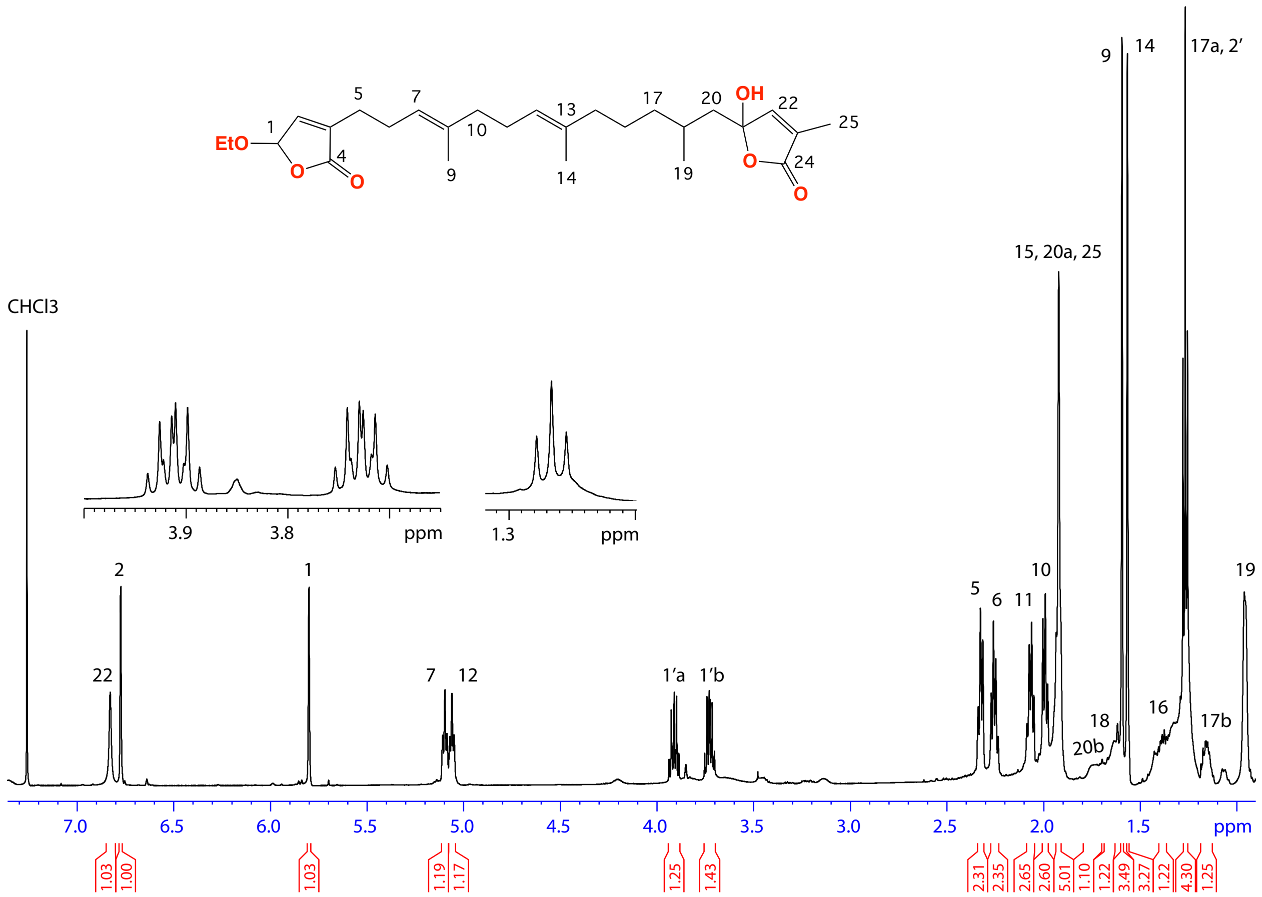

Figure S16. ^{1}H NMR (600 MHz, $CDCl_3$) spectrum of cacolide E (**5**)

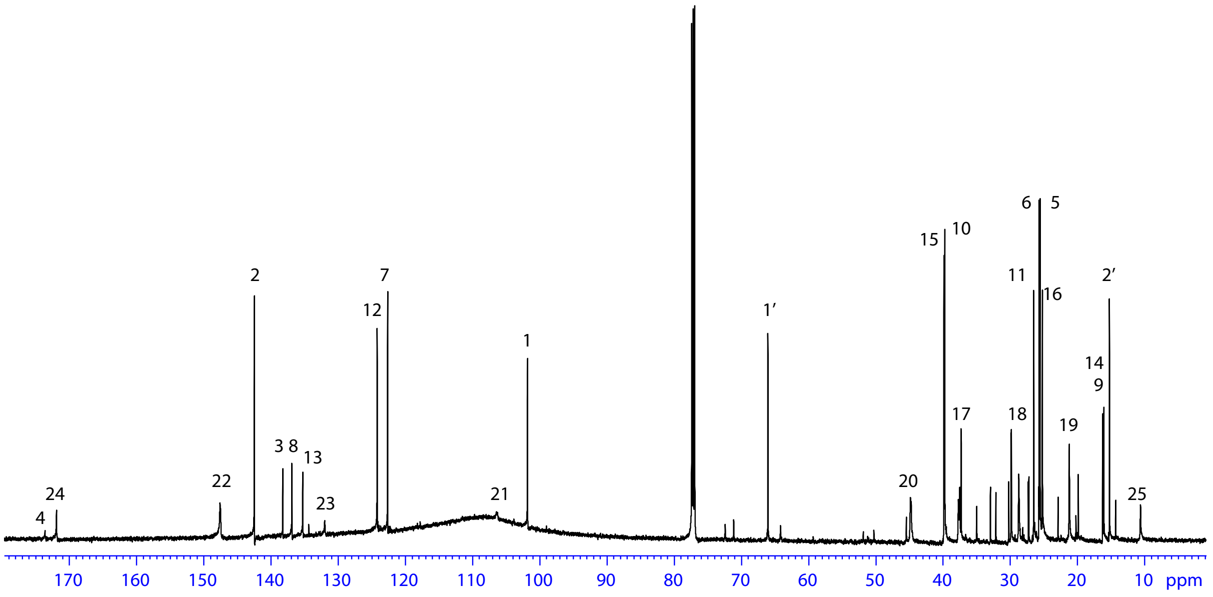

Figure S17. ^{13}C NMR (150 MHz, $CDCl_3$) spectrum of cacolide E (**5**)

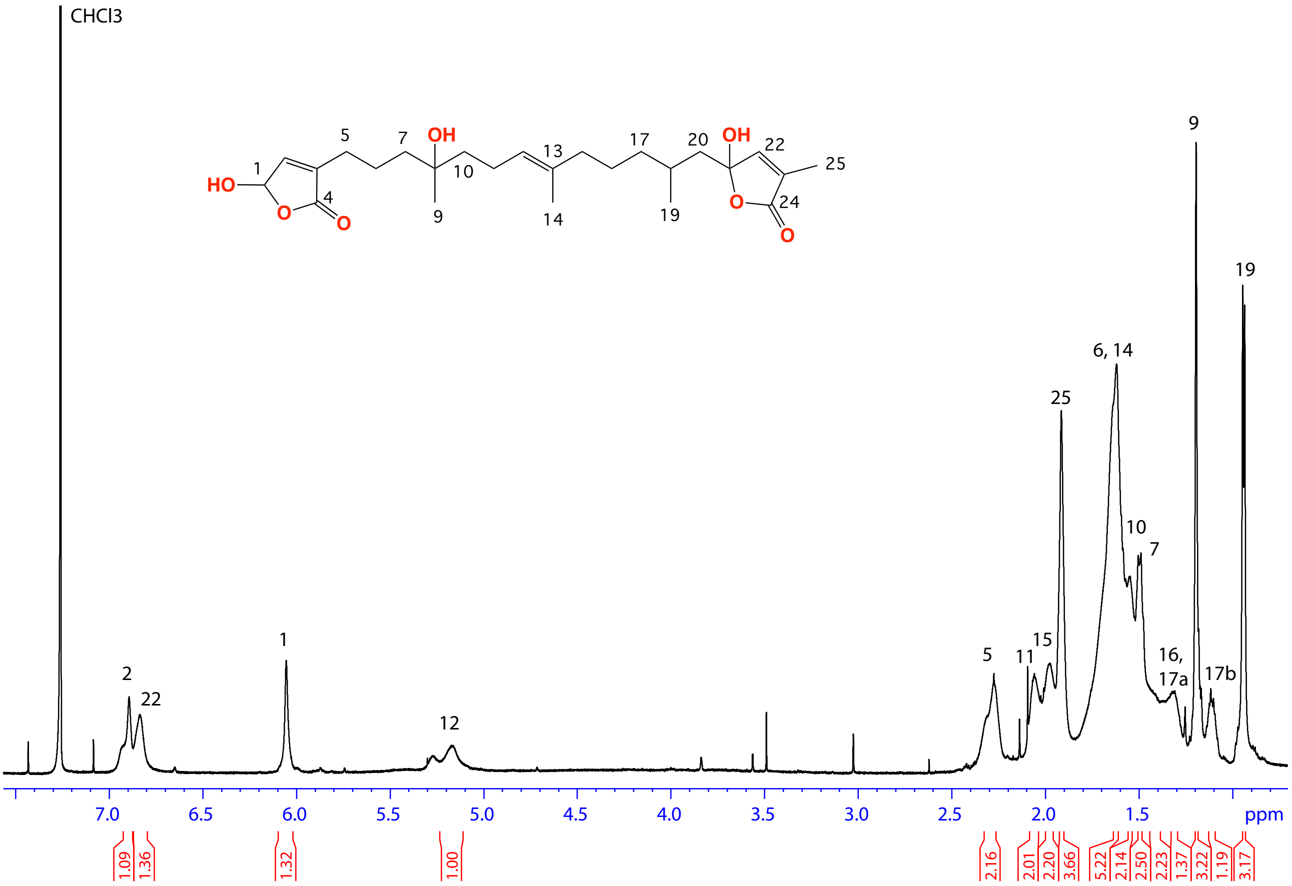

Figure S18. ^{1}H NMR (600 MHz, $CDCl_3$) spectrum of cacolide F (**6**)

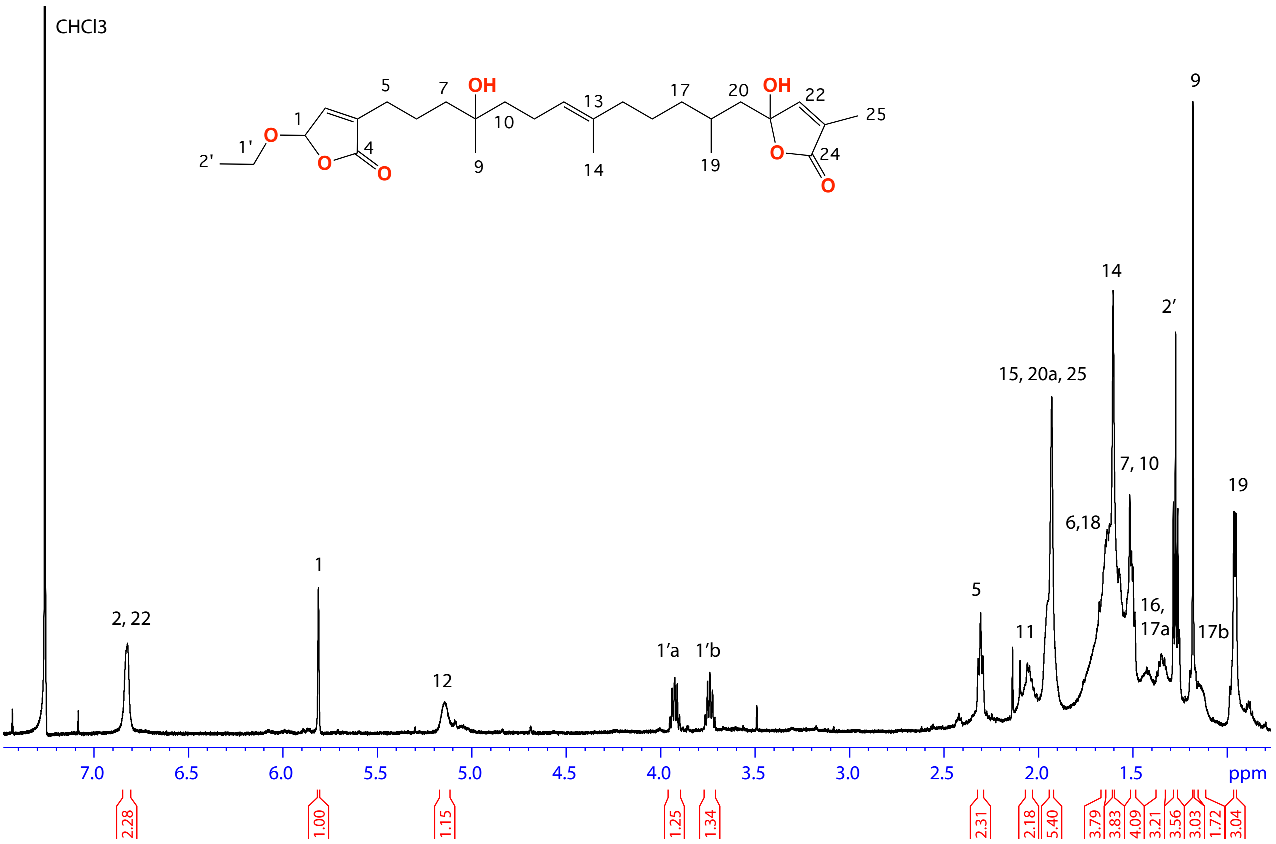

Figure S19. ^{1}H NMR (600 MHz, $CDCl_3$) spectrum of cacolide G (**7**)

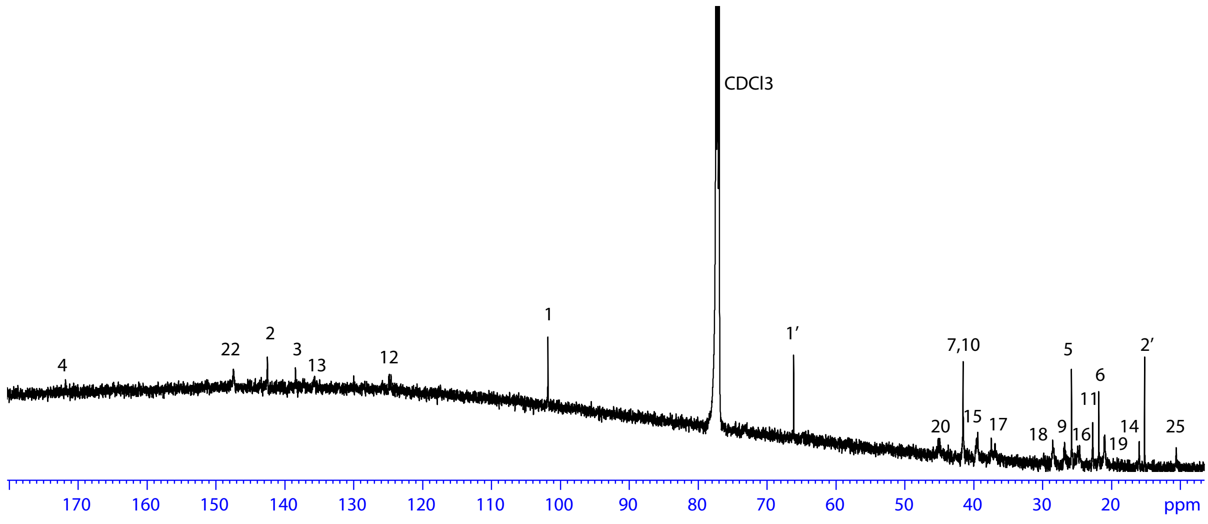

Figure S20. ^{13}C NMR (150 MHz, $CDCl_3$) spectrum of cacolide G (**7**)

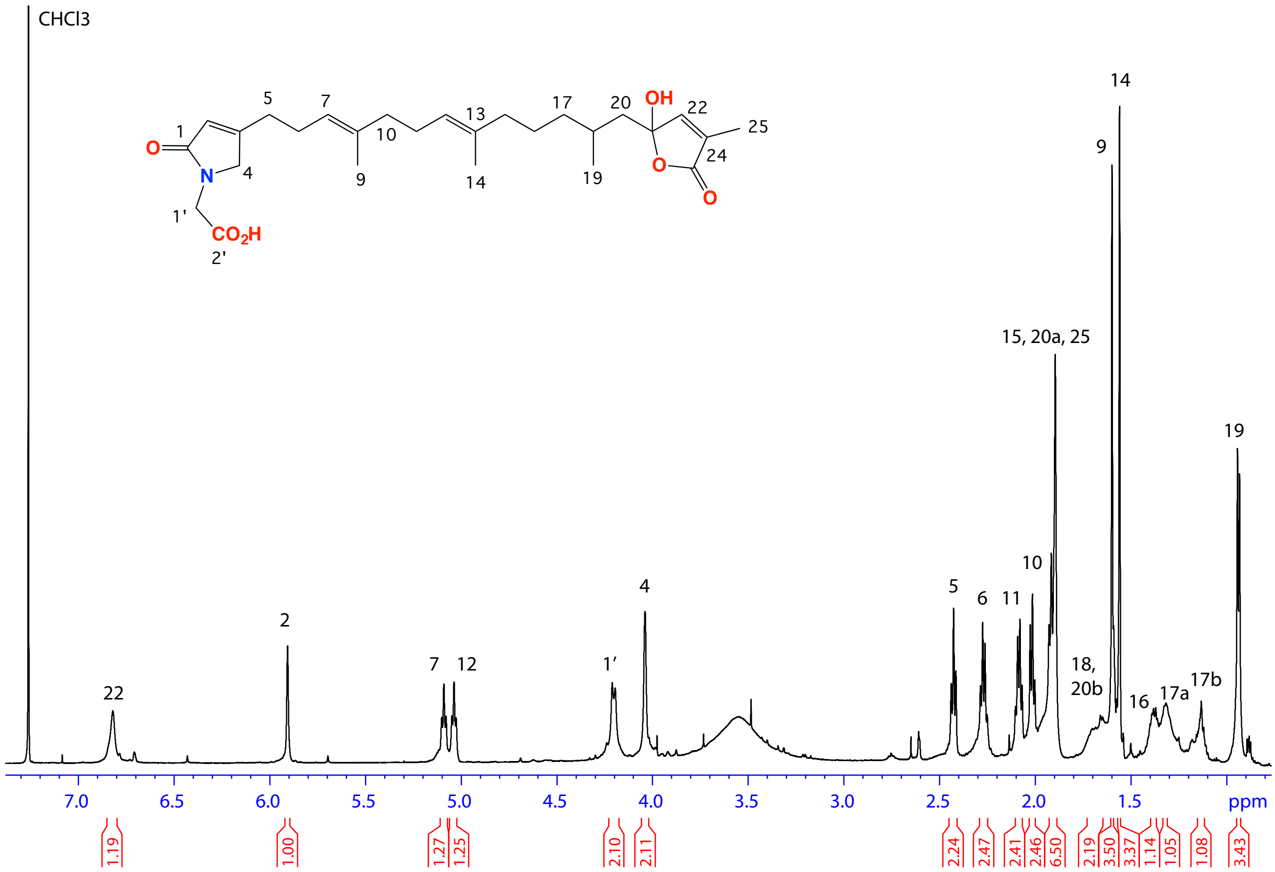

Figure S21. ^{1}H NMR (600 MHz, CDCl$_3$) spectrum of cacolide H (**8**)

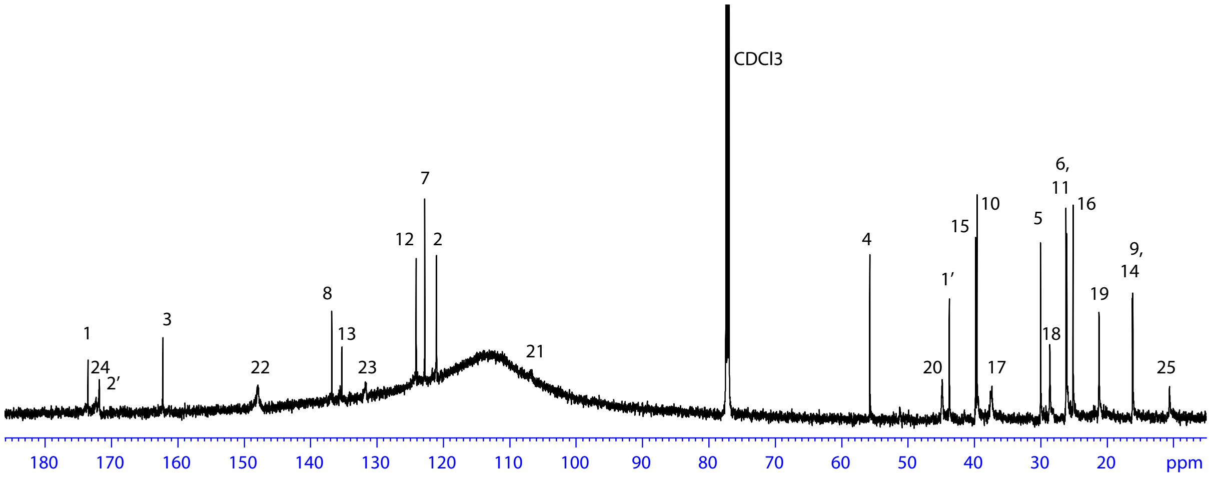

Figure S22. ^{13}C NMR (150 MHz, CDCl$_3$) spectrum of cacolide H (**8**)

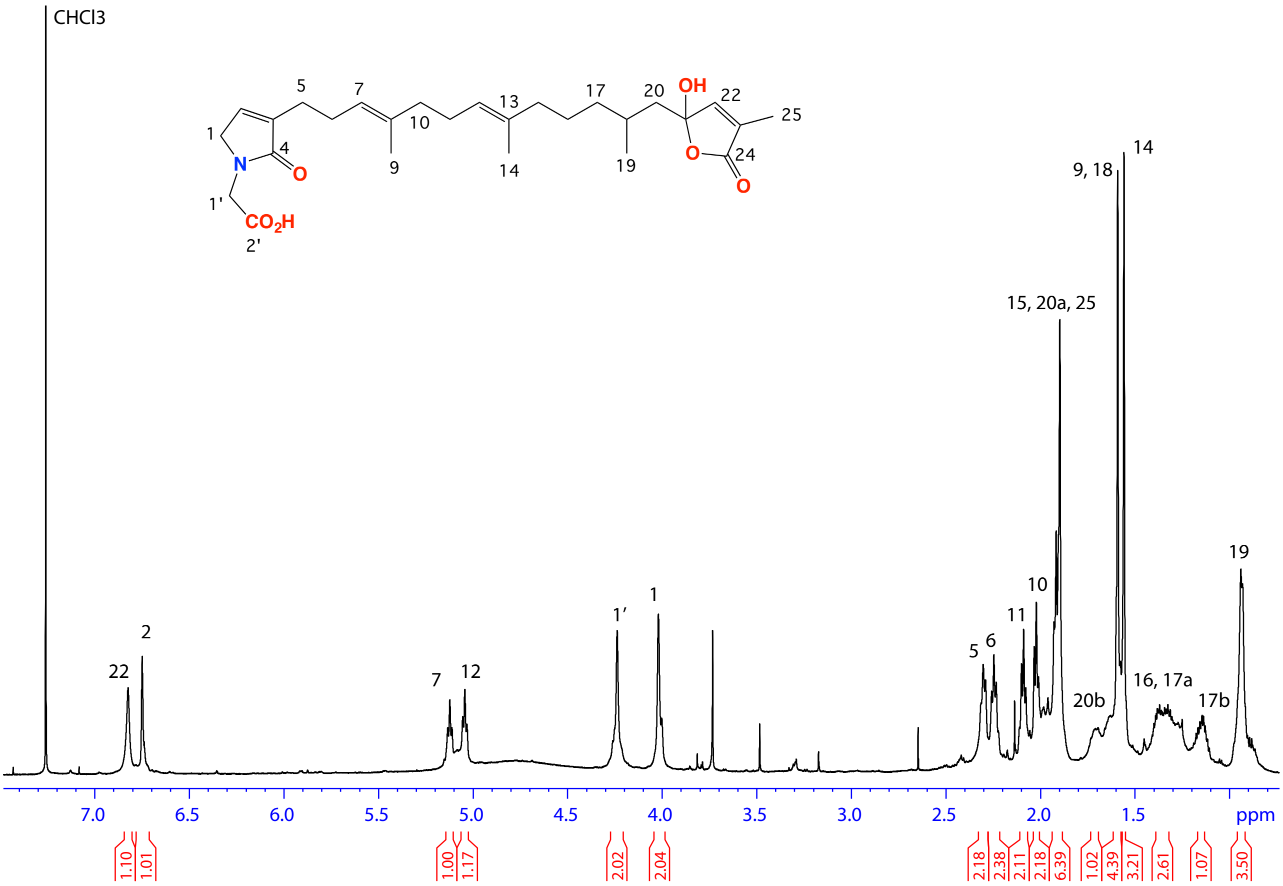

Figure S23. ^{1}H NMR (600 MHz, $CDCl_3$) spectrum of cacolide I (**9**)

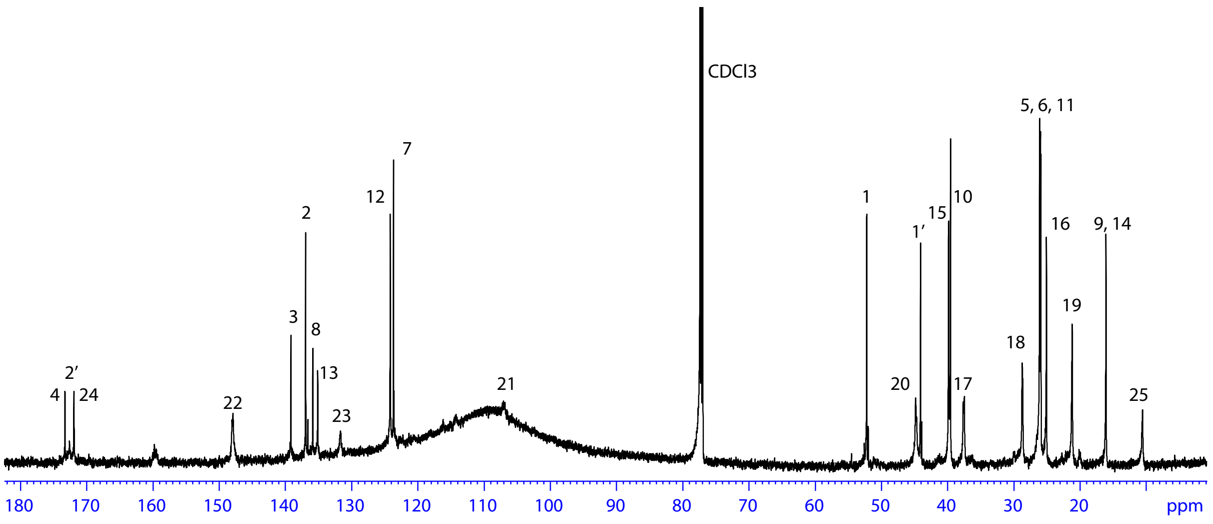

Figure S24. ^{13}C NMR (150 MHz, $CDCl_3$) spectrum of cacolide I (**9**)

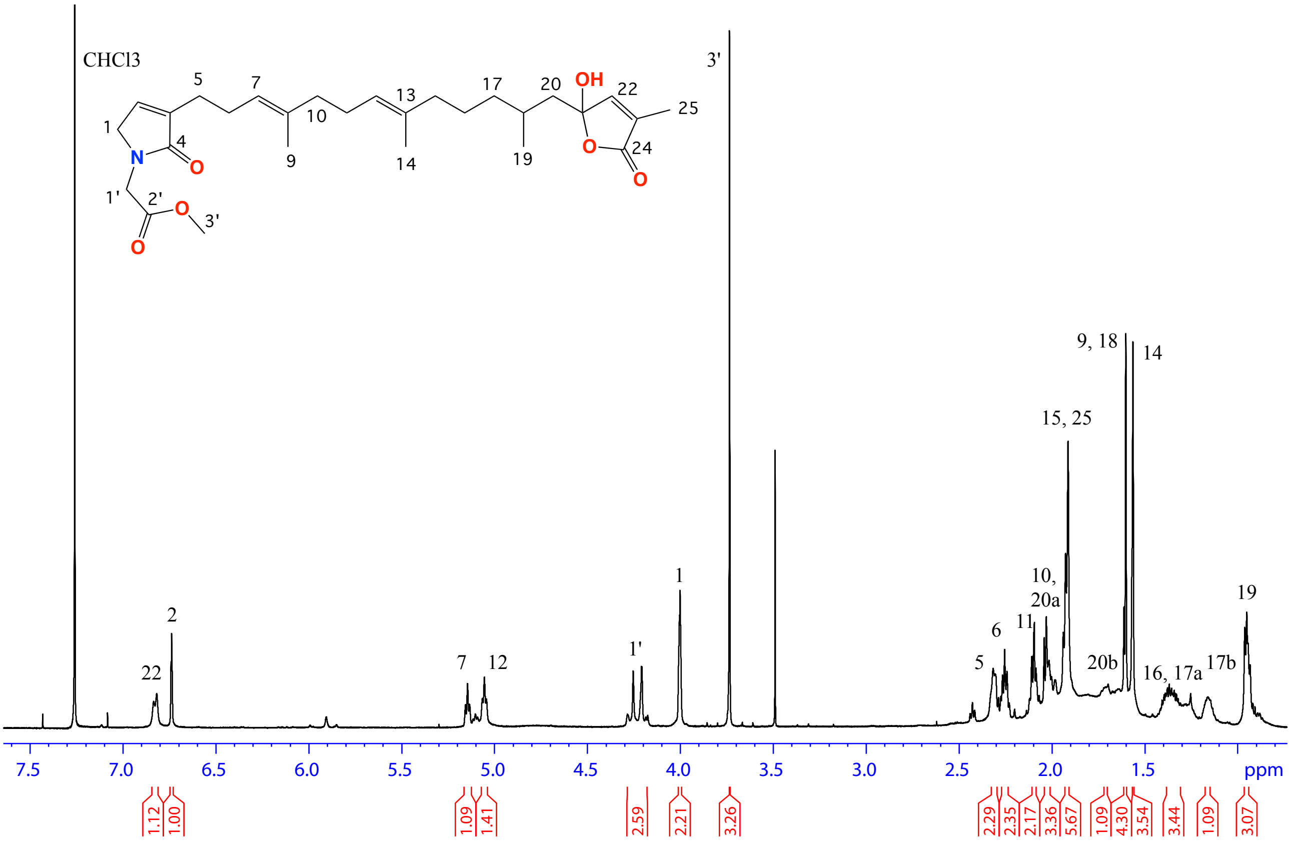

Figure S25. ^{1}H NMR (600 MHz, $CDCl_3$) spectrum of cacolide J (**10**)

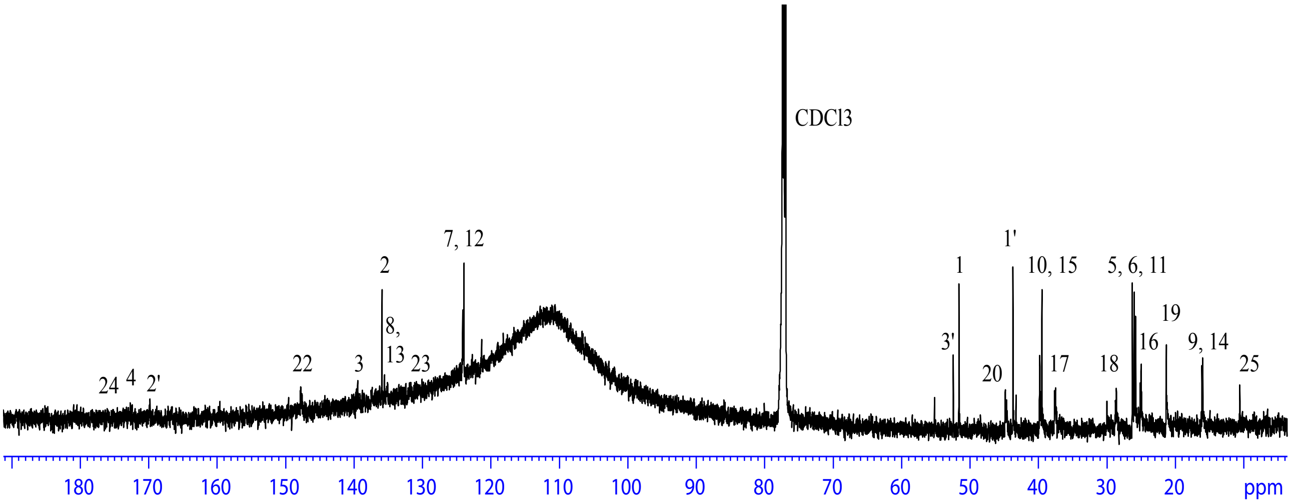

Figure S26. ^{13}C NMR (150 MHz, $CDCl_3$) spectrum of cacolide J (**10**)

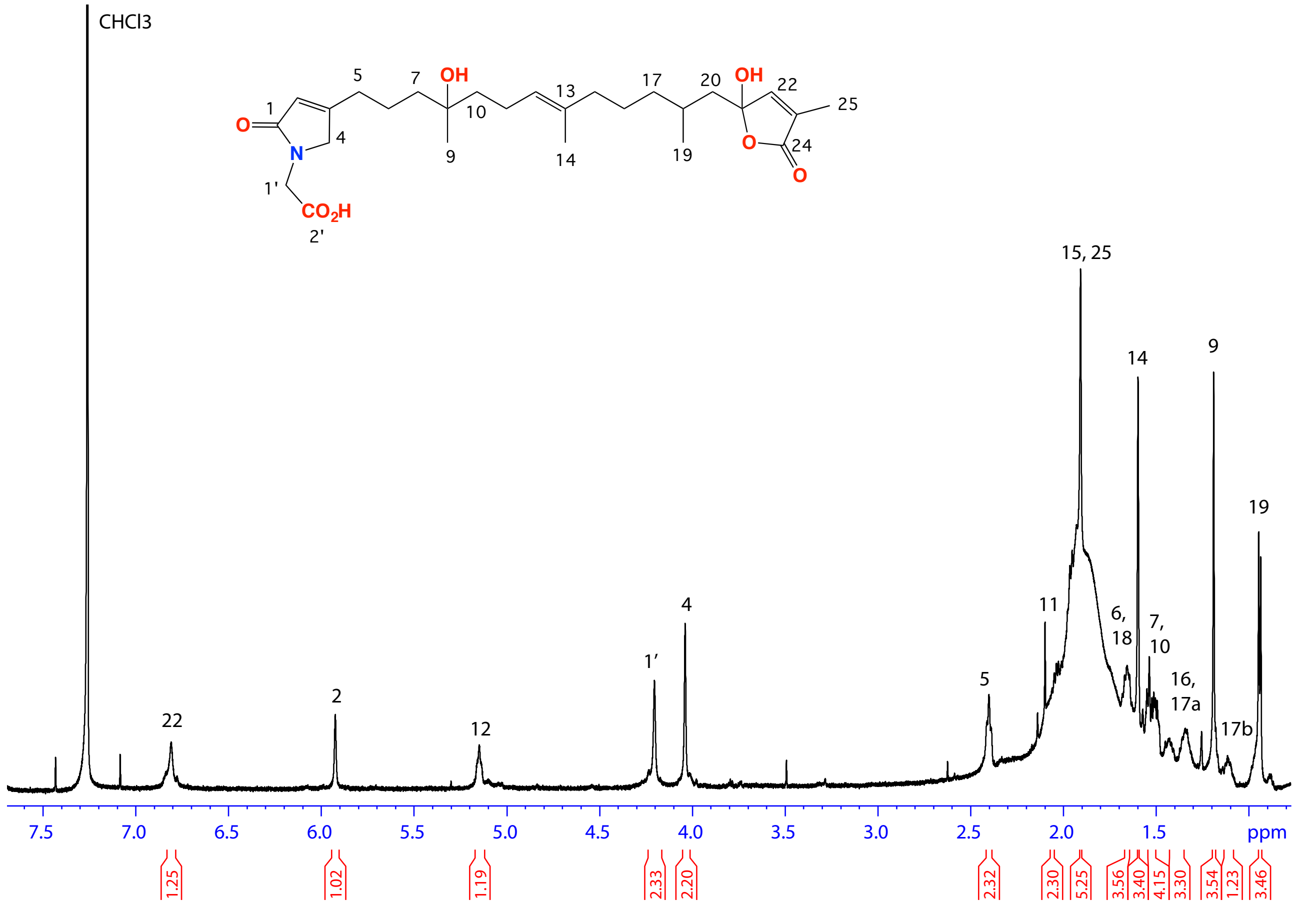

Figure S27. ^{1}H NMR (600 MHz, $CDCl_3$) spectrum of cacolide K (**11**)

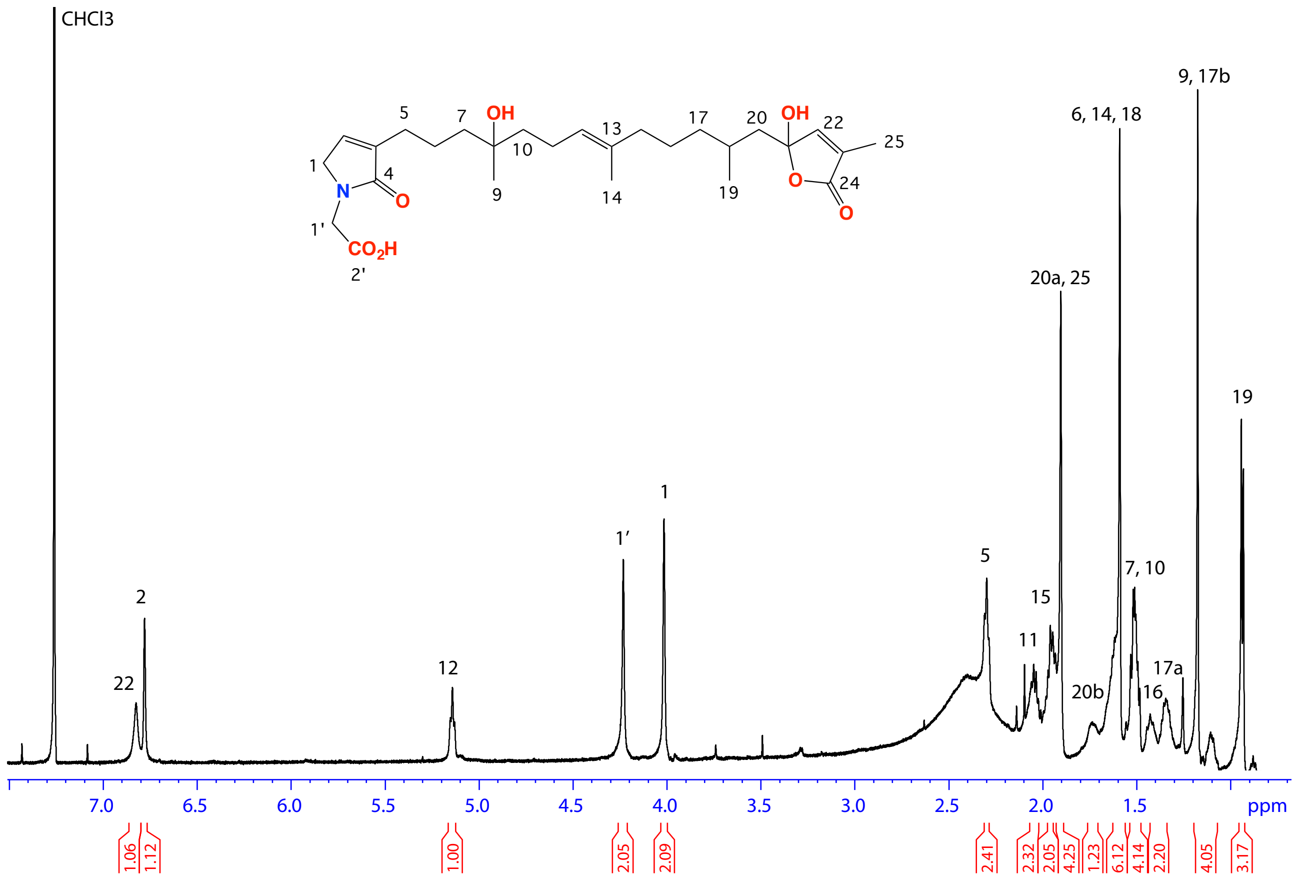

Figure S28. ^{1}H NMR (600 MHz, $CDCl_3$) spectrum of cacolide L (**12**)

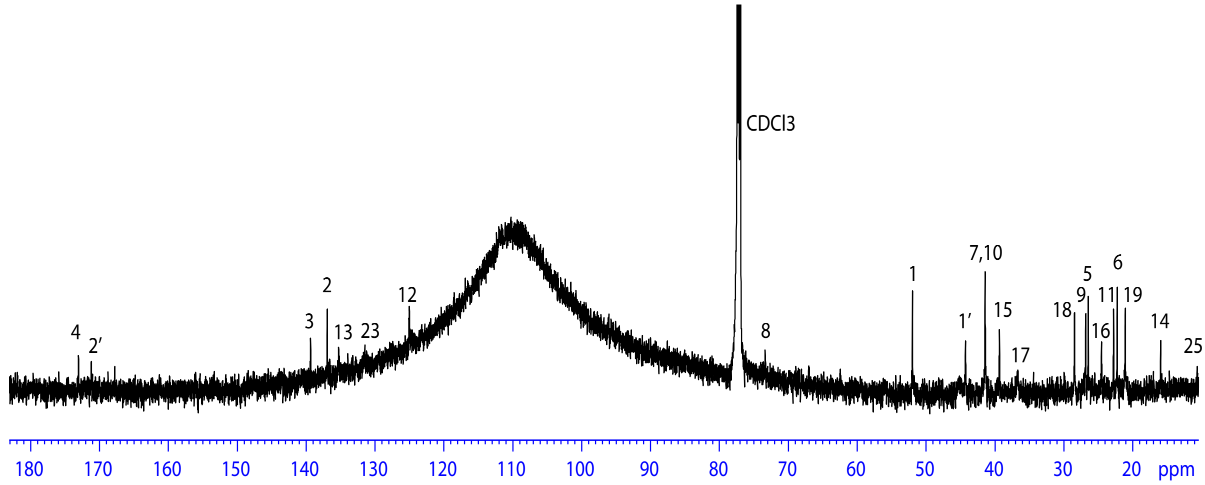

Figure S29. ^{13}C NMR (150 MHz, $CDCl_3$) spectrum of cacolide L (**12**)

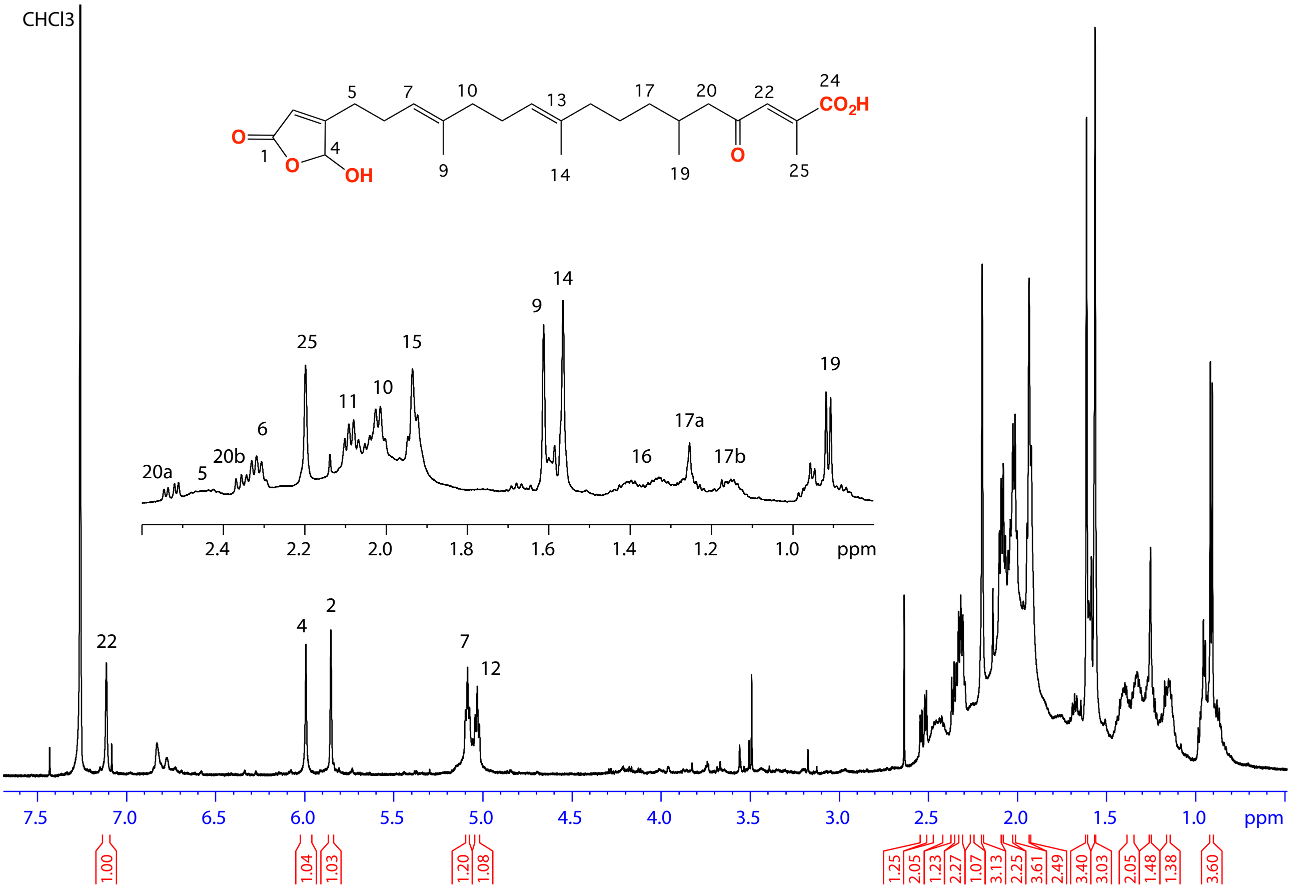

Figure S30. ^{1}H NMR (600 MHz, $CDCl_3$) spectrum of cacolic acid A (**13**)

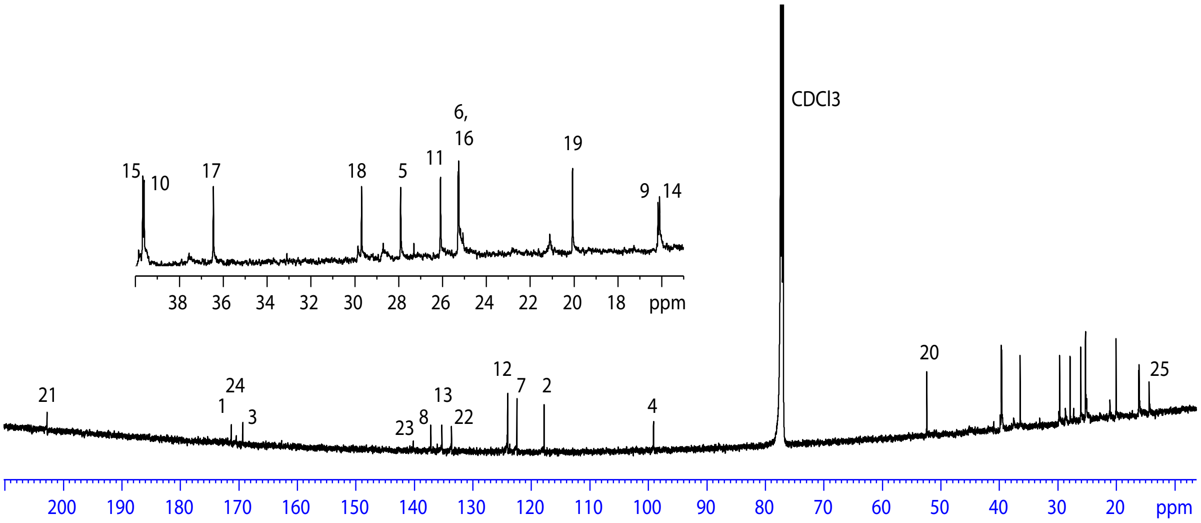

Figure S31. ^{13}C NMR (150 MHz, $CDCl_3$) spectrum of cacolic acid A (**13**)

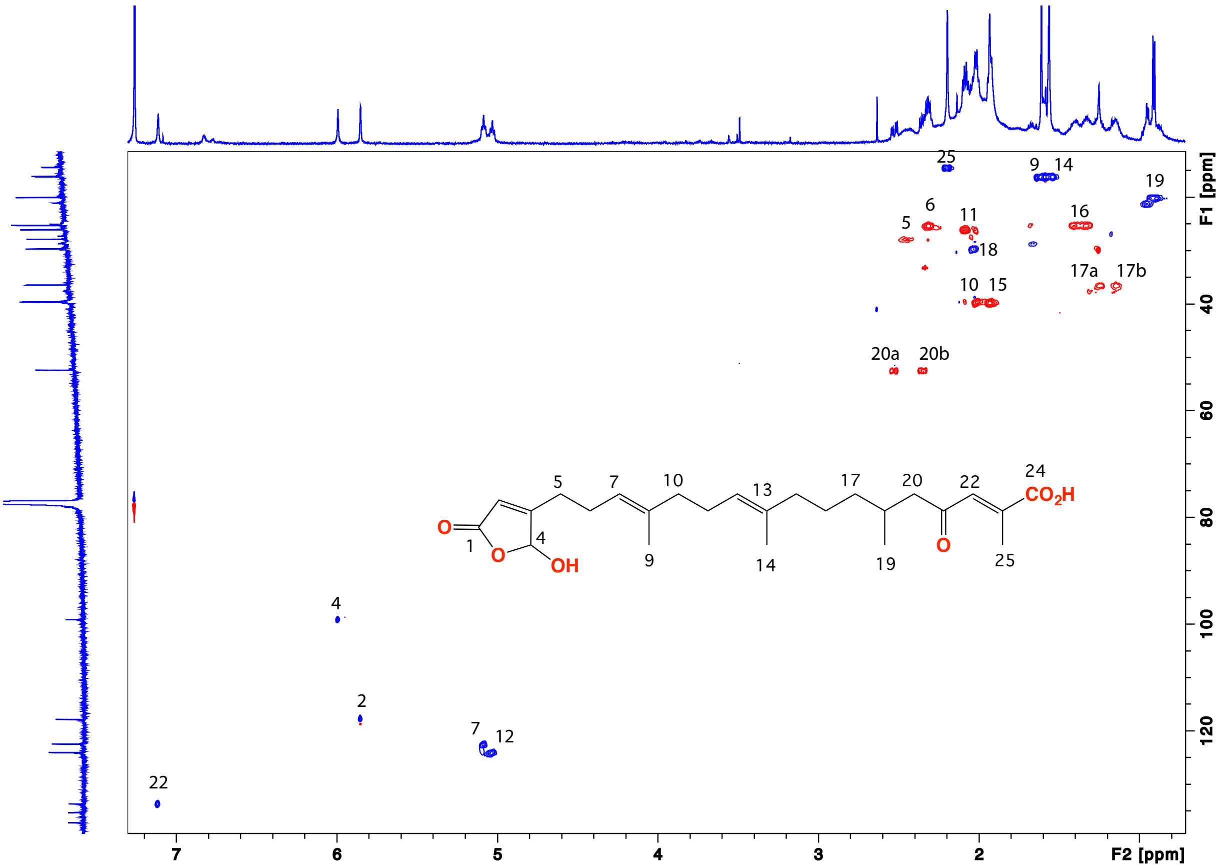

Figure S32. HSQC NMR ($CDCl_3$) spectrum of cacolic acid A (**13**)

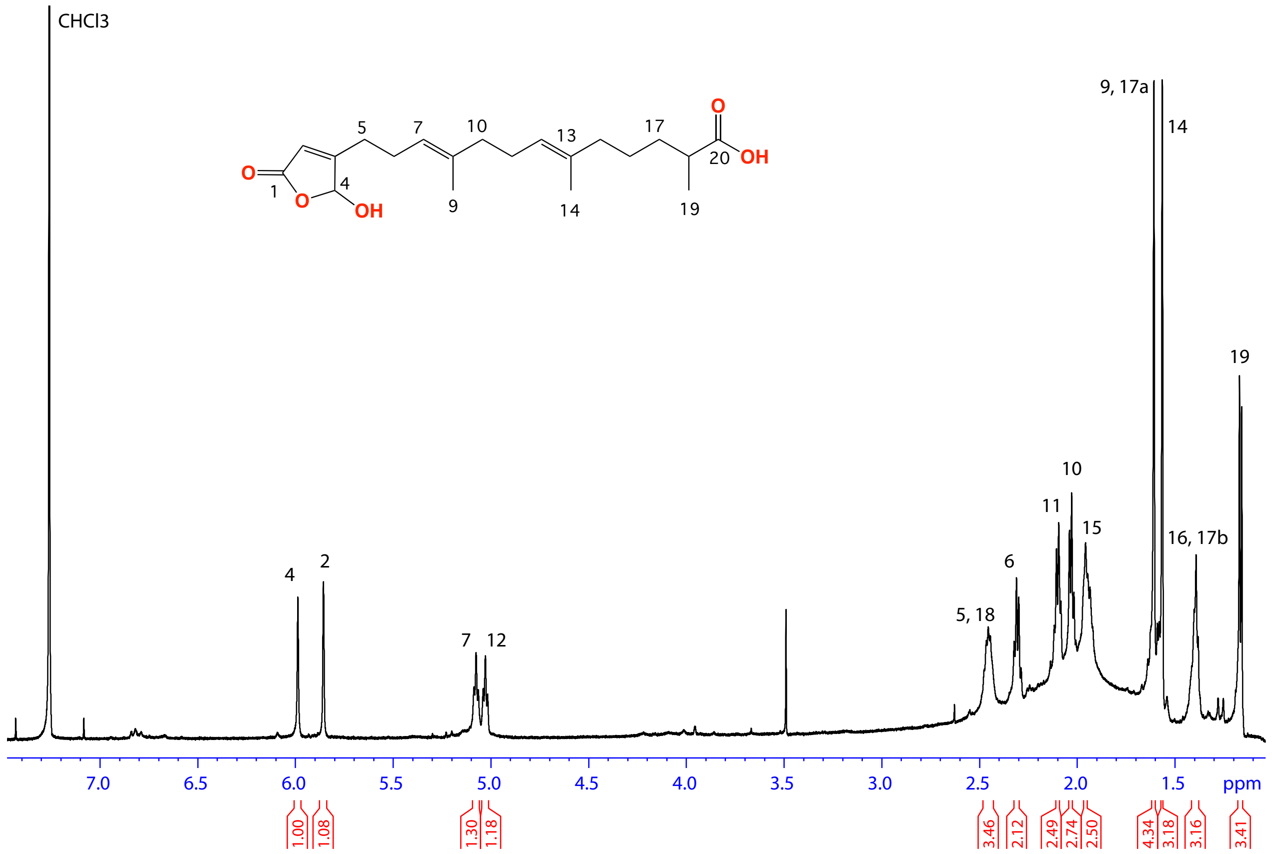

Figure S33. ^{1}H NMR (600 MHz, $CDCl_3$) spectrum of cacolic acid B (**14**)

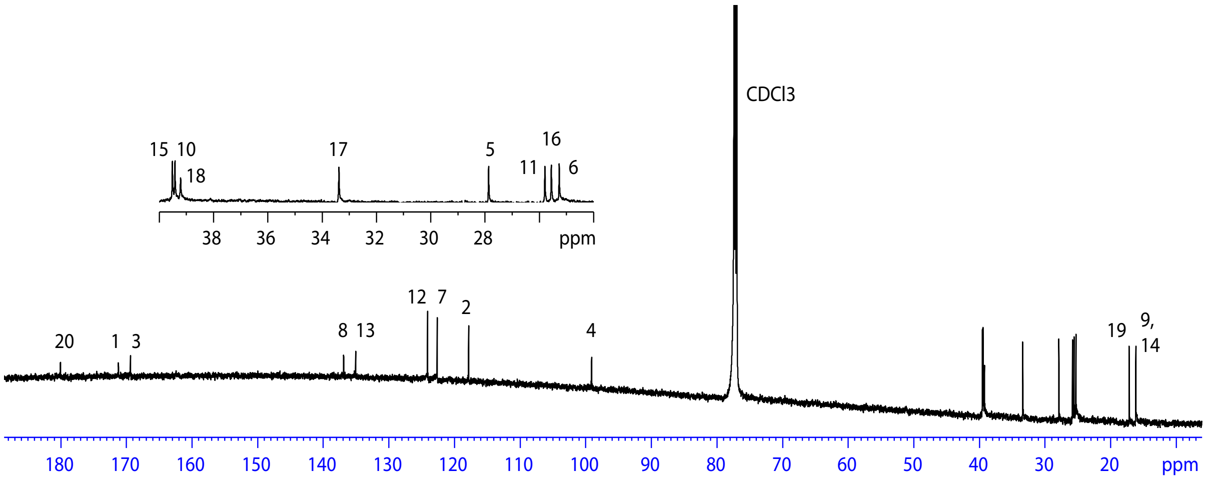

Figure S34. ^{13}C NMR (150 MHz, $CDCl_3$) spectrum of cacolic acid B (**14**)

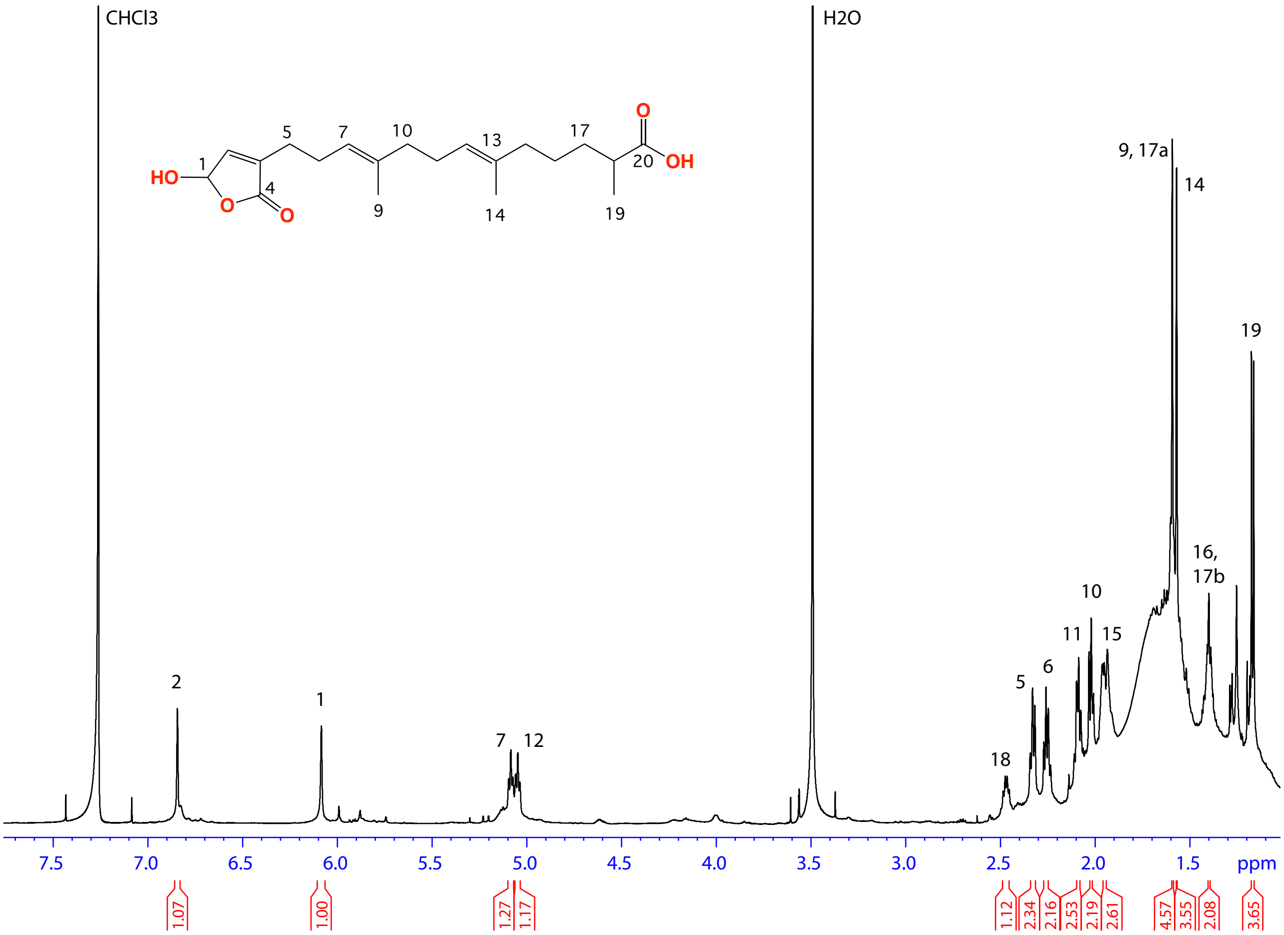

Figure S35. ^{1}H NMR (600 MHz, $CDCl_3$) spectrum of cacolic acid C (**15**)

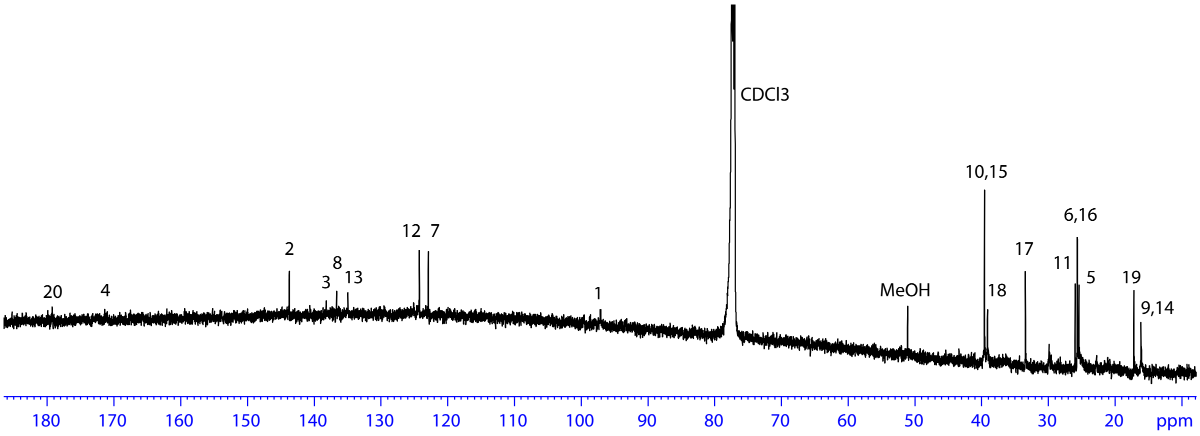

Figure S36. ^{13}C NMR (150 MHz, $CDCl_3$) spectrum of cacolic acid C (**15**)

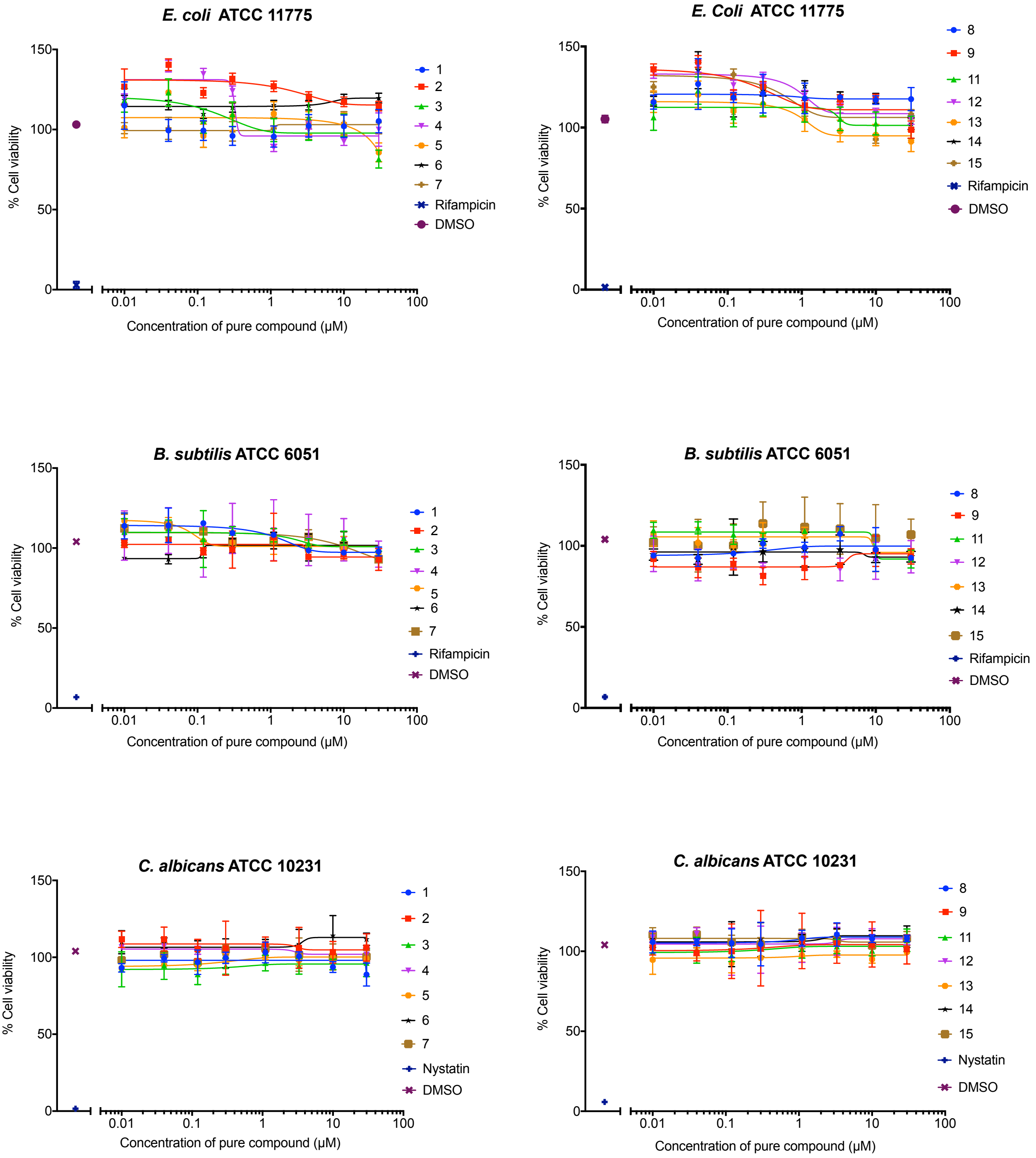

Figure S37. Antimicrobial activity of cacolides **A-I** (**1-9**), K-L (**11-12**) and cacolic acids A-C (**13-15**)

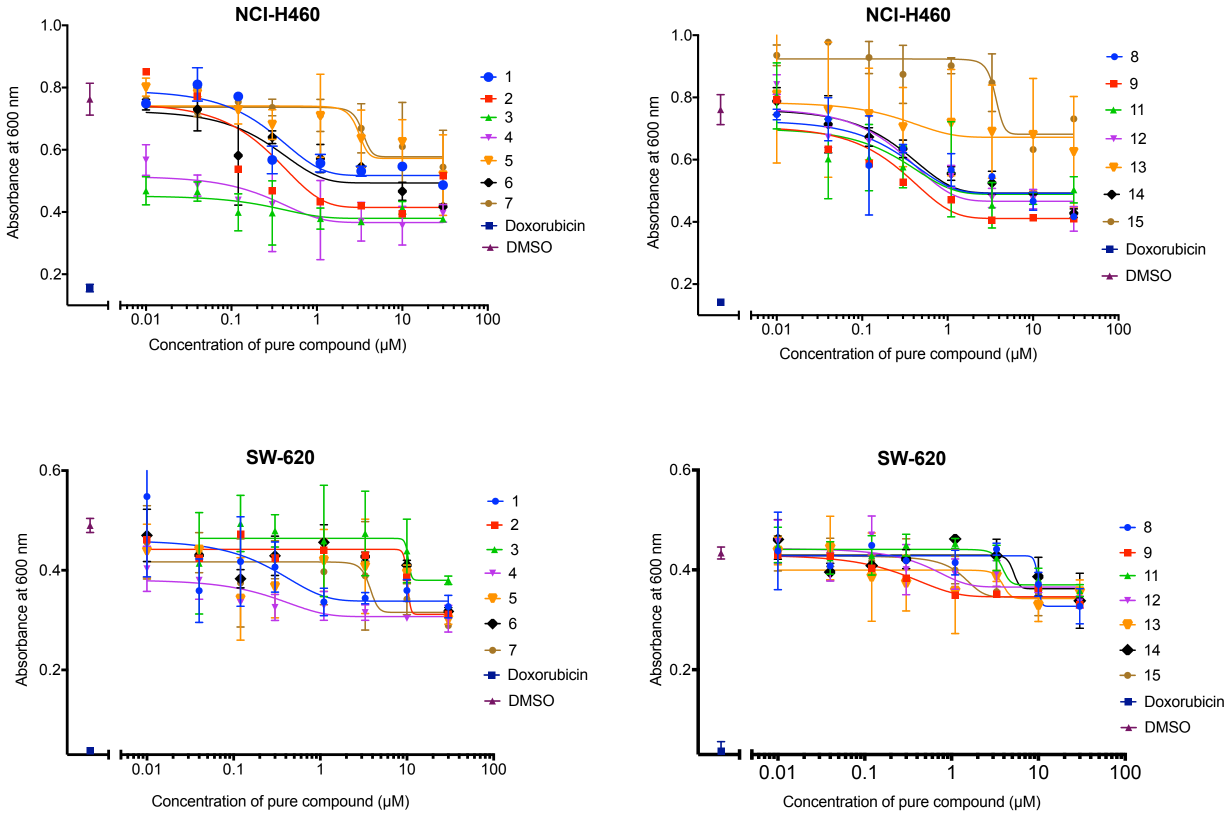

Figure S38. Cytotoxic activity of cacolides A-I (**1**-**9**), K-L (**11**-**12**) and cacolic acids A-C (**13**-**15**) against human colorectal (SW620) and lung (NCI-H460) carcinoma cells

Mass Spectrum SmartFormula Report

Analysis Info Acquisition Date 4/18/2018 12:37:50 PM

Analysis Name	D:\Data\s.khushi\CMB-03404-1-2-10-2.d		
Method	tune-med_AP.m	Operator	a.piggott
Sample Name		Instrument	micrOTOF 213750.00232
Comment			

Acquisition Parameter

Source Type	ESI	Ion Polarity	Positive	Set Nebulizer	0.8 Bar
Focus	Not active			Set Dry Heater	180 °C
Scan Begin	100 m/z	Set Capillary	4500 V	Set Dry Gas	5.0 l/min
Scan End	1000 m/z	Set End Plate Offset	-500 V	Set Divert Valve	Source

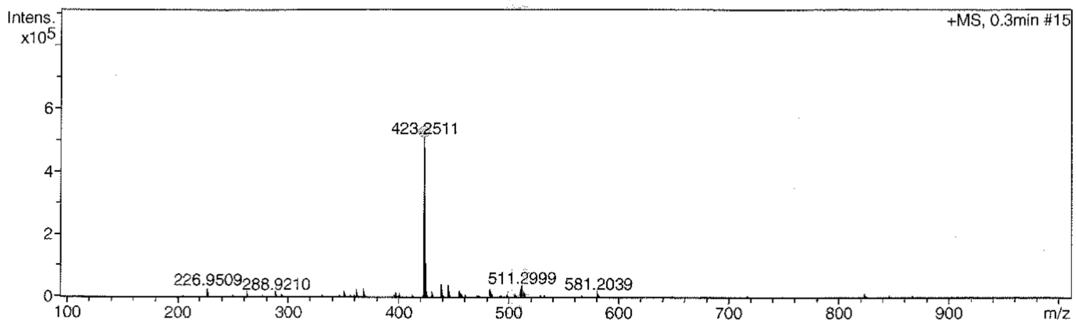

Meas. m/z	#	Ion Formula	m/z	err [ppm]	mSigma	# Sigma	Score	rdb	e⁻ Conf	N-Rule
423.2511	1	C25H36NaO4	423.2506	-1.2	3.7	1	100.00	7.5	even	ok

Figure S39. HR-ESIMS data for cacolide A (**1**)

Mass Spectrum SmartFormula Report

Analysis Info Acquisition Date 4/18/2018 12:09:59 PM

Analysis Name	D:\Data\s.khushi\CMB-03404-1-2-6-5.d		
Method	tune-med_AP.m	Operator	a.piggott
Sample Name		Instrument	micrOTOF 213750.00232
Comment			

Acquisition Parameter

Source Type	ESI	Ion Polarity	Positive	Set Nebulizer	0.8 Bar
Focus	Not active			Set Dry Heater	180 °C
Scan Begin	100 m/z	Set Capillary	4500 V	Set Dry Gas	5.0 l/min
Scan End	1000 m/z	Set End Plate Offset	-500 V	Set Divert Valve	Source

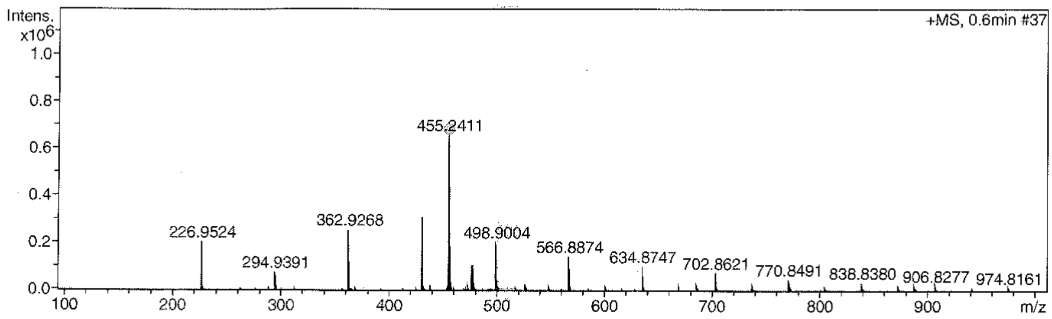

Meas. m/z	#	Ion Formula	m/z	err [ppm]	mSigma	# Sigma	Score	rdb	e⁻ Conf	N-Rule
455.2411	1	C25H36NaO6	455.2404	1.5	0.7	1	100.00	7.5	even	ok

Figure S40. HR-ESIMS data for cacolide B (**2**)

Mass Spectrum SmartFormula Report

**Analysis Info**		Acquisition Date	4/18/2018 12:19:20 PM
Analysis Name	D:\Data\s.khushi\CMB-03404-1-2-6-6.d		
Method	tune-med_AP.m	Operator	a.piggott
Sample Name		Instrument	micrOTOF 213750.00232
Comment			

Acquisition Parameter

Source Type	ESI	Ion Polarity	Positive	Set Nebulizer	0.8 Bar
Focus	Not active			Set Dry Heater	180 °C
Scan Begin	100 m/z	Set Capillary	4500 V	Set Dry Gas	5.0 l/min
Scan End	1000 m/z	Set End Plate Offset	-500 V	Set Divert Valve	Source

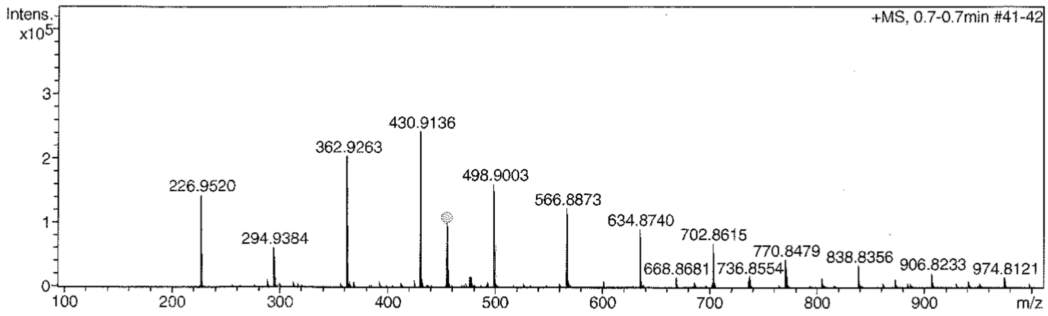

Meas. m/z	#	Ion Formula	m/z	err [ppm]	mSigma	# Sigma	Score	rdb	e⁻ Conf	N-Rule
455.2398	1	C25H36NaO6	455.2404	-1.3	5.4	1	100.00	7.5	even	ok

Figure S41. HR-ESIMS data for cacolide C (**3**)

Mass Spectrum SmartFormula Report

**Analysis Info**		Acquisition Date	4/18/2018 12:56:36 PM
Analysis Name	D:\Data\s.khushi\CMB-03404-1-2-8-4.d		
Method	tune-med_AP.m	Operator	a.piggott
Sample Name		Instrument	micrOTOF 213750.00232
Comment			

Acquisition Parameter

Source Type	ESI	Ion Polarity	Positive	Set Nebulizer	0.8 Bar
Focus	Not active			Set Dry Heater	180 °C
Scan Begin	100 m/z	Set Capillary	4500 V	Set Dry Gas	5.0 l/min
Scan End	1000 m/z	Set End Plate Offset	-500 V	Set Divert Valve	Source

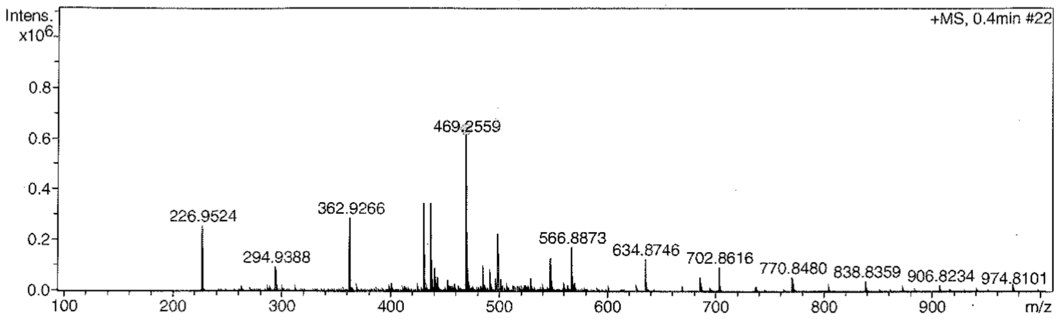

Meas. m/z	#	Ion Formula	m/z	err [ppm]	mSigma	# Sigma	Score	rdb	e⁻ Conf	N-Rule
469.2559	1	C26H38NaO6	469.2561	0.4	24.3	1	100.00	7.5	even	ok

Figure S42. HR-ESIMS data for cacolide D (**4**)

Mass Spectrum SmartFormula Report

**Analysis Info**		Acquisition Date	4/18/2018 12:31:40 PM	
Analysis Name	D:\Data\s.khushi\CMB-03404-1-2-8-9.d			
Method	tune-med_AP.m	Operator	a.piggott	
Sample Name		Instrument	micrOTOF	213750.00232
Comment				

**Acquisition Parameter**					
Source Type	ESI	Ion Polarity	Positive	Set Nebulizer	0.8 Bar
Focus	Not active			Set Dry Heater	180 °C
Scan Begin	100 m/z	Set Capillary	4500 V	Set Dry Gas	5.0 l/min
Scan End	1000 m/z	Set End Plate Offset	-500 V	Set Divert Valve	Source

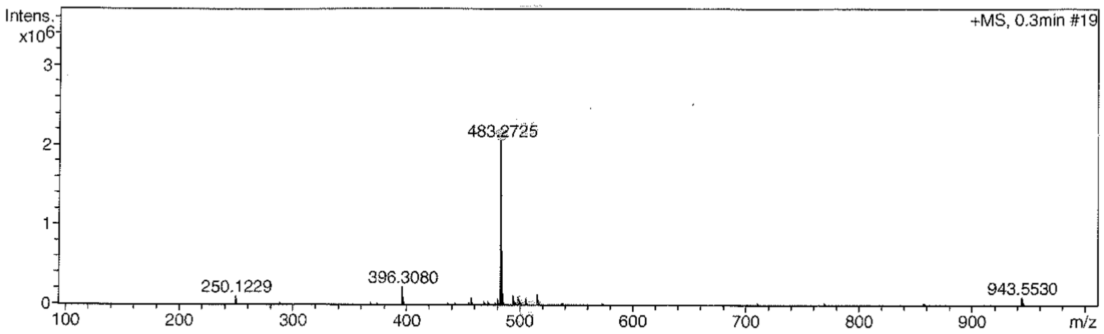

Meas. m/z	#	Ion Formula	m/z	err [ppm]	mSigma	# Sigma	Score	rdb	e⁻ Conf	N-Rule
483.2725	1	C27H40NaO6	483.2717	-1.7	15.8	1	100.00	7.5	even	ok

Figure S43. HR-ESIMS data for cacolide E (**5**)

Mass Spectrum SmartFormula Report

**Analysis Info**		Acquisition Date	4/18/2018 12:04:13 PM	
Analysis Name	D:\Data\s.khushi\CMB-03404-1-2-3-3.d			
Method	tune-med_AP.m	Operator	a.piggott	
Sample Name		Instrument	micrOTOF	213750.00232
Comment				

**Acquisition Parameter**					
Source Type	ESI	Ion Polarity	Positive	Set Nebulizer	0.8 Bar
Focus	Not active			Set Dry Heater	180 °C
Scan Begin	100 m/z	Set Capillary	4500 V	Set Dry Gas	5.0 l/min
Scan End	1000 m/z	Set End Plate Offset	-500 V	Set Divert Valve	Source

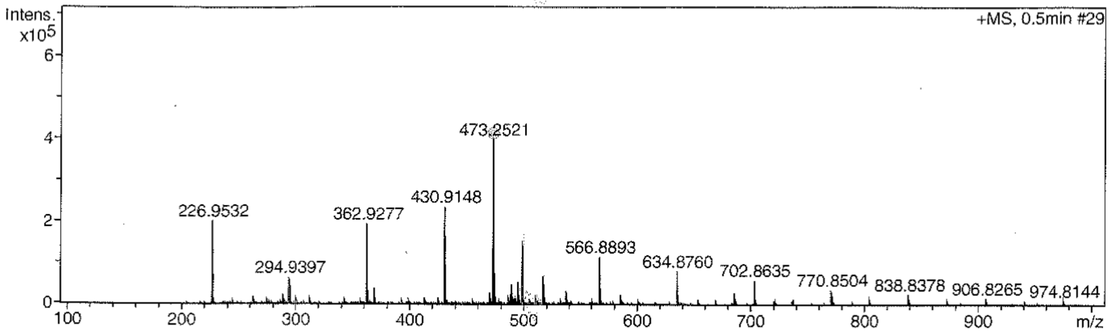

Meas. m/z	#	Ion Formula	m/z	err [ppm]	mSigma	# Sigma	Score	rdb	e⁻ Conf	N-Rule
473.2521	1	C25H38NaO7	473.2510	2.3	3.9	1	100.00	6.5	even	ok

Figure S44. HR-ESIMS data for cacolide F (**6**)

Mass Spectrum SmartFormula Report

Analysis Info

Analysis Name	D:\Data\s.khushi\CMB-03404-1-2-6-2.d	Acquisition Date	4/18/2018 12:45:23 PM
Method	tune-med_AP.m	Operator	a.piggott
Sample Name		Instrument	micrOTOF 213750.00232
Comment			

Acquisition Parameter

Source Type	ESI	Ion Polarity	Positive	Set Nebulizer	0.8 Bar
Focus	Not active			Set Dry Heater	180 °C
Scan Begin	100 m/z	Set Capillary	4500 V	Set Dry Gas	5.0 l/min
Scan End	1000 m/z	Set End Plate Offset	-500 V	Set Divert Valve	Source

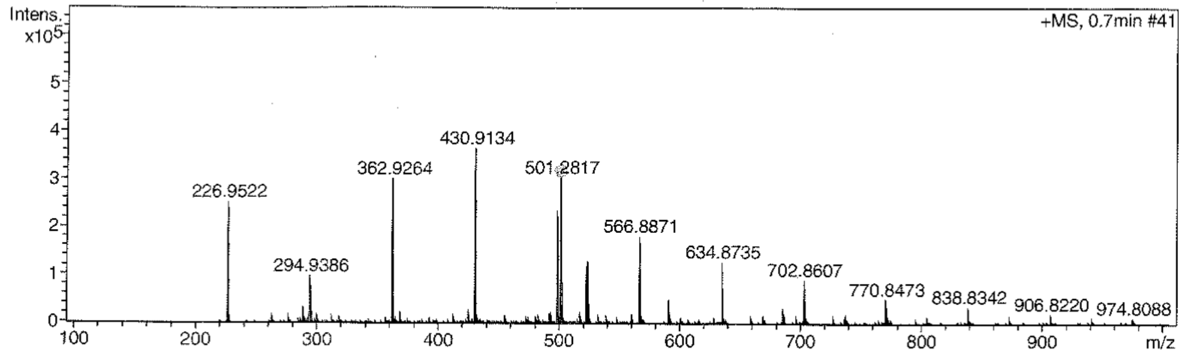

Meas. m/z	#	Ion Formula	m/z	err [ppm]	mSigma	# Sigma	Score	rdb	e⁻ Conf	N-Rule
501.2817	1	C27H42NaO7	501.2823	1.2	7.3	1	100.00	6.5	even	ok

Figure S45. HR-ESIMS data for cacolide G (**7**)

Mass Spectrum SmartFormula Report

Analysis Info

Analysis Name	D:\Data\s.khushi\CMB-03404-1-2-5-5.d	Acquisition Date	4/18/2018 11:59:07 AM
Method	tune-med_AP.m	Operator	a.piggott
Sample Name		Instrument	micrOTOF 213750.00232
Comment			

Acquisition Parameter

Source Type	ESI	Ion Polarity	Positive	Set Nebulizer	0.8 Bar
Focus	Not active			Set Dry Heater	180 °C
Scan Begin	100 m/z	Set Capillary	4500 V	Set Dry Gas	5.0 l/min
Scan End	1000 m/z	Set End Plate Offset	-500 V	Set Divert Valve	Source

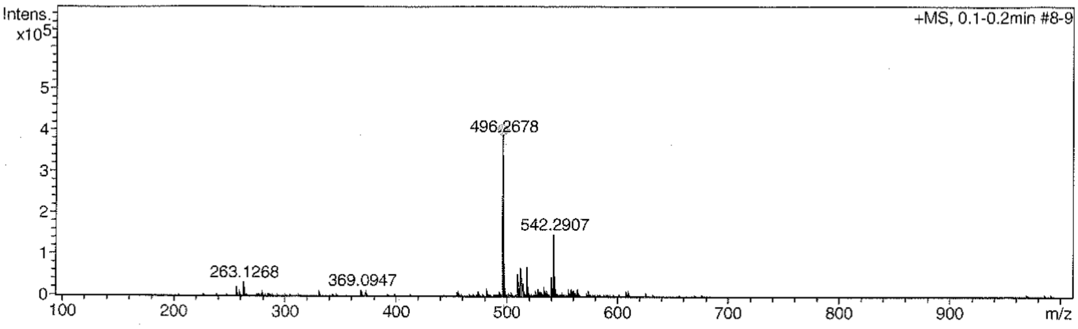

Meas. m/z	#	Ion Formula	m/z	err [ppm]	mSigma	# Sigma	Score	rdb	e⁻ Conf	N-Rule
496.2678	1	C27H39NNaO6	496.2670	-1.6	4.5	1	100.00	8.5	even	ok

Figure S46. HR-ESIMS data for cacolide H (**8**)

Mass Spectrum SmartFormula Report

Analysis Info

Analysis Name	D:\Data\s.khushi\CMB-03404-1-2-9.d	Acquisition Date	4/18/2018 11:30:41 AM
Method	tune-med_AP.m	Operator	a.piggott
Sample Name		Instrument	micrOTOF 213750.00232
Comment			

Acquisition Parameter

Source Type	ESI	Ion Polarity	Positive	Set Nebulizer	0.8 Bar
Focus	Not active			Set Dry Heater	180 °C
Scan Begin	100 m/z	Set Capillary	4500 V	Set Dry Gas	5.0 l/min
Scan End	1000 m/z	Set End Plate Offset	-500 V	Set Divert Valve	Source

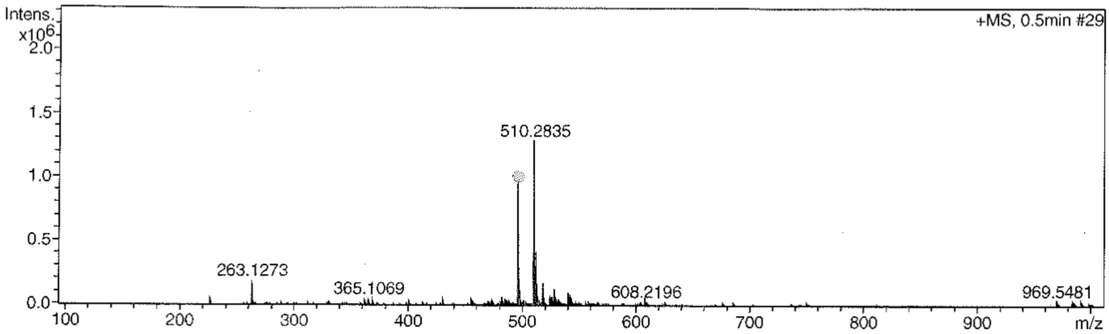

Meas. m/z	#	Ion Formula	m/z	err [ppm]	mSigma	# Sigma	Score	rdb	e⁻ Conf	N-Rule
496.2682	1	C27H39NNaO6	496.2670	2.4	12.4	1	100.00	8.5	even	ok

Figure S47. HR-ESIMS data for cacolide I (**9**)

Mass Spectrum Molecular Formula Report

Analysis Info

Analysis Name	D:\Data\s.khushi\CMB-03404-1-3-3-4-2nd.d	Acquisition Date	10/5/2018 8:08:27 PM
Method	tune-med_AP.m	Operator	a.piggott
Sample Name		Instrument / Ser#	micrOTOF 213750.00232
Comment			

Acquisition Parameter

Source Type	ESI	Ion Polarity	Positive	Set Nebulizer	0.8 Bar
Focus	Not active			Set Dry Heater	180 °C
Scan Begin	100 m/z	Set Capillary	4500 V	Set Dry Gas	5.0 l/min
Scan End	1000 m/z	Set End Plate Offset	-500 V	Set Divert Valve	Source

Generate Molecular Formula Parameter

Formula, min.		
Formula, max.		
Measured m/z	Tolerance	Charge
Check Valence	Minimum	Maximum
Nirogen Rule	Electron Configuration	
Filter H/C Ratio	Minimum	Maximum
Estimate Carbon		

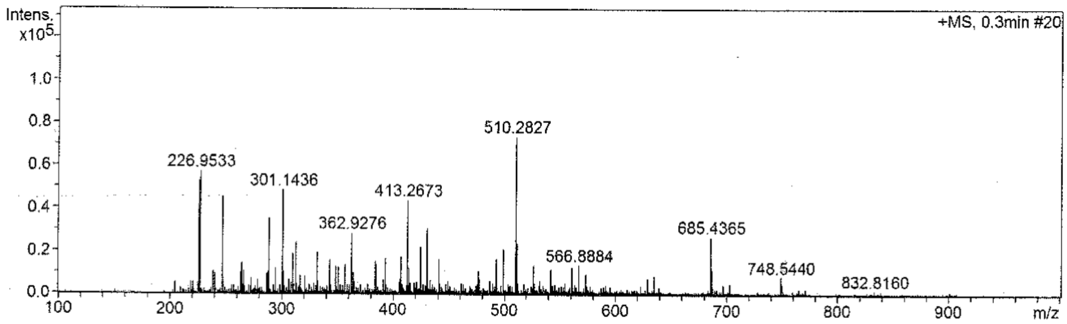

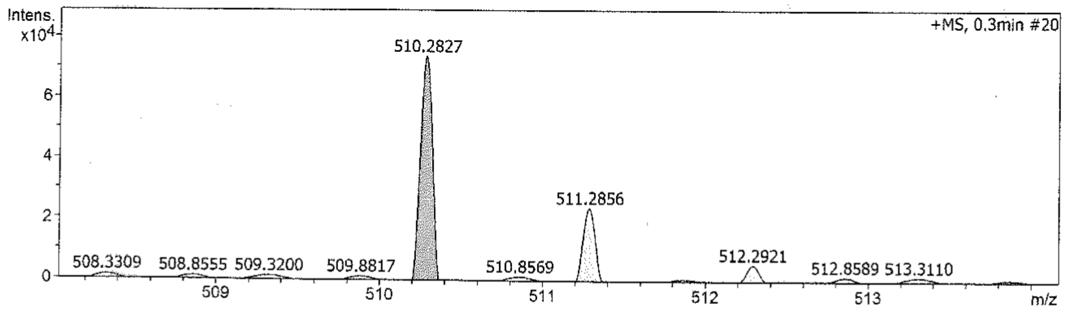

Meas. m/z	#	Ion Formula	m/z	err [ppm]	mSigma	# Sigma	Score	rdb	e⁻ Conf	N-Rule
510.2827	1	C28H41NNaO6	510.2826	0.3	11.0	1	100.00	8.5	even	ok

Figure S48. HR-ESIMS data for cacolide J (**10**)

Mass Spectrum SmartFormula Report

**Analysis Info**		Acquisition Date	4/18/2018 12:52:34 PM	
Analysis Name	D:\Data\s.khushi\CMB-03404-1-2-2-4.d			
Method	tune-med_AP.m	Operator	a.piggott	
Sample Name		Instrument	micrOTOF	213750.00232
Comment				

**Acquisition Parameter**					
Source Type	ESI	Ion Polarity	Positive	Set Nebulizer	0.8 Bar
Focus	Not active			Set Dry Heater	180 °C
Scan Begin	100 m/z	Set Capillary	4500 V	Set Dry Gas	5.0 l/min
Scan End	1000 m/z	Set End Plate Offset	-500 V	Set Divert Valve	Source

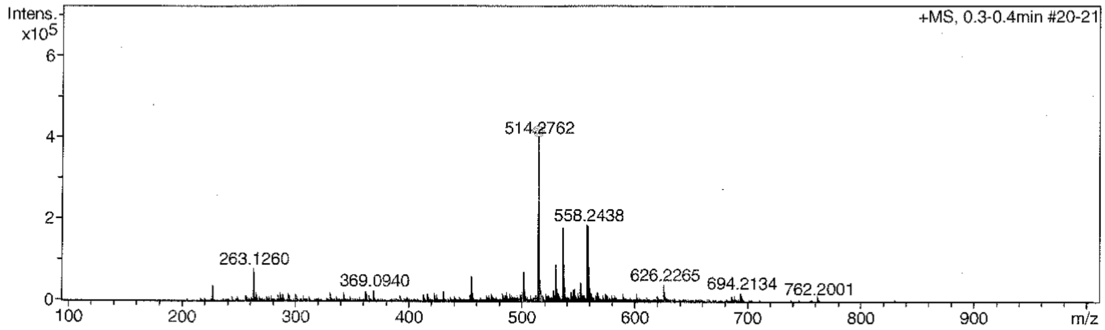

Meas. m/z	#	Ion Formula	m/z	err [ppm]	mSigma	# Sigma	Score	rdb	e⁻ Conf	N-Rule
514.2762	1	C27H41NNaO7	514.2775	2.6	20.0	1	100.00	7.5	even	ok

Figure S49. HR-ESIMS data for cacolide K (**11**)

Mass Spectrum SmartFormula Report

**Analysis Info**		Acquisition Date	4/18/2018 11:41:00 AM	
Analysis Name	D:\Data\s.khushi\CMB-03404-1-2-2-6.d			
Method	tune-med_AP.m	Operator	a.piggott	
Sample Name		Instrument	micrOTOF	213750.00232
Comment				

**Acquisition Parameter**					
Source Type	ESI	Ion Polarity	Positive	Set Nebulizer	0.8 Bar
Focus	Not active			Set Dry Heater	180 °C
Scan Begin	100 m/z	Set Capillary	4500 V	Set Dry Gas	5.0 l/min
Scan End	1000 m/z	Set End Plate Offset	-500 V	Set Divert Valve	Source

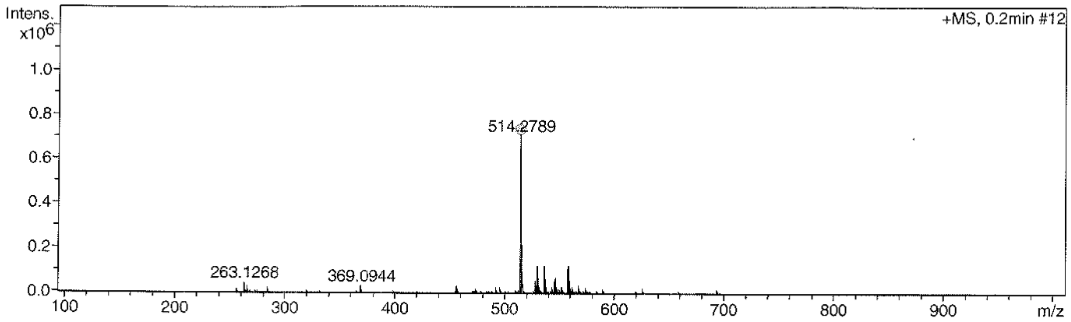

Meas. m/z	#	Ion Formula	m/z	err [ppm]	mSigma	# Sigma	Score	rdb	e⁻ Conf	N-Rule
514.2789	1	C27H41NNaO7	514.2775	-2.7	5.1	1	100.00	7.5	even	ok

Figure S50. HR-ESIMS data for cacolide L (**12**)

Mass Spectrum SmartFormula Report

Analysis Info

Analysis Name	D:\Data\s.khushi\CMB-03404-1-2-6-7.d	Acquisition Date	4/18/2018 12:26:02 PM
Method	tune-med_AP.m	Operator	a.piggott
Sample Name		Instrument	micrOTOF 213750.00232
Comment			

Acquisition Parameter

Source Type	ESI	Ion Polarity	Positive	Set Nebulizer	0.8 Bar
Focus	Not active			Set Dry Heater	180 °C
Scan Begin	100 m/z	Set Capillary	4500 V	Set Dry Gas	5.0 l/min
Scan End	1000 m/z	Set End Plate Offset	-500 V	Set Divert Valve	Source

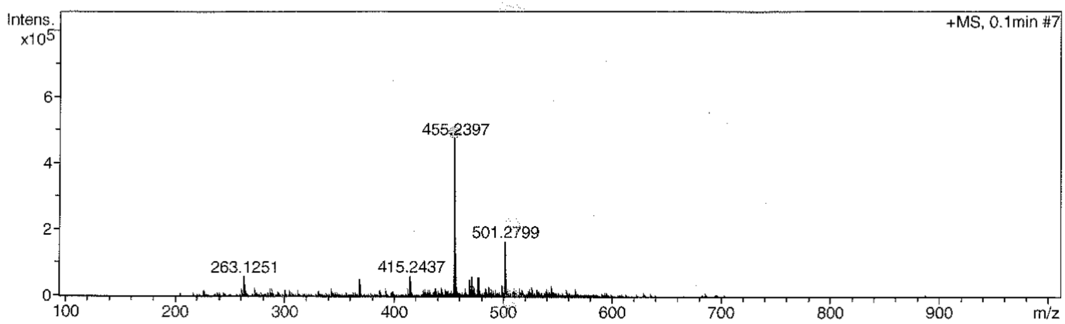

Meas. m/z	#	Ion Formula	m/z	err [ppm]	mSigma	# Sigma	Score	rdb	e⁻ Conf	N-Rule
455.2397	1	C25H36NaO6	455.2404	1.7	7.4	1	100.00	7.5	even	ok

Figure S51. HR-ESIMS data for cacolic acid A (**13**)

Mass Spectrum SmartFormula Report

Analysis Info

Analysis Name	D:\Data\s.khushi\CMB-03404-1-2-5-1.d	Acquisition Date	4/18/2018 11:48:52 AM
Method	tune-med_AP.m	Operator	a.piggott
Sample Name		Instrument	micrOTOF 213750.00232
Comment			

Acquisition Parameter

Source Type	ESI	Ion Polarity	Positive	Set Nebulizer	0.8 Bar
Focus	Not active			Set Dry Heater	180 °C
Scan Begin	100 m/z	Set Capillary	4500 V	Set Dry Gas	5.0 l/min
Scan End	1000 m/z	Set End Plate Offset	-500 V	Set Divert Valve	Source

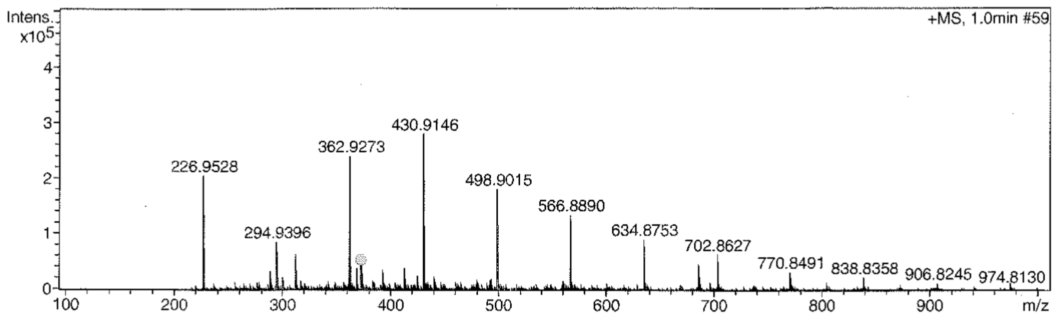

Meas. m/z	#	Ion Formula	m/z	err [ppm]	mSigma	# Sigma	Score	rdb	e⁻ Conf	N-Rule
373.1995	1	C20H30NaO5	373.1985	2.7	63.2	1	100.00	5.5	even	ok

Figure S52. HR-ESIMS data for cacolic acid B (**14**)

Mass Spectrum SmartFormula Report

Analysis Info

Analysis Name	D:\Data\s.khushi\CMB-03404-1-2-5-2.d	Acquisition Date	4/18/2018 12:59:59 PM
Method	tune-med_AP.m	Operator	a.piggott
Sample Name		Instrument	micrOTOF 213750.00232
Comment			

Acquisition Parameter

Source Type	ESI	Ion Polarity	Positive	Set Nebulizer	0.8 Bar
Focus	Not active			Set Dry Heater	180 °C
Scan Begin	100 m/z	Set Capillary	4500 V	Set Dry Gas	5.0 l/min
Scan End	1000 m/z	Set End Plate Offset	-500 V	Set Divert Valve	Source

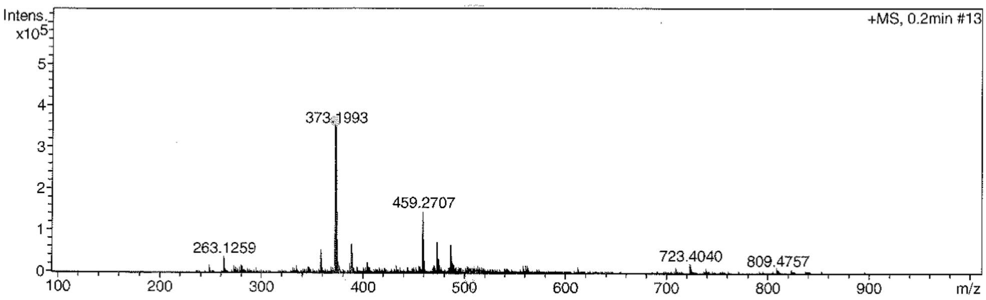

Meas. m/z	#	Ion Formula	m/z	err [ppm]	mSigma	# Sigma	Score	rdb	e⁻ Conf	N-Rule
373.1993	1	C20H30NaO5	373.1985	2.1	24.5	1	100.00	5.5	even	ok

Figure S53. HR-ESIMS data for cacolic acid C (**15**)
